# Ediacaran origin and Ediacaran-Cambrian diversification of Metazoa

**DOI:** 10.1126/sciadv.adp7161

**Published:** 2024-11-13

**Authors:** Emily Carlisle, Zongjun Yin, Davide Pisani, Philip C. J. Donoghue

**Affiliations:** ^1^ Bristol Palaeobiology Group, School of Earth Sciences, University of Bristol, Life Sciences Building, Tyndall Avenue, Bristol BS8 1TQ, UK.; ^2^ State Key Laboratory of Palaeobiology and Stratigraphy, Nanjing Institute of Geology and Palaeontology, Chinese Academy of Sciences, Nanjing 210008, China.; ^3^ CAS Center for Excellence in Life and Paleoenvironment, Nanjing 210008, China.

## Abstract

The timescale of animal diversification has been a focus of debate over how evolutionary history should be calibrated to geologic time. Molecular clock analyses have invariably estimated a Cryogenian or Tonian origin of animals while unequivocal animal fossils first occur in the Ediacaran. However, redating of key Ediacaran biotas and the discovery of several Ediacaran crown-Metazoa prompt recalibration of molecular clock analyses. We present revised fossil calibrations and use them in molecular clock analyses estimating the timescale of metazoan evolutionary history. Integrating across uncertainties including phylogenetic relationships, clock model, and calibration strategy, we estimate Metazoa to have originated in the early Ediacaran, Eumetazoa in the middle Ediacaran, and Bilateria in the upper Ediacaran, with many crown-phyla originating across the Ediacaran-Cambrian interval or elsewise fully within the Cambrian. These results are in much closer accord with the fossil record, coinciding with marine oxygenation, but they reject a literal reading of the fossil record.

## Teaser

Molecular clock analyses based on revised fossil interpretations and rock dating estimate an Ediacaran origin of animals.

## INTRODUCTION

The timescale of animal diversification has been the subject of controversy from the initial formulation of evolutionary theory (*1*). A century and a half of subsequent research has transformed understanding of the extent of geological time and the richness of the fossil record, but it has done little to dull the distinction between the Neoproterozoic and Phanerozoic fossil record of animals. To be sure, there are claims of fossil animals extending deep into the Neoproterozoic, including putative sponge body fossils at 890 million years ago (Ma) (*2*) and sponge biomarkers at 713 to 635 Ma (*3*, *4*), but the veracity of both records have been called into question (*2*, *5*–*9*). The Weng’an biota (~590 Ma) yields embryo-like fossils (*10*) that have been interpreted as metazoans, although the weight of evidence now favors a non-metazoan holozoan affinity (*11*–*15*). The age of the putative stem-ctenophore, *Eoandromeda*, has been redated from 582 Ma (*16*) to <560 Ma (*17*), and its ctenophoran affinity has been called into question (*18*–*20*). Fossil burrows may provide the first evidence for locomotion 565 Ma, but whether they were produced by stem- or crown-metazoans is debated (*21*). The strongest evidence for pre-Phanerozoic animals comes from the reinterpretation of Ediacaran rangeomorphs (574 Ma) as stem-eumetazoans (*22*), supporting a post-Gaskiers (~574 Ma) origin of crown-Eumetazoa, although there are no unequivocal records of bilaterians until the late Ediacaran appearance of trace fossils (*23*) reflecting animals with body plans capable of peristalsis (*24*). Nevertheless, there is evidence for a broad diversity of bilaterians in the earliest Cambrian, as evidenced by fossil Konservat-Lagerstätten such as the Kuanchuanpu biota [537 to 532 Ma; (*25*)] from which scalidophorans (*26**–**30*), mollusks (*31*), and chaetognaths (*31*) are known, along with cnidarians (*25*, *32*) and the speculative claim of a ctenophore (*33*). As well as directly demonstrating the existence of the respective phyla, these fossils also indirectly indicate the establishment of their successive sister lineages (*34*), corroborated by fossil evidence for most bilaterian phyla in younger Cambrian biotas such as the Sirius Passet (*35*), Chengjiang (*36*), and Burgess Shale (*37*) Lagerstätten. This sudden appearance of a fossil record of bilaterian animals lies at the core of the Cambrian explosion hypothesis, which suggests a sudden and dramatic diversification of bilaterian animals and their body plans within the early Cambrian or, at the very earliest, the latest Ediacaran (*38*). However, the alternative interpretation, that it reflects only a sudden appearance of more readily fossilizable animals (*39*), is at least equally as widely held. This view is supported by a dearth of Ediacaran sedimentary strata relative to the proceeding Cambrian (*40*, *41*), the paucity of stem-representatives to clades of phyla (*42*), the differential preservation potential of ancestral animals (*42*), and not least by molecular clock analyses that universally estimate an extensive pre-Phanerozoic history for Bilateria, Eumetazoa, and Metazoa (*43**–**45*).

Molecular timescales for early animal evolution have been the subject of robust criticism on a number of grounds, most recently based on the argument that hyper-diverse clades like arthropods require unusually high rates of diversification early in their evolutionary history that molecular clock methods cannot accommodate (*38*). Yet, the arthropod clade is one in which molecular clock estimates and the fossil record are in agreement, with paleontological estimates of total-group euarthropod origination at or after ~550 Ma (*46*) and a composite of molecular clock analyses yielding an estimate of 545.1 to 588.5 Ma (*44*). Furthermore, birth-death modeling of rapid diversifications yields estimates compatible with conventional molecular clock methods (*47*). However, the principal objection to many molecular timescales is based on a lack of corroboration from the fossil record (*48*) since some researchers expect molecular estimates of clade age to closely approximate fossil minima. At least in part, this criticism is illogical since the fossil record of morphology will always underestimate clade ages that reflect the genetic divergence of lineages. Morphological attributes can only accrue after divergence and they have to be fossilizable and sufficient in number to discriminate from ancestral characters asymmetrically lost in descendent lineages, lengthening the gap between genetic divergence and fossil evidence of it having occurred (*49*). Furthermore, fossil occurrences are not random; they are driven largely by the environmental tolerances of organisms and secular variations in the preservation of those environments in the rock record (*49*, *50*), leading to structural gaps in the fossil record (*42*). Hence, we contend that the greatest weakness of molecular clock methods is the interpretation of the fossil record used in calibrating molecular evolution to geologic time. Uncertainty in the interpretation of fossils and their ages propagates directly through to uncertain posterior age estimates (*44*, *51*). This uncertainty is amplified by the need to define both minimum and maximum age constraints on calibrated nodes within molecular clock analyses. Minima are based on the oldest crown representative of clades which, by definition, must be older. Maxima attempt to constrain how much older the clade may be, justified in one way or another on the basis of qualified negative evidence, such as records of out-group lineages in older strata, which demonstrate that the correct preservational conditions were obtained, and so the absence of in-group members may reflect the fact that they had not yet evolved (*49*, *52*). Maxima are often arbitrary but objective in the sense that they are defined on the basis of a well-studied and readily dated deposit. However, their definition encompasses contemporaneous and younger fossil deposits in which an absence of evidence must shift toward evidencing the increasing probability that the clade had not yet evolved. Reflecting this, maxima are extended to older ages with a tail distribution to allow for the possibility that they underestimate clade age but at low probability.

Thus, we contend that it is the broad span of time between calibration minima and maxima that has resulted in such uncertain age estimates for clades such as Metazoa, Eumetazoa, and Bilateria (*44*) rather than speculative claims of pathological properties of relaxed molecular clock methods (*38*, *48*, *53*). Here, we seek to minimize this uncertainty, in particular, by departing from the practice of establishing maxima such that they encompass the variable quality of fossil evidence and the broad diversity of interpretations of clade age that are based on it. This is effective if the aim is to integrate over uncertainty in the interpretation of the fossil record [e.g. (*45*)]; however, our aim here is to establish an integrative timescale for animal diversification that is based on a critical evaluation of the fossil record and its interpretations rather than an historical consensus view. This is timely since there have been several fundamental shifts in our understanding of the fossil record of early animal evolution, from revised geochronology, to fossil discoveries and the reinterpretation of old records. Two key Ediacaran biotas have been redated and found to be substantially younger than previously thought (*17*, *23*). The Lantian biota yields numerous exceptionally preserved algal macro-remains but no crown-animals (*54*, *55*). Hence, redating this biota from 635.5 Ma ± 0.6 million years (Myr) (*56*) to 602 ± 7 Myr (*55*) has broad implications. The Weng’an biota has also been revised from 609 ± 5 Ma (*57*) to 587.2 Ma ± 3.6 Myr (*17*), and reanalysis of its animal embryo–like fossils has led to their reinterpretation as stem-Metazoa at best (*10*, *13*, *58*) but more likely non-metazoan holozoans (*11*, *12*). Together, these discoveries demonstrate that there is no evidence to suggest that crown-animals evolved before 590.8 Ma, whereas reinterpretation of *Charnia masoni* (and by implication, other rangeomorphs) as a stem-eumetazoan indicates that crown-metazoans were present by 574 Ma (*22*). The cnidarian *Auroralumina attenboroughii* attests to the existence of crown-Eumetazoa by 561 Ma (*59*), and the discovery of a diversity of bilaterians in the earliest Cambrian Kuanchuanpu Formation (*60*, *61*) evidences their diversification before 537 to 532 Ma (*25*).

Revisions of the age of key fossil biotas and the affinities of the organisms they contain have broad implications for the recalibration of molecular evolutionary timescales. Here, we revise the fossil calibrations for the major metazoan clades and conduct a molecular clock analysis to establish the impact of these revised calibrations on the timescale of metazoan diversification. In so doing, we analyze fixed tree topologies in a Bayesian relaxed-clock node-calibrated dating framework. Our results suggest that Metazoa originated in the Ediacaran, in contrast to previous analyses that indicated an older origin. We performed sensitivity analyses that show that our results are not affected by phylogenetic uncertainty in animal relationships, the use of different [independent (IR) and autocorrelated (AC)] rate models to relax the molecular clock assumption, and the probability densities used to model our calibrations. Our results demonstrate that previous estimates of the timescale of animal diversification were old and uncertain because the underpinning fossil calibrations were broad and uninformative, reflecting the broad diversity of interpretations of the fossil evidence. Our revised timescale is much more precise and in closer agreement with clade age minima, achieved in large part as a consequence of our critical reinterpretation of the fossil record in establishing constraints on divergence time estimation, which is necessarily limiting on our evolutionary timescale and the interpretations based on it.

## RESULTS

### Fossil calibrations

Forty-six nodes were calibrated on the basis of the latest fossil and geological data. We discuss here the calibrations used for Metazoa, Eumetazoa and Bilateria, as these are among the most contentious and impactful in estimating the timescale of metazoan diversification; the remainder can be found in the Supplementary Materials.

#### Crown-Metazoa

Rangeomorphs such as *Charnia masoni* are the first definitive evidence of crown-group metazoans. These have recently been reinterpreted as stem-group eumetazoans with possible sensory and contractile capabilities but neither muscles nor a nervous system (*22*). The earliest occurrence of *Charnia* is in the Drook Formation of Mistaken Point, Newfoundland, dated to 574.17 Ma ± 0.66 Myr (*62*). The minimum calibration can therefore be set at 573.51 Ma. For the soft maximum calibration, we use the Lantian biota, which yields numerous exceptionally preserved algal macrofossils but no credible claim for crown-Metazoa (*54*, *63*). The Lantian biota has been redated to 602 Ma ± 7 Myr (*55*), yielding a soft maximum calibration of 609 Ma.

#### Crown Eumetazoa

The recently described crown-group cnidarian *Auroralumina attenboroughii* is used as the minimum calibration for crown-group Eumetazoa. *Auroralumina* is interpreted as a thecate cnidarian with tetraradial periderm and was recovered as a stem-group medusozoan in a phylogenetic analysis (*59*). *Auroralumina* provides a minimum calibration age of 561.1 Ma; although more recent U-Pb–derived data provide a date of 556.6 Ma ± 6.4 Myr (*64*) for this formation, this date has high uncertainty and entirely encompasses the original, more precise date of 563 Ma ± 1.9 Myr (*65*). The soft maximum is based on the Weng’an biota, which is composed of exceptionally preserved fossil algae and embryo-like fossils that some have suggested may be stem-metazoans (*10*, *58*), although they are more likely non-metazoan holozoans (*11*, *12*, *66*), and there is certainly no evidence of stem- or crown-eumetazoans. The Weng’an biota was recently redated to 587.2 Ma ± 3.6 Myr (*17*), yielding a soft maximum clade age calibration of 590.8 Ma.

#### Crown Bilateria

The earliest evidence for crown Bilateria is equivocal; Ediacaran *Kimberella* has been interpreted as a stem-mollusk due to putative radula feeding traces, but this is disputed and *Kimberella* has also been interpreted as a stem-coelenterate (*44*, *67*, *68*). Another candidate for the oldest bilaterian is the putative chaetognath *Redkinia* (*69*, *70*); however, this is found only as fragmentary fossils that occur in isolation, making it difficult to establish its affinity and age (*70*). For this reason, we use the stem-gastropod *Aldanella yanjiahensis* (sometimes synonymized with *Aldanella attleborensis*) as the basis of the minimum age calibration for crown Bilateria. *Aldanella* is a dextrally coiled mollusk and indubitably sits within the stem of Gastropoda (*31*, *71*). *Aldanella* is found in the *Anabarites trisulcatus-Protohertzina anabarica* Assemblage Biozone, dated between 537 and 532 Ma (*72*), yielding a minimum calibration of 532 Ma. For the maximum calibration, we again use the Weng’an biota.

### Effective priors

**Fig. 1.**
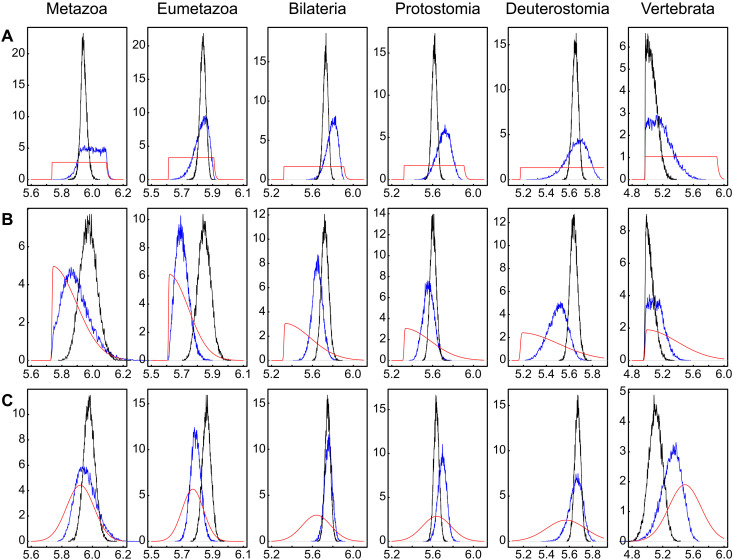
Effective priors, posterior age estimates and specified calibrations. Comparison of effective priors (blue), posterior age estimates (black), and specified calibrations (red) for the six major metazoan nodes. (**A**) Uniform calibrations, (**B**) Skew normal calibrations, and (**C**) normal calibrations. The *x*-axis scale is in 100 Myr.

Specified calibrations are affected by the topology of the tree used because a node cannot be older than its ancestors. The effective priors can therefore be different from the specified priors (calibrations) following the truncation of some of the calibration densities (*73**–**75*). Analyzing the tree without molecular data provides an estimate of the effective priors. Comparison of the specified and effective priors demonstrates that although in many cases, the effective prior is much narrower than the specified prior, they still overlap substantially (Fig. 1, Table 1 and figs. S1 to S3). The effective maximum for the metazoan node under the uniform calibration scheme and with the AC rate model (which assumes rate heritability, as opposed IR model which does not assume rate heritability) slightly exceeds the specified soft maximum but still falls within the soft 2.5% tail of the uniform distribution, with a specified calibration of 609 to 573.51 Ma and an effective prior of 610.1 to 588 Ma. Similar shifts from the specified to the effective prior are seen under the skew-normal and uniform calibration schemes using both AC and IR rate models. The eumetazoan node has a narrower effective calibration (590.2 to 573.7 Ma) than the specified calibration (590.8 to 561.1 Ma). For the bilaterian node, the effective priors (588.7 to 568.9 Ma) are slightly narrower and the minimum much older than in the specified calibration (590.8 to 532 Ma). This does not preclude a younger posterior estimate for the age of crown-Bilateria should the molecular data prove informative of such. However, rather than raw stratigraphic data, the older minimum on the effective prior reflects the structure of the phylogeny and the uncertainty associated with the age of descendant clades.

**Table 1.** Comparison of user-specified priors (calibrations), effective priors, and posteriors. Although similar, the effective calibrations once phylogeny is considered differ from the user-specified calibrations. There is also an effect of the molecular data on the age, as seen in the posterior age estimates that do not precisely match the user-specified calibrations. Posterior age estimates are from tree one and the AC rates model. The nodes presented here are the same as in Fig. 1.

		Priors			Posteriors		
Clade	Scheme	Mean	Lower	Upper	Mean	Lower	Upper
Metazoa	Calibration	591.3	573.5	609.0			
	Uniform	599.1	588.0	610.1	594.3	590.2	598.6
	Skew normal	589.5	574.0	606.9	597.4	586.8	608.3
	Normal	595.9	582.9	609.6	597.9	590.6	605.3
Eumetazoa	Calibration	576.0	561.1	590.8			
	Uniform	582.6	573.7	590.2	583.5	579.7	587.4
	Skew normal	570.1	562.3	578.3	584.5	576.2	592.7
	Normal	578.9	572.5	585.8	586.1	580.6	591.6
Bilateria	Calibration	561.4	532.0	590.8			
	Uniform	579.1	568.9	588.7	572.9	567.9	577.6
	Skew normal	564.9	554.6	575.3	572.3	565.1	579.3
	Normal	575.5	568.0	582.8	575.0	569.8	580.4
Protostomia	Calibration	561.4	532.0	590.8			
	Uniform	570.9	557.5	583.2	561.7	556.9	566.7
	Skew normal	556.1	545.6	566.7	560.5	554.6	566.5
	Normal	569.4	561.4	577.7	563.8	558.7	568.7
Deuterostomia	Calibration	554.1	517.3	590.8			
	Uniform	566.1	547.5	583.2	565.1	560.1	570.4
	Skew normal	549.8	532.6	564.8	563.8	557.4	570.3
	Normal	565.6	553.6	576.7	567.3	562.2	572.6
Vertebrata	Calibration	543.9	497.0	590.8			
	Uniform	518.1	496.9	544.7	506.5	496.8	519.0
	Skew normal	512.0	496.7	529.9	504.2	496.0	515.3
	Normal	531.4	505.1	555.5	510.4	492.8	527.5

Comparison of the posterior age estimates with the effective priors and specified priors shows that for most nodes, the molecular data are informative (Fig. 1, Table 1 and figs. S1 to S3). The posterior age estimates are not identical to the effective priors but fall within the bounds set by both the specified calibrations and the effective priors. This demonstrates that the molecular data are providing further information on the age of origin of the node not given by the fossil or topologically influenced priors. For a few nodes, the effective prior and posterior age estimates are closely comparable, suggesting that there has been little information gained from including the molecular data. However, for most nodes, there is enough difference to demonstrate that the molecular data are informative.

### Posterior age estimates

Since we did not statistically discriminate the fit of different clock models, we report the results of the averaged age estimates across all calibration schemes to account for the uncertainty, unless otherwise specified. Using the tree that reflects conventional views of animal relationships [i.e., monophyletic Deuterostomia, Xenacoelomorpha sister to Nephrozoa, and Porifera sister to all other animals, e.g., (*76*, *77*)], uniform calibration scheme and AC rates, the age of the metazoan crown-ancestor (598.6 to 590.2 Ma) falls firmly within the Ediacaran [635 to 538 Ma (*78*)]. Crown-Eumetazoa is estimated to have originated 587.4 to 579.7 Ma, followed closely by crown-Bilateria at 577.6 to 567.9 Ma. Nephrozoa, Deuterostomia, Ecdysozoa, Protostomia, and Spiralia are all estimated to have originated within the late Ediacaran, between 574.2 and 553.4 Ma. Most metazoan phyla are estimated to have originated within the Cambrian, or the 95% higher posterior densities (HPDs) span the Ediacaran-Cambrian boundary, except for Cnidaria, Chordata, and Xenacoelomorpha, which are estimated to have originated in the latest Ediacaran.

### Clock models

**Fig. 2.**
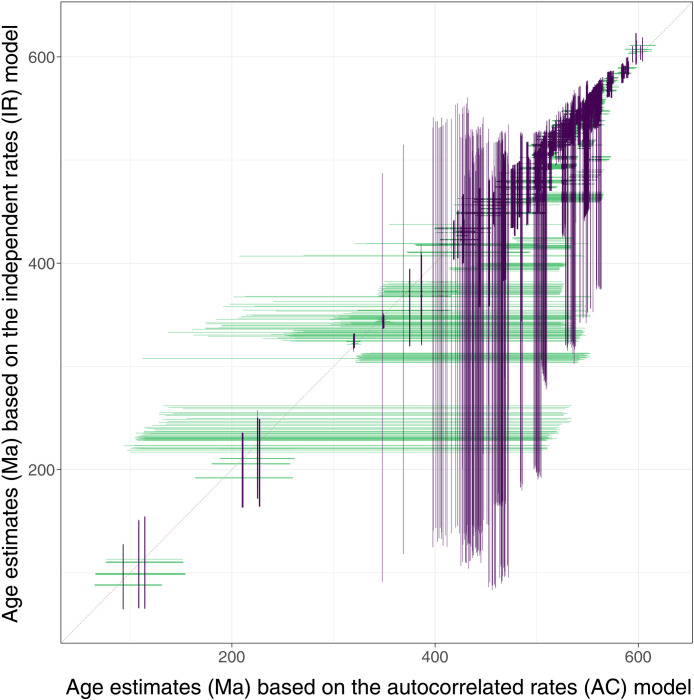
Comparison of the age estimates from AC and IR rate models. Comparison of the 95% HPD posterior age estimates for each node under AC and IR rates. In purple are the IR results for each node, and in green are the AC results for each node for all trees and calibration schemes; the 95% HPDs for the same nodes should intersect at the intercept if the results from the two models are the same. The plot shows that while the alternative clock rate models yield comparable ages for the oldest and youngest clades, the AC rate model consistently estimates older ages for clades of intermediate age.

There is very little difference between the results under the IR model and the AC model, although age estimates under the IR model have wider 95% HPD intervals and they are slightly older under the uniform calibration density scheme (Fig. 2 and fig. S1 to S3). Maximum age estimates were more likely to exceed the specified soft maximum calibration in analyses using the IR model. However, overall age estimate differences were minimal, with node age estimates overlapping substantially with those obtained under the AC model. We therefore report results averaged across both models, unless otherwise specified.

### Calibration densities

**Fig. 3.**
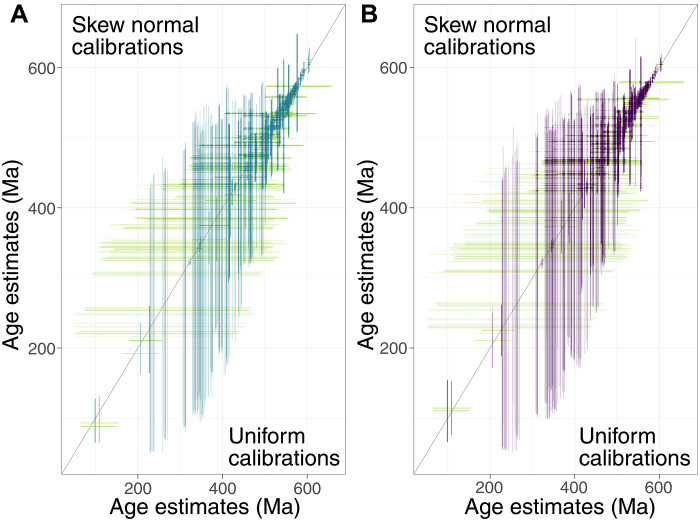
Comparison of the age estimates from the three calibration schemes. (**A**) Comparison of the 95% HPD posterior age estimates using skew-normal (*y* axis) versus uniform (*x* axis) calibration densities. (**B**) Comparison of the 95% HPD posterior age estimates using normal (*y* axis) versus uniform (*x* axis) calibration densities. The 95% HPDs for the same nodes should intersect at the intercept if the results from the competing calibration densities are the same. The figure shows that the competing calibration densities yield comparable clade age estimates.

While we used the same minimum and maximum age constraints in all of our analyses, the main analysis used a uniform distribution to span these constraints. This calibration scheme reflects agnosticism as to when clades originated between minimum-maximum calibration bounds. We also used two nonuniform distributions to explore their impact on divergence time estimation. The skew-normal calibration scheme reflects an optimistic interpretation of the fossil record, assuming that the minimum calibration is close to the true age of the clade. The normal calibration scheme is more pessimistic, reflecting a view that fossil calibration minima are a poor approximation of clade age. Overall, age estimates varied little between the schemes, with the minimum age for Metazoa slightly younger under the skew-normal calibration density scheme (3 Myr younger, averaged across tree topology and rates models; Fig. 3 and tables S1 to S3). The maximum age estimate is younger under the uniform calibration compared with the skew-normal and normal age estimates when averaged across all trees and rates models, although this difference is more pronounced for the metazoan and eumetazoan nodes. There is greater uncertainty in the age estimates when using the skew-normal and normal calibrations, as seen in larger 95% HPD intervals. Maximum age estimates under the skew-normal and normal calibration schemes are also more likely to violate the maximum calibration in the metazoan and eumetazoan nodes under the IR model. Maximum age estimates exceeding the calibrations on these nodes is more pronounced in the trees with Ctenophora as sister group to other metazoans. In these trees, the difference between the maximum age estimate under the uniform calibration scheme (averaged across trees and rate models) is 12 Myr for the skew-normal calibration scheme and 6 Myr for the normal calibration scheme. The maximum age estimate for the metazoan node exceeded the soft maximum calibration of 609 Ma for all trees under the IR model and for the trees with Ctenophora sister under the AC model, ranging from less than 1 to 19 Myr for Ctenophora sister trees with the IR model and skew-normal calibration scheme. For the eumetazoan node, the maximum age estimate exceeded the soft maximum calibration of 590.8 Ma by at most 3 Myr and only under the skew-normal and normal calibration schemes but for all tree topologies.

### Tree topologies

**Fig. 4.**
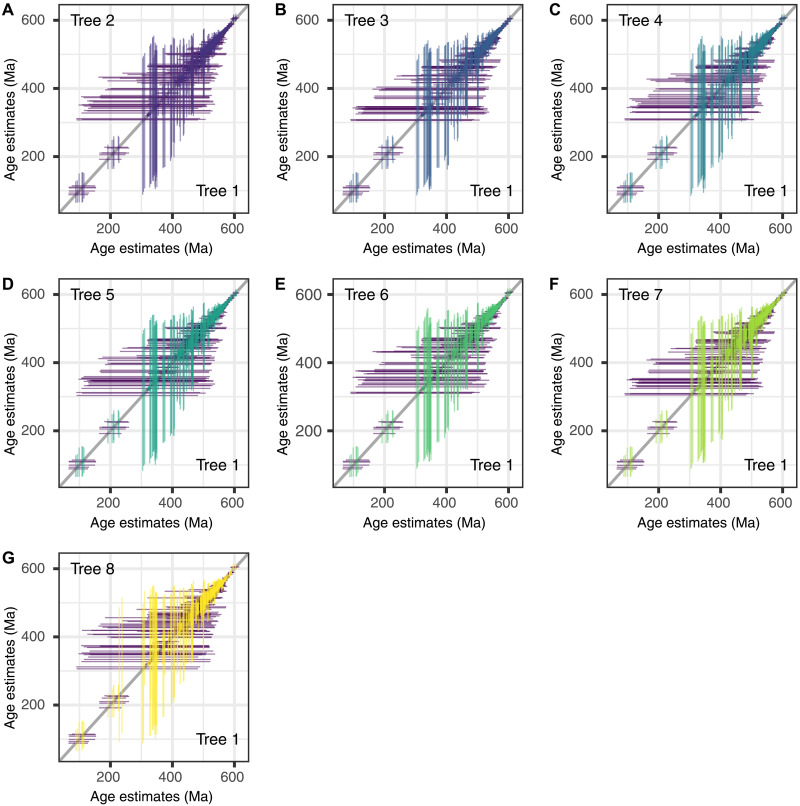
Comparison of the age estimates from the tree hypotheses. (**A** to **G**) Comparison of the 95% HPD posterior age estimates under each of the eight tree hypotheses, compared with a conventional tree (tree 1 in fig. S2) in which Porifera is sister to all other Metazoa, Xenacoels are sister to Bilateria and monophyletic deuterostomes. (A) Tree 2, which differs in recognizing Ctenophora sister. (B) Tree 3, which differs in recognizing Xenacoelomorpha grouped with Ambulacraria (Xenambulacraria). (C) Tree 4, which differs in recognizing Ctenophora sister and Xenambulacraria. (D) Tree 5, which differs in recognizing deuterostome paraphyly. (E) Tree 6, which differs in recognizing deuterostome paraphyly and Ctenophora sister. (F) Tree 7, which differs in recognizing deuterostome paraphyly and Xenambulacraria. (G) Tree 8, which differs in recognizing Ctenophora sister, Xenambulacraria, and deuterostome paraphyly. The 95% HPDs for the same nodes should intersect at the intercept if the results from the competing tree topologies are the same. The plots show that the competing topologies yield comparable clade age estimates.

**Fig. 5.**
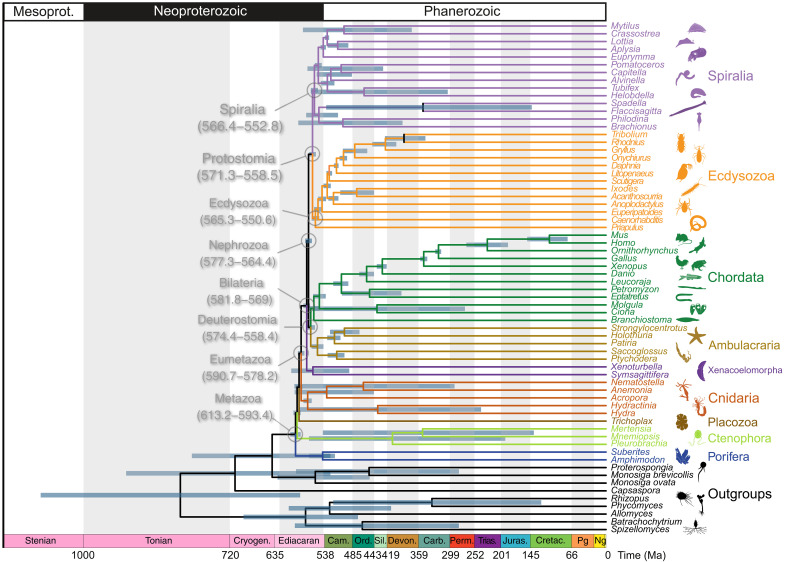
Metazoan clade age estimates. The conventional tree (tree 1 in fig. S2) with age estimates averaged across all trees, models, and calibration schemes used. Age estimates for nodes that are missing in the standard tree can be found in supplementary tables. 95% HPD intervals for major metazoan clades are labeled.

There are some differences among the divergence times estimated from the eight different topologies tested, although, overall, the difference is minimal (Fig. 4 and tables S1 to S3). The age estimate for crown-metazoans is slightly older in trees that have Ctenophora as the sister group to all other animals (age estimate of 616.1 to 595.2 Ma compared with 610.3 to 591.6 Ma, averaged across all rate models and calibration schemes; tables S1 to S3). This difference is not seen in the other nodes, including Eumetazoa. The age estimate for Bilateria is slightly older when Xenacoelomorpha is sister to Nephrozoa (582.6 to 569.8 Ma) rather than Ambulacraria (580.9 to 568.2 Ma), whereas Protostomia is slightly younger (570.5 to 557.7 versus 572.2 to 559.2 Ma), as is Deuterostomia (573.3 to 557.1 versus 575.5 to 559.6 Ma). When Deuterostomia is monophyletic, the age estimate for Protostomia is slightly older (572.8 to 559.8 Ma) than when Deuterostomia is paraphyletic (569.8 to 557.1 Ma), whereas Vertebrata is slightly older when Deuterostomia is paraphyletic (524.8 to 495.2 Ma) rather than monophyletic (522.8 to 494.6 Ma) (tables S1 to S3). Nevertheless, overall age estimates vary little across the alternative phylogenies. While we have reported results averaged across all trees, not all sets of relationships that have been proposed for Metazoa can be represented in a single, resolved, phylogeny. In our main figure, we present a conventional animal phylogeny in which Porifera is sister to all other animals, Deuterostomia is monophyletic, and Xenacoelomorpha is the sister of Nephrozoa (Fig. 5). The estimated ages of nodes that cannot be represented on this tree, such as Xenambulacraria, can be found in tables S1 to S3.

Summarizing the posterior age estimates for the nodes across all tests (model, calibration density scheme, and tree hypothesis), the age estimates remain substantially similar. The origin of Metazoa is estimated to be 613.2 to 593.4 Ma, while Eumetazoa is also mid-Ediacaran (590.7 to 578.2 Ma; Fig. 5).

## DISCUSSION

There is a long history of attempts to establish the age of origin of Metazoa. Most recent studies have suggested a Tonian [1000 to 720 Ma (*79*)] or Cryogenian [720 to 635 Ma (*79*)] origin for the clade (*44*, *45*) despite a lack of animal fossils before middle Ediacaran (*42*). Efforts to explain this discrepancy include suggestions of increased rates of evolution during the Cambrian, leading to the explosion of phenotypes observed in the fossil record (*80*), a long prehistory of animals with low energy requirements and little preservation potential (*81*, *82*). Our results suggest a short prehistory of metazoan evolution before its representation in the fossil record; animals originated in the Ediacaran shortly before fossils of crown-metazoans began to appear.

Much of the uncertainty in molecular clock analyses comes from fossil calibrations (*44*, *51*). Paleontological constraints on the deep divergences among Metazoa have been loose because of the challenge of interpreting negative evidence. However, as analysis of sites of exceptional preservation has proceeded, the plausibility that crown-metazoans existed contemporaneous with the Lantian and Weng’an biotas has diminished as it has become possible to reject claims of crown-metazoan affinity for their constituents. At the same time, records of metazoans and eumetazoans have become older, while the deposits that now appear to evidence the absence of metazoans, the Lantian and Weng’an biotas, have been redated as younger. In consequence, the calibrations on these deep nodes have become increasingly informative and influential in the ensuing posterior age estimates. It remains clear, however, that the fossil record should not be read literally.

### Phylogeny, rates models, and calibration density schemes

We examined the effects of different rates models, tree topologies, and calibration density schemes on the clade age estimates. Under the IR model, the ages of nodes tended to be more uncertain and would occasionally violate the maximum calibration set for the metazoan and eumetazoans nodes, but overall differences between node age estimates were minimal. The soft maximum age estimate is more likely to be exceeded in the metazoan and eumetazoan nodes when skew-normal and normal calibration density schemes are used. This may be due to higher probability placed around the mean in the normal calibration scheme, or because the effective priors on these nodes are so constrained. This suggests that the uniform calibration density may be allowing more precise estimates because, after truncation to ensure that ancestral nodes are older than their descendants, the bulk of the probability density remains close to the minimum bound. It is not possible to justify one calibration strategy in favor of others, but the differences in clade age estimates between calibration strategies are minor. Consequently, we report results averaged across all calibration strategies to ensure that the uncertainty in the fossil calibrations is captured in our summary evolutionary timescale.

Changes to the topology of the tree had little impact on the age estimates of the major nodes (Fig. 4 and tables S1 to S3). The minimum age of Metazoa was slightly older (~4 Myr when averaged across all rate model and calibration density schemes) in trees that had Ctenophora as the sister group to other animals. In addition, in these trees, the maximum age estimate for Metazoa exceeded the soft maximum calibration by ~7 Myr, whereas when Porifera is sister-group to other animals, the soft maximum is exceeded by ~1 Myr (averaged across all tests; this effect was more pronounced under skew-normal, normal, and IR conditions). In trees where Xenacoelomorpha is sister to Ambulacraria, the minimum and maximum age of Bilateria is 1 to 2 Myr younger, but no effect is seen on the age of Eumetazoa. The phylogenetic position of Xenacoelomorpha therefore does not have much of an effect on the age estimates of Metazoa and Eumetazoa. When Deuterostomia is paraphyletic, the age estimate for Protostomia is 1 to 2 Myr younger, while the age for vertebrates is 1 Myr older. Overall, however, there is very little difference in clade age estimates between the competing trees, indicating that the age estimates are robust to topological uncertainty. These results suggest that while aspects of metazoan phylogeny remained unresolved, this uncertainty does not propagate to the timescale of early animal evolution.

Regardless of tree topology, rate model, or calibration density scheme used, the minimum age estimate for the vertebrate node exceeded the minimum calibration by ~2 Myr. This violation was more pronounced under the skew-normal and normal calibration schemes. The node uses the minimum calibration age of 497 Ma based on the oldest conodont *Furnishina bigeminata* (*83*), but age estimates across the tree hypotheses, models, and calibrations are typically 0.1 to 8 Myr younger, with larger differences seen under the skew-normal, normal, and IR schemes. This suggests that the calibration used may be close to the true age of the node.

### An Ediacaran origin of Metazoa

Our results support an Ediacaran origin for crown-Metazoa, with an age estimate averaged across all analyses of 613.2 to 593.4 Ma (Fig. 5). We have chosen to average across the rates models, calibration density schemes, and topologies to capture analytic uncertainty. Similarly, crown-Eumetazoa is estimated to have diverged 590.7 to 578.2 Ma, and Bilateria, Deuterostomia and Protostomia diverged in the late Ediacaran (581.8 to 569 Ma, 574.4 to 558.4 Ma, and 571.3 to 558.5 Ma, respectively; Fig. 5); many of the crown phyla are estimated to have originated around the Ediacaran-Cambrian transition. Previous molecular clock analyses have suggested a much older age for Metazoa; some of the earliest analyses using a strict clock suggested an age of >900 Ma (*84**–**88*). With the inclusion of better calibrations (*63*, *89**–**91*) and Bayesian methods (*49*), molecular clock age estimates have drawn closer to fossil record minima, although still suggesting a Tonian or Cryogenian origin of crown-Metazoa (*43**–**45*, *92*, *93*). Our study used relaxed molecular clock models that are not very different from those of previous studies but with a revised set of fossil calibrations, yielding much younger divergence time estimates. This demonstrates the fundamental influence of calibrations in molecular clock analyses (*51*). Our calibrations, while they overlap with those used previously (*44*, *63*), are much more precise (and therefore informative), with maximum age calibrations informed by revised geological ages for the Ediacaran Lantian and Weng’an biotas, along with revised interpretations of their faunal and floral composition. Both have been extensively studied, and the absence of crown-metazoans, even under the most exceptional circumstances of fossilization, is informative (*11*, *12*). Combined with the absence of crown-metazoan fossils from younger strata, they provide for an arbitrary but objective basis on which our soft maximum constraints are based. Although some continue to argue for the presence of total-group metazoans in the Weng’an biota (*10*, *13*, *58*, *94*), this is not incompatible with our results.

Molecular clock estimates cannot be incompatible with the fossil evidence used in their calibration. However, they can be incompatible with alternative interpretations of those same fossil data and, as we have described, interpretations vary between those who accept that fossil minima can underestimate clade age by hundreds of millions of years versus others who believe the fossil record to be an accurate temporal archive of animal evolutionary history. Our divergence time estimates suggest that the fossil record of early animals is a close approximation of clade age. The earliest definitive evidence for crown-metazoans is from rangeomorphs such as *Charnia masoni*, which have been interpreted as stem-eumetazoans (*22*). These date to around 574 Ma, and the minimum age estimate for the Metazoan node is 593.4 Ma, suggesting 19 Myr of evolution before the clade appeared in the fossil record. This difference is not so much as to require an extensive, cryptic prehistory of crown-metazoans before their appearance in the known fossil record, but it still allows for the requirement that two sister lineages evolve distinctive morphological phenotypes from a common ancestor such that they can be distinguished as crown-, rather than stem-metazoans. Similar differences are seen in many of the other nodes, with the crown clades of Bilateria, Protostomia, and Deuterostomia all requiring a prehistory of 20 to 40 Myr before their appearance in the known fossil record. The pattern of sequential evolution of grades of organization, from crown-group metazoan to crown-group eumetazoan to crown-group bilaterian, is seen in parallel in both the molecular and fossil records (*38*). Marshall (*95*) argued that the gap between the true (molecular) origin time and the appearance of the recognizable lineage in the fossil record must be relatively small, less than the average species duration, which may be as little as 2 Myr or as much as 15 Myr (*96*). This compares well to our age estimates for Metazoa and Eumetazoa, which suggests that there may have been only a short interval of time in which it is necessary to invoke reduced preservation rates or secular bias in the rock record (*40*, *50*). However, for taxa without much of a fossil record, such as Nematoda, Nematomorpha, and Rotifera, the gap is much larger. Clades such as Bilateria, Protostomia, and Deuterostomia have a wider gap between divergence time estimate and fossil minimum, which may reflect difficulty in identifying early representatives of these clades in the fossil record. For Deuterostomia, in particular, the gap is ~40 Myr. However, the oldest fossil representatives of their sister lineage, the protostomes, are 20 Myr older than the oldest deuterostome fossils. Hence, the fossil record intrinsically requires a deuterostome ghost lineage that accounts for half of the discrepancy between the molecular clade age estimate and fossil minimum. Future work should focus on critical analysis of older candidate records of Bilateria, Protostomia, and Deuterostomia. For instance, with better characterization, the Ediacaran fossils *Kimberella* or *Redkinia* could inform calibration minima for both Bilateria and Protostomia, which would likely further diminish the temporal gap between the molecular clock clade age estimates and the fossil record minima (*67*, *70*, *97*, *98*).

Molecular clock analyses and the fossil record are not independent of one another. Molecular clock analyses are invariably calibrated using fossil evidence and, as that fossil evidence changes, the divergence time estimates change concomitantly. Our study is based on our interpretations of the fossil record (and its dating), which place only a small probability of crown-metazoans appearing much before the deposition of the Lantian Lagerstatten, currently dated to 602 ± 7 Myr (*55*), or the origination of eumetazoans and bilaterians much before the deposition of the Weng’an Lagerstatten, currently dated to 587.2 Ma ± 3.6 Myr (*17*). Relaxing the calibrations on these clades to encompass older, more speculative claims of animals from the Cryogenian (*3*) and Tonian (*2*) would, without question, result in concomitantly older estimates for the antiquity of animals [e.g., (*43**–**45*)]. However, these early and middle Ediacaran Lagerstatte have been subjected to intense scrutiny precisely because they constitute windows of exceptional preservation into shallow marine diversity during the interval in which earlier molecular timescales predicted the existence of diverse metazoans, eumetazoans, and bilaterians. That scrutiny has been rewarded with the evidence of non-metazoan holozoans, but no metazoans (*11**–**15*, *54*, *55*, *58*, *99*, *100*). Hence, on the basis of the available evidence, crown-animals are absent from these deposits because they had not yet evolved (*100*). The molecular clock is a method for rationalizing disparate paleontological, molecular, and phylogenetic data, their interpretations, along with evolutionary models, to obtain a holistic evolutionary timescale. However, it has to be accepted that changes to any of these variables will necessitate that we reanalyze the timescale of animal evolutionary history.

### Implications of an Ediacaran origin for Metazoa

Diverse intrinsic and extrinsic causal hypotheses have been proposed to explain the diversification of animals, but uncertainty in the interpretation of fossils and the age of the rocks in which they are recovered has precluded adequate tests (*44*). Recent revisions in geochronology (*17*, *23*) and reinterpretations of Ediacaran biotas (*14*, *15*, *22*, *59*) allow for more precise timescales that affect more stringent tests of proposed causal mechanisms. Most molecular clock analyses of animal diversification have concluded metazoans originated before or around the two “Snowball Earth” glaciation events, the Sturtian [~717 to 660 Ma; (*101*)] and Marinoan [~650 to 630 Ma; (*102*)] glaciations (*43**–**45*). This required rationalization of how early animals survived these catastrophic glaciations; there would have been limited areas of Earth with ice thin enough to allow for phototrophic primary production (*103*), requiring early metazoans to persist in isolated refugia (*103*) or in chemosynthetic communities (*104*). Our results indicate that neither of these scenarios was necessary as crown-metazoans originated long after these glaciation events, although these harsh environmental episodes may go some way to explain the low diversity of non-metazoan holozoans. Nevertheless, crown-metazoans and crown-eumetazoans appear to have emerged before the short-lived, and less severe, “Slushball Earth” Gaskiers glaciation episode [~580 Ma; (*103*, *105*)], although crown-bilaterians are estimated to have originated after the event.

Oxygenation of the biosphere has long been invoked as a prerequisite for the evolution of multicellular animals (*106*, *107*), extending to deterministic arguments that animal evolution was in some sense inevitable, delayed by a late rise in oxygen (*108*, *109*). Testing such hypotheses is challenging because the timing, molarity, and spatial extent of the oxygenation of the atmosphere and oceans remains poorly constrained. Further, confounding arguments suggest that, rather than being a consequence of a rise in oxygen, early animal evolution promoted marine oxygenation by filter feeding sponges breaking down cyanobacterially induced redox stratification of the oceans (*110*). In addition, rather than promoting complex animal body plans, ancestral animals may have had to adapt their development to tolerate oxygen (*111*). It is increasingly clear that the oxygenation of the marine environment was both temporally and spatially heterogeneous but that background levels did not show an appreciable rise until at least the middle to late Ediacaran (*112*), perhaps postdating the origin of crown-animals, -eumetazoans, and -bilaterians, but coinciding with the diversification of the animal phyla. Beyond the background, several Ediacaran ocean oxygenation events have been invoked as extrinsic drivers of metazoan diversification (*113*, *114*). In particular, the Shuram carbon isotope excursion [~575 to 565 Ma; (*115*)] has been correlated with increased ocean oxygenation (*116**–**120*) as well as extensive flooding, leading to the formation of shallow seas (*117*, *118*). The Shuram excursion ~575 to 565 Ma coincides with our estimated age for the divergence of crown-Bilateria, -Nephrozoa, -Protostomia, and -Deuterostomia. Metazoa and Eumetazoa are estimated to have originated shortly before the Shuram excursion, although the origination of Eumetazoa coincides with a smaller oxygenation event at ~580 Ma (*116*). Low ocean oxygenation may not limit the origination of metazoans such as sponges (*121*), but higher levels may be required for the formation of complex food webs (*122*). Our timescale suggests that the evolution of more complex bilaterians coincided with the increased episodic oxygenation of the oceans in the upper Ediacaran (*113*). Coincidence is not causation, but our timescale for animal diversification is certainly incompatible with conventional view that oxygenation of the biosphere delayed the diversification of animals since the fundamental clades that underpin animal diversity appear to have already diversified before long term increases in dissolved oxygen in the middle to late Ediacaran [e.g., (*119*)].

Our results suggest that there was a radiation of metazoans beginning in the middle Ediacaran, with all major phyla originating by the late Cambrian (Fig. 5), although this may have taken a little under 100 Myr based on the maximum and minimum age estimates for all of the phyla, suggesting the “Cambrian explosion” was more drawn-out than a literal reading of the fossil record would suggest. Although crown-Metazoa originated in the Ediacaran, many of the crown-phyla did not originate until the Cambrian; Euarthropoda, Echinodermata, Ctenophora, Hemichordata, Rotifera, and Chaetognatha likely originated in the Cambrian, while Chordata, Cnidaria, and Mollusca have late Ediacaran origins (Fig. 5). This coincides with transgression and expansion of shallow marine environments worldwide (*41*).

There is no shortage of rhetoric that pitches molecular clock methodology and the fossil record as opposed and incompatible, but, given that molecular clock analyses are usually calibrated using fossil evidence, this is self-evidently false. Molecular clock methods provide a means of interpreting the fossil record in establishing a timescale for evolutionary history and, hence, they are in tension only with alternative approaches to inferring evolutionary time from the fossil record. There can be no doubt that the fossil record requires interpretation since it can be demonstrated intrinsically to be an imperfect temporal archive of evolutionary history (*95*). Nevertheless, the fossil record remains integral to establishing evolutionary timescales, and, combined with molecular data and methods, fossil data provide the best means of disambiguating rates and times in molecular evolution. The accuracy and precision of molecular estimates of evolutionary timescales relies largely on the uncertainties within fossil calibrations (*123*). As we have shown, calibration uncertainty propagates through to posterior time estimates, and so the goal of timescale accuracy and precision requires informative fossil calibrations for critical nodes, facilitating effective estimates of the age of other clades that are more poorly represented in the fossil record. Thus, in a very real sense, the fundamental problem with molecular clock analyses is the fossil record (*51*, *124*) and, in particular, the limitations in our ability to interpret it. The development of mechanistic methods for deriving estimates of clade age from stratigraphic data (*125*) show promise as objective approaches to deriving informative fossil calibrations.

## MATERIALS AND METHODS

We used a Bayesian, node-calibrated, relaxed clock approach to date trees with a fixed topology. It was not our aim to find the topology of animal phylogeny, and so we constructed trees to reflect prior competing views of metazoan relationships. Thus, the trees were not estimated from the sequence data, but their branch lengths were estimated from the data as part of the dating analysis. The Bayesian relaxed clock approach was adopted because it allows us to reflect the uncertainty in the fossil calibration of node ages, as well as modeling rate variation among lineages. In particular, we used MCMCtree [v4.9 (*126*)] because its implementation of the approximate likelihood calculation allowed us to efficiently conduct experiments exploring the impact on timescale estimation of experimental variables such as rate model, tree topology, and calibration density, following the approach in (*44*).

### Sequence data

We added 11 taxa from (*127*) to an existing dataset (*44*), including three species of Ctenophora and a series of metazoan outgroups, allowing us to remove the metazoan crown node from the root of the tree to better estimate its age. Any locus not found in both sources (*44*, *127*) was discarded. This resulted in a dataset composed of 66 taxa combined into a single amino acid alignment composed of 30,031 amino acid positions from 203 nuclear coding genes.

### Tree hypotheses

Metazoan phylogeny is not yet fully resolved, especially the earliest sequence of branching. To examine the effect of phylogenetic uncertainty on the age estimates for metazoan clades, we tested eight tree topologies with the same number of tips but with the branching order modified to represent alternative resolutions of disputed clades (fig. S5). The trees represent alternative resolutions for the root of Metazoa, placing either Ctenophora (comb jellies) (*128**–**132*) or Porifera (sponges) (*127*, *133**–**138*) as the sister of all other animals. We also considered alternative hypotheses for the placement of Xenacoelomorpha, as sister to either Nephrozoa or to Ambulacraria (*138**–**141*). Last, we tested the effects of assuming Deuterostomia to be either monophyletic or paraphyletic (*140**–**143*). For comparisons, the tree with Porifera as sister to other animals, Xenacoelomorpha as sister to Nephrozoa, and monophyletic Deuterostomia was chosen as the standard tree (fig. S5, tree 1).

### Molecular clock analysis

Forty-six nodes were calibrated, updated from (*63*) (figs S1 to S4). Twenty-three minimum bound and 30 maximum bound calibrations were updated to reflect the latest improvements in our understanding of the fossil record and of the geological timescale. Minimum calibrations were chosen on the basis of the oldest recognized and widely accepted crown-group member of considered clades. Maximum calibrations were based on the youngest formation that yields close relatives of the clade of interest, but has no evidence of the clade itself, under the assumption that the clade would have been fossilized had it been present.

To test the robustness of the results, we examined three different calibration density schemes (fig. S6). First, we used calibrations with a 0.1% soft minimum bound and a 2.5% soft maximum bound added to a uniform distribution spanning the minimum and maximum specified calibration constraints. The resulting probability distributions representing agnosticism concerning the timing of divergence between minimum and maximum bounds, while also allowing for the divergence time to lie outside these bounds at low probability. To represent a more optimistic view of the fossil record, we used a skew-normal distribution to place higher probability near the minimum bounds, representing a prior view that the minimum constraint is a close approximation of clade age. Last, we used a normal distribution to represent a less optimistic view, placing higher probability for the age of origin at the midpoint between the minimum and maximum bounds.

Analyses were performed using MCMCtree v4.9 (*126*), with the likelihood calculated approximately, as is customary with large molecular alignment, and using the LG + G model of amino acid substitution (*144*). The evolutionary rate (mean substitution rate, or rgene_gamma) for each tree hypothesis was calculated on the basis of the topology and the calibration scheme used. The prior for the rate variance (σ^2^) was set to G (*1*, *10*), a gamma distribution with shape of 1 and scale of 10, to allow serious violation of the clock (*44*). The analysis was run for 20,000 iterations after a burn-in of 100,000 and with a sampling frequency of 1000. Five chains were run to ensure convergence, which was quantified by effective sample size values of greater than 200 and by comparing the posterior means. Converged chains were averaged to reach a consensus value for each node. Each tree topology was run under both the IR and AC rate models (*123*) for each calibration scheme. All age estimates, from priors to posteriors, are reported in terms of the 95% HPD rather than mean or median since simulations indicate that the 95% HPD has higher coverage probability (*145*).

## Supplementary Materials

### This PDF file includes:

Figs. S1 to S6 Tables S1 to S3 Supplementary calibration justifications References
