# Supplementary material for: Ediacaran origin and Ediacaran-Cambrian diversification of Metazoa
Source: Sci Adv. 2024 Nov 13;10(46):eadp7161. doi: 10.1126/sciadv.adp7161 (PMC11559618; doi:10.1126/sciadv.adp7161)
Supplement: Supplementary file 1 — Figs. S1 to S6 Tables S1 to S3 Supplementary calibration justifications References [file sciadv.adp7161_sm.pdf]

Science Advances
AAAS

Supplementary Materials for

Ediacaran origin and Ediacaran-Cambrian diversification of Metazoa

Emily Carlisle *et al.*

Corresponding author: Philip C. J. Donoghue, phil.donoghue@bristol.ac.uk

Sci. Adv. **10**, eadp7161 (2024)
DOI: 10.1126/sciadv.adp7161

This PDF file includes:

Figs. S1 to S6
Tables S1 to S3
Supplementary calibration justifications
References

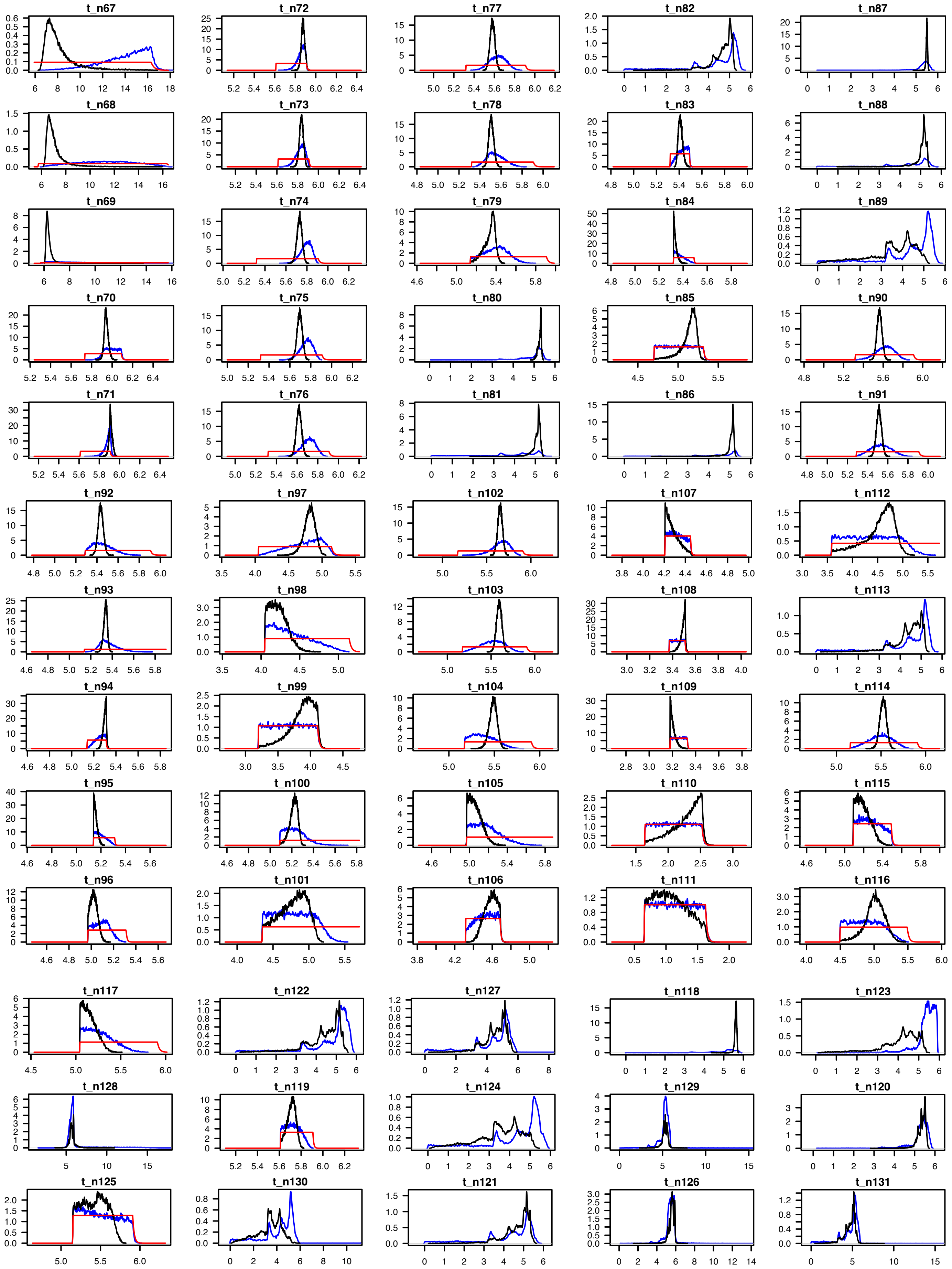
t_n67
t_n72
t_n77
t_n82
t_n87
t_n68
t_n73
t_n78
t_n83
t_n88
t_n69
t_n74
t_n79
t_n84
t_n89
t_n70
t_n75
t_n80
t_n85
t_n90
t_n71
t_n76
t_n81
t_n86
t_n91
t_n92
t_n97
t_n102
t_n107
t_n112
t_n93
t_n98
t_n103
t_n108
t_n113
t_n94
t_n99
t_n104
t_n109
t_n114
t_n95
t_n100
t_n105
t_n110
t_n115
t_n96
t_n101
t_n106
t_n111
t_n116
t_n117
t_n122
t_n127
t_n118
t_n123
t_n128
t_n119
t_n124
t_n129
t_n120
t_n125
t_n130
t_n121
t_n126
t_n131

Supplementary Figure S1: Calibrations, priors, and posteriors from the uniform calibration scheme.

In red are the uniform calibration curves, in blue the prior curves, and in black the posterior curves for the standard tree (tree 1). Nodes are numbered based on the standard tree.

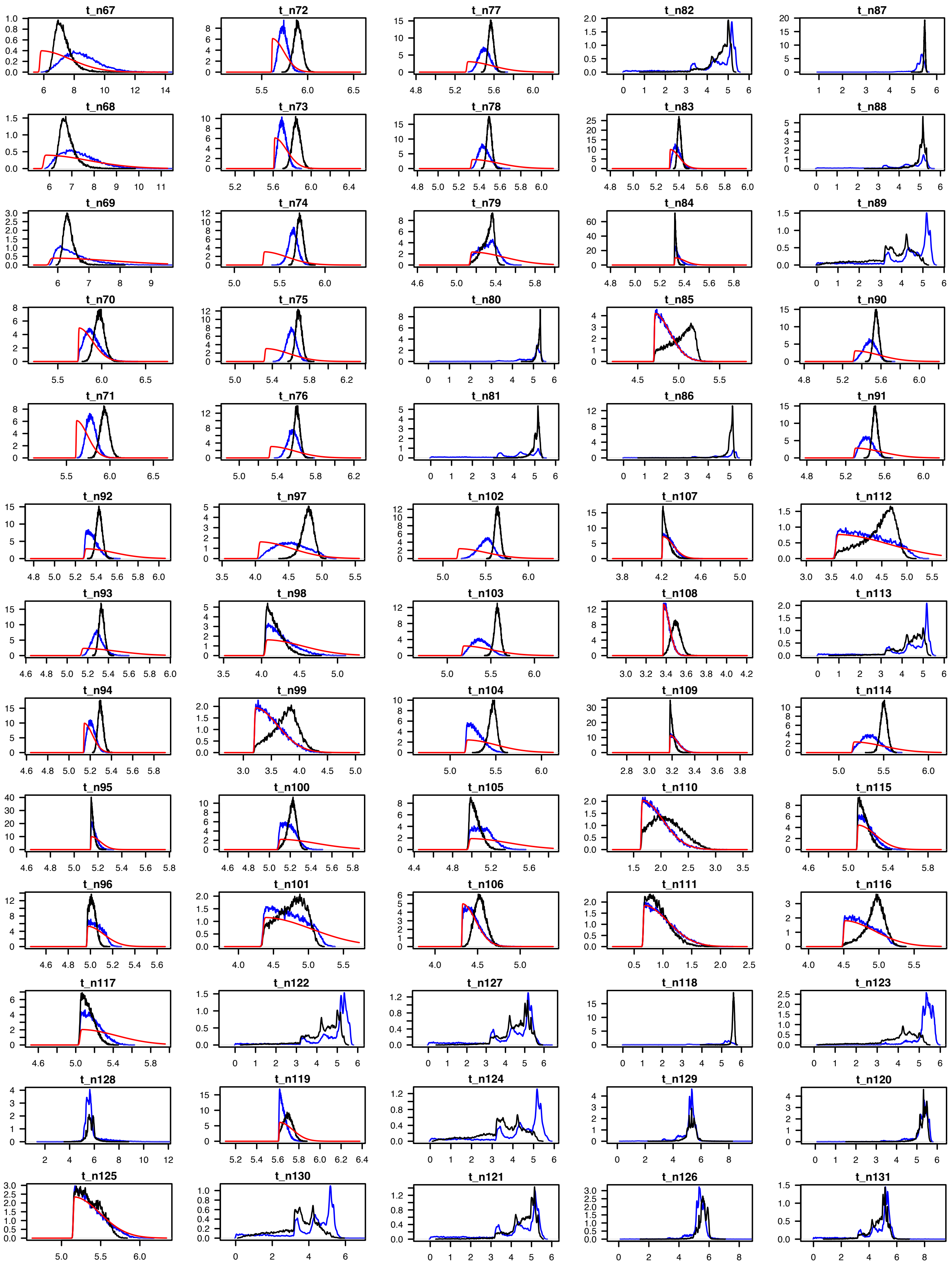
t_n67
t_n72
t_n77
t_n82
t_n87
t_n68
t_n73
t_n78
t_n83
t_n88
t_n69
t_n74
t_n79
t_n84
t_n89
t_n70
t_n75
t_n80
t_n85
t_n90
t_n71
t_n76
t_n81
t_n86
t_n91
t_n92
t_n97
t_n102
t_n107
t_n112
t_n93
t_n98
t_n103
t_n108
t_n113
t_n94
t_n99
t_n104
t_n109
t_n114
t_n95
t_n100
t_n105
t_n110
t_n115
t_n96
t_n101
t_n106
t_n111
t_n116
t_n117
t_n122
t_n127
t_n118
t_n123
t_n128
t_n119
t_n124
t_n129
t_n120
t_n125
t_n130
t_n121
t_n126
t_n131

Supplementary Figure S2: Calibrations, priors, and posteriors from the skew-normal calibration scheme. In red are the skew-normal calibration curves, in blue the prior curves, and in black the posterior curves for the standard tree (tree 1). Nodes are numbered based on the standard tree.

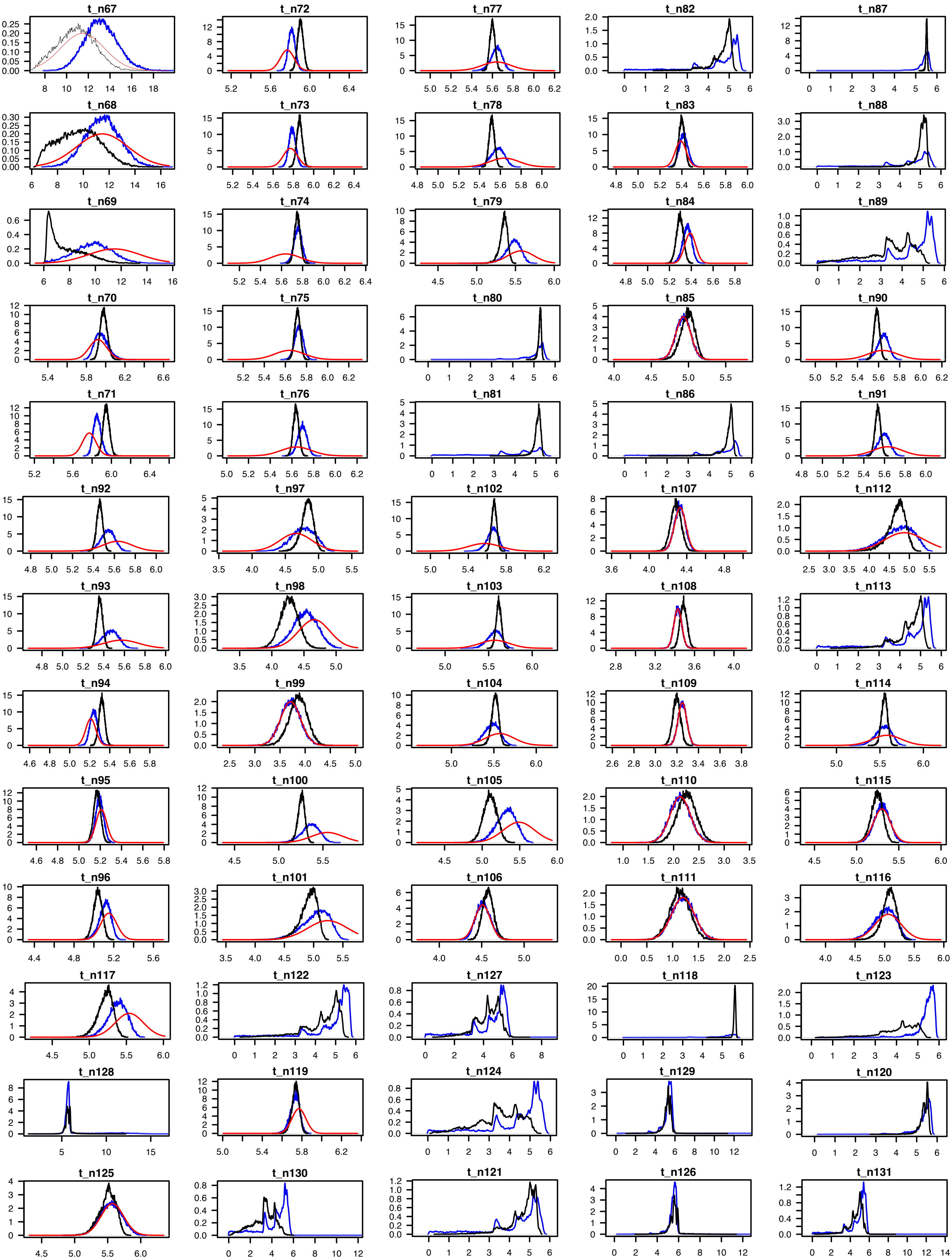
t_n67
t_n72
t_n77
t_n82
t_n87
t_n68
t_n73
t_n78
t_n83
t_n88
t_n69
t_n74
t_n79
t_n84
t_n89
t_n70
t_n75
t_n80
t_n85
t_n90
t_n71
t_n76
t_n81
t_n86
t_n91
t_n92
t_n97
t_n102
t_n107
t_n112
t_n93
t_n98
t_n103
t_n108
t_n113
t_n94
t_n99
t_n104
t_n109
t_n114
t_n95
t_n100
t_n105
t_n110
t_n115
t_n96
t_n101
t_n106
t_n111
t_n116
t_n117
t_n122
t_n127
t_n118
t_n123
t_n128
t_n119
t_n124
t_n129
t_n120
t_n125
t_n130
t_n121
t_n126
t_n131

Supplementary Figure S3*: *Calibrations, priors, and posteriors from the normal calibration scheme.

In red are the normal calibration curves, in blue the prior curves, and in black the posterior curves for the standard tree (tree 1). Nodes are numbered based on the standard tree.

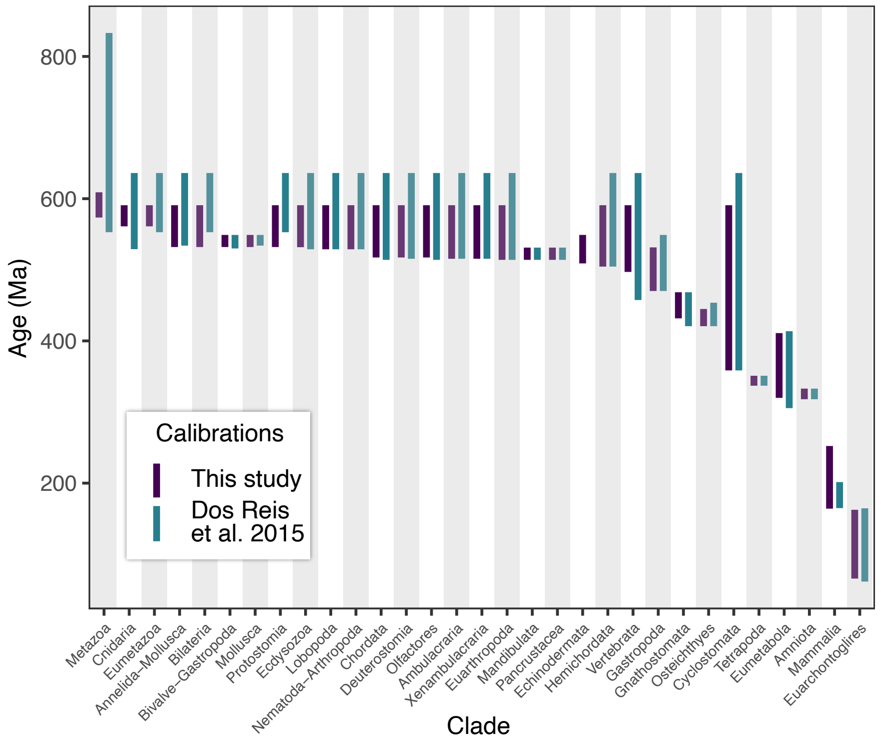

Age (Ma)
800
600
400
200
Calibrations
This study
Dos Reis
et al. 2015
Metazoa
Cnidaria
Eumetazoa
Annelida–Mollusca
Bilateria
Bivalve–Gastropoda
Mollusca
Protostomia
Ecdysozoa
Lobopoda
Nematoda–Arthropoda
Chordata
Deuterostomia
Olfactores
Ambulacraria
Xenambulacraria
Euarthropoda
Mandibulata
Pancrustacea
Echinodermata
Hemichordata
Vertebrata
Gastropoda
Gnathostomata
Osteichthyes
Cyclostomata
Tetrapoda
Eumetabola
Amniota
Mammalia
Euarchontoglires
Clade

Supplementary Figure S4: Comparison of calibration schemes. In blue are the calibrations for the major metazoan nodes used in this study, while in purple are those from dos Reis et al. (*44*). Although the calibrations overlap substantially, the ones used in this study are narrower.

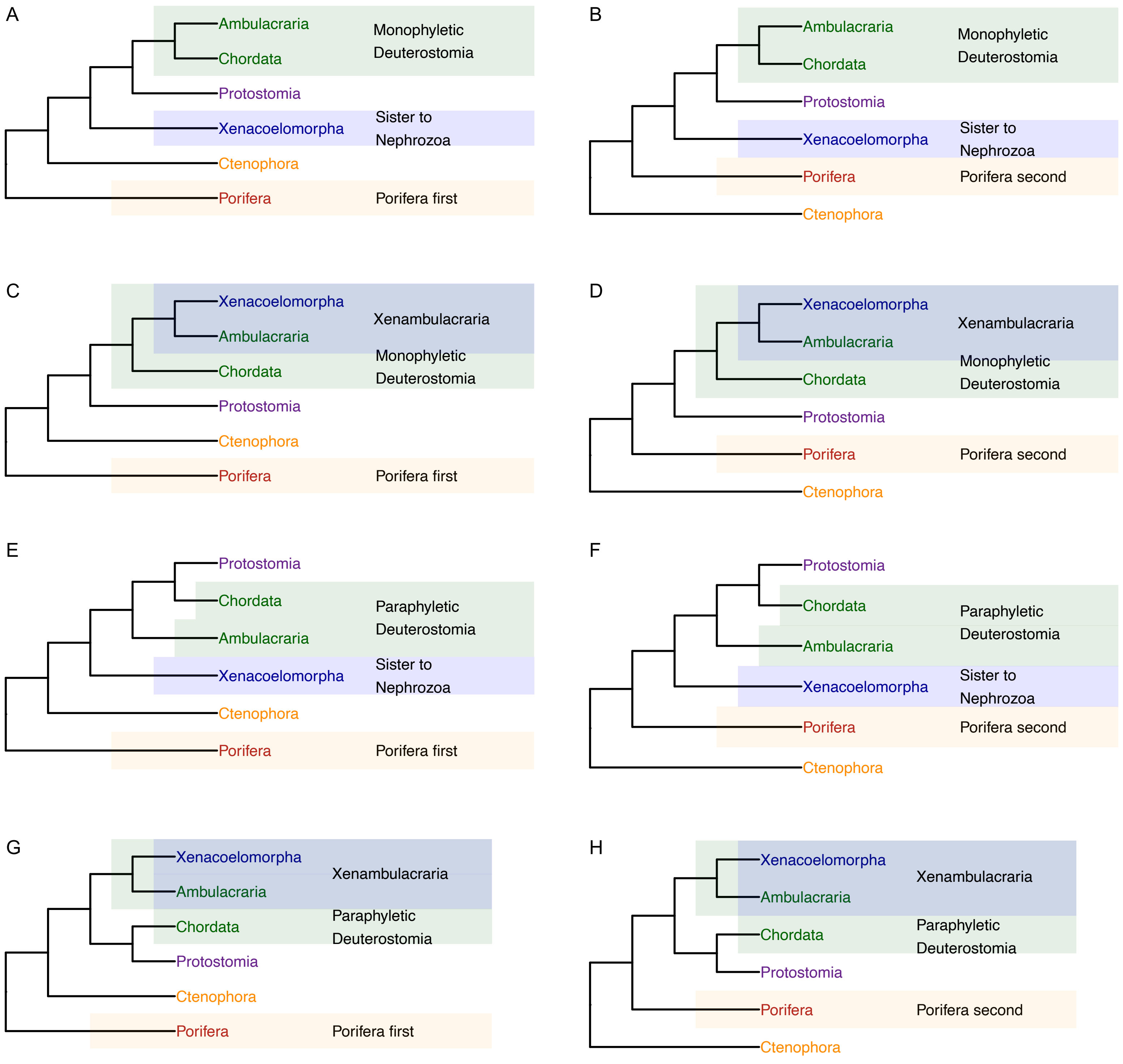
A
Ambulacraria
Chordata
Monophyletic Deuterostomia
Protostomia
Xenacoelomorpha
Sister to Nephrozoa
Ctenophora
Porifera
Porifera first
B
Ambulacraria
Chordata
Monophyletic Deuterostomia
Protostomia
Xenacoelomorpha
Sister to Nephrozoa
Porifera
Porifera second
Ctenophora
C
Xenacoelomorpha
Ambulacraria
Xenambulacraria
Chordata
Monophyletic Deuterostomia
Protostomia
Ctenophora
Porifera
Porifera first
D
Xenacoelomorpha
Ambulacraria
Xenambulacraria
Chordata
Monophyletic Deuterostomia
Protostomia
Porifera
Porifera second
Ctenophora
E
Protostomia
Chordata
Ambulacraria
Paraphyletic Deuterostomia
Xenacoelomorpha
Sister to Nephrozoa
Ctenophora
Porifera
Porifera first
F
Protostomia
Chordata
Ambulacraria
Paraphyletic Deuterostomia
Xenacoelomorpha
Sister to Nephrozoa
Porifera
Porifera second
Ctenophora
G
Xenacoelomorpha
Ambulacraria
Xenambulacraria
Chordata
Protostomia
Paraphyletic Deuterostomia
Ctenophora
Porifera
Porifera first
H
Xenacoelomorpha
Ambulacraria
Xenambulacraria
Chordata
Protostomia
Paraphyletic Deuterostomia
Porifera
Porifera second
Ctenophora

Supplementary Figure S5: Tree topologies used in this study. These tree hypotheses are based on previous studies (*128, 133, 138-140, 142, 143*). The standard tree is tree 1, with monophyletic Deuterostomia, Xenacoelomorpha sister to Nephrozoa and Porifera as sister to all other animals.

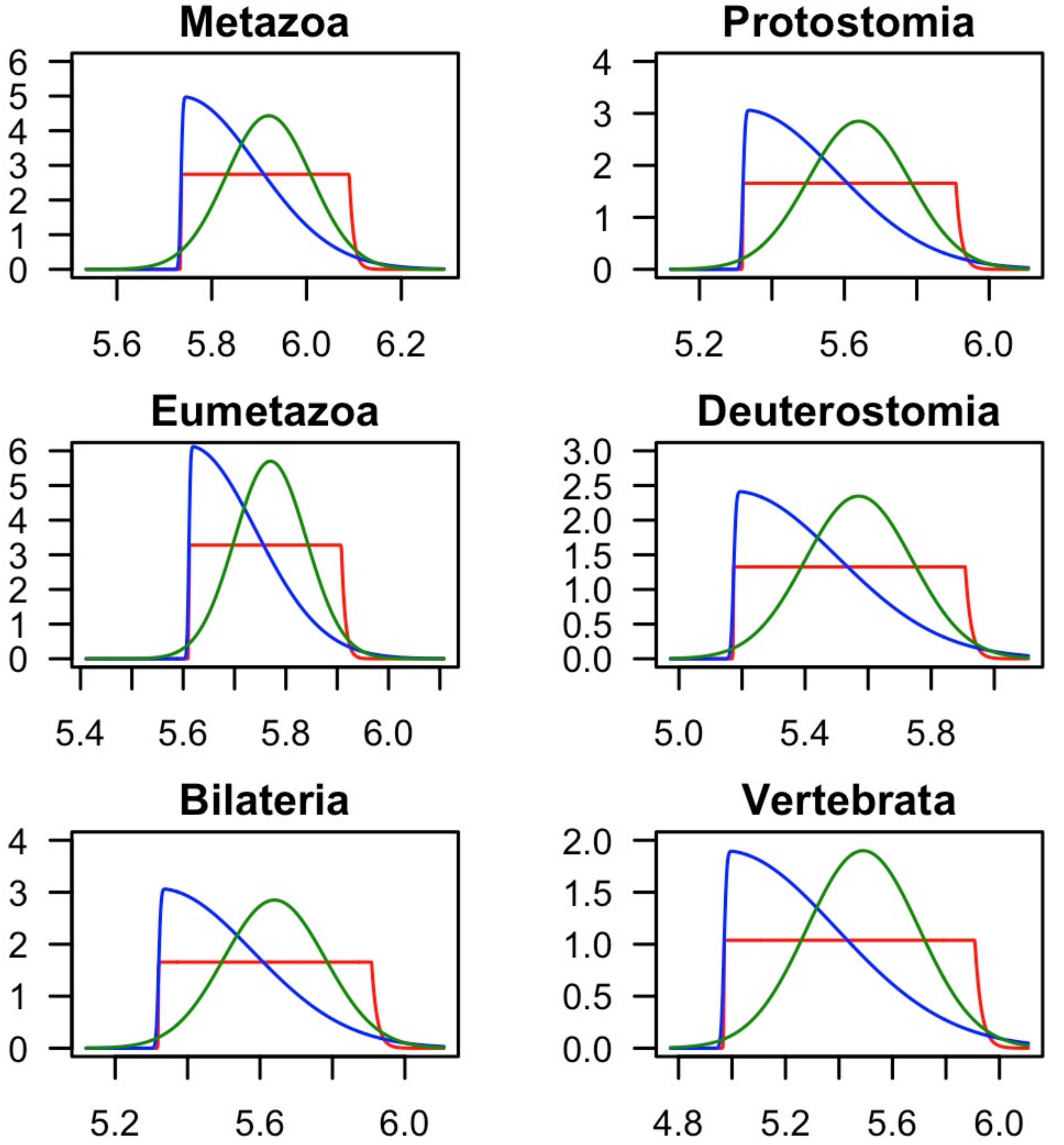

Metazoa
0
1
2
3
4
5
6
5.6
5.8
6.0
6.2
Protostomia
0
1
2
3
4
5.2
5.6
6.0
Eumetazoa
0
1
2
3
4
5
6
5.4
5.6
5.8
6.0
Deuterostomia
0.0
0.5
1.0
1.5
2.0
2.5
3.0
5.0
5.4
5.8
Bilateria
0
1
2
3
4
5.2
5.6
6.0
Vertebrata
0.0
0.5
1.0
1.5
2.0
4.8
5.2
5.6
6.0

Supplementary Figure S6: Comparison of the three calibration schemes for six major metazoan nodes. In red are the uniform calibrations for each node. In blue are the skew normal calibrations, placing a higher probability of an origin closer to the minimum calibration used. In green are the normal calibrations, with higher probability at the midpoint between the minimum and maximum calibrations. The x-axis scale is 100 million years.

Model	tree	Acari-Arenacea lower 95% HPD	Acari-Arenacea mean 95% HPD	Acari-Arenacea upper 95% HPD	Ambulacraria lower 95% HPD	Ambulacraria mean 95% HPD	Ambulacraria upper 95% HPD	Amniota lower 95% HPD	Amniota mean 95% HPD
AC	1	438.4	477.2	507.4	545.2	552.3	559.5	318.0	320.6
	2	438.9	477.2	507.8	545.3	552.5	559.7	318.0	320.7
	3	438.8	476.3	506.9	543.4	550.8	557.9	318.0	320.6
	4	438.7	476.2	506.8	543.6	550.9	558.1	318.0	320.6
	5	439.6	478.7	509.3	550.8	558.1	565.4	318.0	320.7
	6	440.2	478.9	510.2	550.9	558.2	565.7	318.0	320.7
	7	440.0	478.1	509.4	549.0	556.5	563.8	318.0	320.7
	8	439.1	478.2	508.4	549.1	556.7	564.0	318.0	320.7
IR	1	434.8	461.7	494.9	532.2	548.7	564.6	318.0	324.4
	2	434.9	461.9	495.4	531.9	548.7	564.6	318.0	324.5
	3	434.9	461.5	494.6	528.2	544.2	560.0	318.0	324.4
	4	434.9	461.8	495.2	527.9	544.2	559.7	318.0	324.4
	5	434.9	461.9	495.0	537.1	555.4	572.1	318.0	324.5
	6	434.9	462.1	495.7	537.0	555.4	572.1	318.0	324.5
	7	434.9	461.7	495.2	532.0	549.6	566.4	318.0	324.4
	8	434.9	461.9	495.5	531.8	549.5	566.9	318.0	324.4

Model	tree	Amniota upper 95% HPD	Annelida lower 95% HPD	Annelida mean 95% HPD	Annelida upper 95% HPD	Annelida-Mollusca lower 95% HPD	Annelida-Mollusca mean 95% HPD	Annelida-Mollusca upper 95% HPD	Bilateria lower 95% HPD	Bilateria mean 95% HPD
AC	1	325.4	520.5	533.3	542.9	546.2	550.7	555.2	567.9	572.9
	2	325.5	520.3	533.3	543.0	546.3	550.7	555.3	568.2	573.1
	3	325.3	519.9	533.4	543.0	546.8	551.3	555.9	565.8	570.8
	4	325.4	520.2	533.4	543.4	546.9	551.4	556.0	566.1	571.0
	5	325.6	521.1	533.2	542.2	545.0	549.3	553.7	568.6	573.4
	6	325.6	521.3	533.2	542.1	545.1	549.3	553.8	568.8	573.6
	7	325.5	521.0	533.3	542.5	545.5	549.8	554.2	566.5	571.4
	8	325.5	521.0	533.3	542.4	545.5	549.8	554.3	566.7	571.5
IR	1	331.9	513.9	528.5	544.6	544.9	553.9	562.9	573.3	579.2
	2	332.0	513.9	528.7	544.9	544.7	553.9	562.8	573.0	579.2
	3	331.9	513.9	528.8	545.6	545.8	555.4	564.7	572.4	578.6
	4	331.9	513.9	529.0	545.6	545.8	555.3	564.8	572.4	578.6
	5	332.0	513.9	527.9	543.4	543.9	552.4	560.9	573.7	579.6
	6	331.9	513.9	527.9	543.7	543.9	552.3	561.1	573.4	579.5
	7	331.9	513.9	528.1	544.0	544.6	553.6	562.5	572.9	579.0
	8	331.9	513.9	528.3	544.4	544.5	553.6	562.5	572.6	578.9

Model	tree	Bilateria upper 95% HPD	Bivalve-Gastropoda lower 95% HPD	Bivalve-Gastropoda mean 95% HPD	Bivalve-Gastropoda upper 95% HPD	Capitellid-Polychaete-Leech lower 95% HPD	Capitellid-Polychaete-Leech mean 95% HPD	Capitellid-Polychaete-Leech upper 95% HPD	Capsospora-Choanozoa lower 95% HPD
AC	1	577.6	532.0	533.6	536.7	509.5	526.7	539.7	621.3
	2	577.7	532.0	533.6	536.7	509.1	526.7	539.7	623.1
	3	575.5	531.9	533.7	536.8	508.4	526.6	539.6	621.5
	4	575.8	532.0	533.7	536.9	508.4	526.6	539.8	625.3
	5	578.1	532.0	533.5	536.3	511.2	527.0	539.5	621.4
	6	578.2	532.0	533.5	536.3	511.1	527.0	539.0	622.4
	7	576.1	532.0	533.5	536.4	510.9	527.0	539.7	621.4
	8	576.2	532.0	533.5	536.4	510.3	526.9	539.3	622.9
IR	1	585.2	532.0	535.0	540.1	426.5	495.2	538.5	692.2
	2	585.0	532.0	535.0	540.2	430.0	496.2	539.7	678.0
	3	584.6	532.0	535.1	540.3	430.0	496.1	540.1	697.8
	4	584.6	532.0	535.1	540.4	428.3	496.0	540.3	686.8
	5	585.3	532.0	534.9	539.9	425.6	493.9	537.8	692.9
	6	585.2	532.0	534.9	540.0	426.2	494.2	538.0	688.1
	7	584.8	532.0	535.0	540.1	426.0	494.4	537.7	691.6
	8	584.7	532.0	535.0	540.0	427.5	495.0	538.5	685.7

Model	tree	Capsospora-Choanozoa mean 95% HPD	Capsospora-Choanozoa upper 95% HPD	Chaetognatha lower 95% HPD	Chaetognatha mean 95% HPD	Chaetognatha upper 95% HPD	Choanoflagellata-Metazoa lower 95% HPD	Choanoflagellata-Metazoa mean 95% HPD
AC	1	712.5	876.2	107.3	349.7	510.6	606.8	638.8
	2	711.3	870.0	110.2	351.9	512.7	608.7	637.7
	3	711.9	872.5	105.1	346.4	507.8	606.0	639.1
	4	710.5	862.8	106.2	347.3	510.4	609.2	638.4
	5	711.8	886.4	115.8	355.0	514.8	605.5	637.9
	6	708.1	865.6	114.0	357.3	515.5	607.9	636.5
	7	710.9	873.4	108.3	351.3	512.2	606.2	638.4
	8	709.5	864.9	113.3	353.1	511.7	608.1	637.7
IR	1	989.5	1357.2	53.3	228.0	433.6	620.8	796.6
	2	969.8	1325.0	54.7	233.2	442.0	619.5	776.7
	3	987.9	1349.3	53.6	229.3	432.4	622.2	796.6
	4	969.9	1332.0	53.8	230.5	437.6	619.2	775.7
	5	994.3	1363.1	52.3	229.5	438.5	622.0	799.6
	6	975.1	1345.7	52.2	233.5	440.4	619.1	778.1
	7	988.5	1345.0	51.2	230.2	437.4	621.4	795.5
	8	972.2	1339.1	55.2	230.7	439.5	616.7	776.9

Model	tree	Choanoflagellata-Metazoa upper 95% HPD	Chordata lower 95% HPD	Chordata mean 95% HPD	Chordata upper 95% HPD	Cnidaria lower 95% HPD	Cnidaria mean 95% HPD	Cnidaria upper 95% HPD	Copepoda-Branchiopoda lower 95% HPD
AC	1	687.9	553.2	559.2	565.0	562.7	571.0	577.9	497.0
	2	677.7	553.4	559.4	565.3	563.0	571.2	578.2	497.0
	3	687.8	554.1	560.0	566.0	562.8	570.7	577.7	497.0
	4	678.5	554.1	560.2	566.1	563.0	570.9	577.8	497.0
	5	688.4	555.6	561.3	566.7	563.3	571.6	578.6	497.0
	6	676.0	555.9	561.4	567.1	563.3	571.8	578.7	497.0
	7	686.7	556.4	562.1	567.5	563.4	571.4	578.4	497.0
	8	678.0	556.5	562.2	567.7	563.5	571.5	578.4	497.0
IR	1	1046.8	533.8	550.8	566.5	561.1	567.6	576.6	496.9
	2	1010.9	533.5	550.7	566.5	561.0	567.7	576.6	497.0
	3	1045.4	536.4	553.6	570.4	561.1	567.4	576.2	496.9
	4	1003.2	535.6	553.5	569.7	561.1	567.5	576.3	496.9
	5	1052.7	536.8	554.7	570.5	561.1	567.8	576.9	497.0
	6	1018.3	536.6	554.6	570.7	561.1	567.8	577.0	496.9
	7	1041.4	538.2	556.8	572.9	561.1	567.6	576.5	496.9
	8	1008.8	537.8	556.7	572.8	561.1	567.7	576.5	496.9

Model	tree	Copepoda-Branchiopoda mean 95% HPD	Copepoda-Branchiopoda upper 95% HPD	Ctenophora lower 95% HPD	Ctenophora mean 95% HPD	Ctenophora upper 95% HPD	Ctenophora-other-Metazoa lower 95% HPD	Ctenophora-other-Metazoa mean 95% HPD
AC	1	502.5	508.0	174.0	406.4	536.6	587.8	591.3
	2	502.5	508.1	240.4	430.8	546.5	NA	NA
	3	502.3	507.9	173.9	403.9	536.2	587.8	591.2
	4	502.3	507.8	210.9	424.0	546.9	NA	NA
	5	502.7	508.4	205.4	416.0	538.2	588.0	591.3
	6	502.7	508.5	201.9	430.8	554.4	NA	NA
	7	502.6	508.3	190.0	410.5	536.5	588.0	591.3
	8	502.6	508.2	222.4	428.5	548.7	NA	NA
IR	1	503.3	511.6	132.8	337.1	532.0	587.9	591.3
	2	503.4	511.8	138.8	362.8	550.8	NA	NA
	3	503.3	511.6	133.2	336.9	531.2	587.9	591.3
	4	503.3	511.6	138.1	354.5	544.4	NA	NA
	5	503.4	511.7	138.2	346.8	539.9	587.9	591.3
	6	503.4	511.8	136.4	367.3	554.7	NA	NA
	7	503.3	511.6	136.1	339.2	536.4	587.8	591.3
	8	503.4	511.7	138.3	358.4	552.5	NA	NA

Model	tree	Ctenophora-other-Metazoa upper 95% HPD	Cyclostomata lower 95% HPD	Cyclostomata mean 95% HPD	Cyclostomata upper 95% HPD	Deuterostomia lower 95% HPD	Deuterostomia mean 95% HPD	Deuterostomia upper 95% HPD
AC	1	594.9	381.6	452.9	501.1	560.1	565.1	570.4
	2	NA	381.5	453.2	499.8	560.1	565.3	570.3
	3	595.0	381.2	452.9	499.5	561.1	566.3	571.3
	4	NA	382.5	453.1	500.0	561.3	566.4	571.5
	5	595.1	378.9	453.7	501.6	NA	NA	NA
	6	NA	380.2	453.7	503.1	NA	NA	NA
	7	595.1	380.5	453.3	501.8	NA	NA	NA
	8	NA	381.1	453.5	502.5	NA	NA	NA
IR	1	594.8	358.4	417.1	479.2	555.4	566.3	576.5
	2	NA	358.4	417.6	479.7	555.3	566.3	576.5
	3	594.9	358.4	417.2	478.8	559.9	570.0	579.6
	4	NA	358.4	417.5	479.1	559.8	570.0	579.6
	5	594.9	358.3	418.0	480.2	NA	NA	NA
	6	NA	358.3	418.5	480.5	NA	NA	NA
	7	594.8	358.4	417.6	479.8	NA	NA	NA
	8	NA	358.3	418.2	480.6	NA	NA	NA

Model	tree	Ecdysozoa lower 95% HPD	Ecdysozoa mean 95% HPD	Ecdysozoa upper 95% HPD	Echinodermata lower 95% HPD	Echinodermata mean 95% HPD	Echinodermata upper 95% HPD	Euarchontoglires lower 95% HPD
AC	1	551.2	556.2	561.1	508.9	519.7	533.0	65.8
	2	551.4	556.3	561.2	509.0	519.7	533.1	65.8
	3	552.3	557.2	562.3	508.9	518.7	531.3	65.7
	4	552.3	557.3	562.3	508.9	518.7	531.1	65.7
	5	549.9	554.7	559.5	509.0	523.6	539.8	65.8
	6	549.9	554.7	559.6	509.0	523.6	540.0	65.9
	7	550.6	555.4	560.2	509.0	522.0	537.2	65.7
	8	550.7	555.4	560.3	509.0	522.1	537.3	65.7
IR	1	550.7	560.8	570.8	508.9	519.8	536.5	65.6
	2	550.5	560.8	570.9	508.9	519.8	536.4	65.5
	3	552.6	563.1	573.3	508.9	518.2	532.8	65.6
	4	552.5	563.1	573.3	508.9	518.2	532.9	65.6
	5	548.7	558.7	568.2	509.0	522.0	540.7	65.6
	6	548.7	558.6	568.1	508.9	522.1	540.7	65.6
	7	550.3	560.6	570.2	508.9	520.1	537.1	65.6
	8	550.3	560.5	570.6	508.9	520.1	537.0	65.6

Model	tree	Euarchontoglires mean 95% HPD	Euarchontoglires upper 95% HPD	Euarthropoda lower 95% HPD	Euarthropoda mean 95% HPD	Euarthropoda upper 95% HPD	Eumetabola lower 95% HPD	Eumetabola mean 95% HPD
AC	1	108.3	153.9	529.9	533.6	537.0	344.9	386.7
	2	108.3	154.2	529.9	533.6	536.9	345.6	386.7
	3	108.2	153.9	530.0	533.7	537.0	346.0	386.8
	4	108.2	153.8	530.1	533.7	537.1	346.0	386.8
	5	108.6	154.2	529.6	533.3	536.7	345.4	386.5
	6	108.3	154.2	529.5	533.3	536.7	344.7	386.7
	7	108.4	153.9	529.8	533.5	536.9	344.2	386.6
	8	108.5	154.3	529.7	533.5	536.8	345.0	386.7
IR	1	98.9	150.6	525.5	534.6	543.9	321.9	368.0
	2	99.0	150.4	525.5	534.5	544.1	321.2	368.2
	3	98.1	149.7	525.9	535.2	544.8	321.3	368.0
	4	98.5	150.1	525.9	535.1	544.9	321.2	368.1
	5	99.2	150.8	524.9	533.9	542.8	321.5	368.1
	6	99.4	151.4	524.9	533.8	542.8	322.2	368.3
	7	98.7	150.3	525.6	534.5	543.8	320.8	368.2
	8	99.0	150.9	525.6	534.4	544.0	320.2	368.4

Model	tree	Eumetabola upper 95% HPD	Eumetazoa lower 95% HPD	Eumetazoa mean 95% HPD	Eumetazoa upper 95% HPD	Fungi-Holozoa lower 95% HPD	Fungi-Holozoa mean 95% HPD	Fungi-Holozoa upper 95% HPD	Gastropoda lower 95% HPD	Gastropoda mean 95% HPD
AC	1	415.2	579.7	583.5	587.4	647.7	817.6	1126.7	490.4	513.6
	2	415.4	580.0	583.8	587.5	648.1	814.8	1120.2	490.5	513.5
	3	415.6	579.3	583.3	587.1	646.5	813.5	1116.1	489.6	513.0
	4	415.7	579.7	583.5	587.3	649.6	812.0	1107.3	489.5	512.8
	5	415.8	580.0	583.8	587.6	645.9	814.5	1128.9	493.7	515.0
	6	415.0	580.3	584.0	587.7	648.7	812.3	1108.3	494.1	514.9
	7	415.0	579.7	583.5	587.4	645.2	809.7	1109.8	492.3	514.4
	8	415.6	579.9	583.7	587.4	647.0	812.4	1114.1	492.5	514.4
IR	1	408.0	579.3	584.2	588.9	814.8	1171.3	1598.3	470.1	491.7
	2	407.2	579.3	584.2	589.0	799.0	1156.6	1588.5	470.1	491.9
	3	407.3	579.0	584.0	588.7	814.7	1168.2	1589.7	470.1	491.5
	4	407.4	578.9	583.9	588.7	803.2	1154.1	1587.7	470.2	491.7
	5	407.6	579.6	584.5	589.1	823.3	1177.8	1604.2	470.1	491.8
	6	408.4	579.4	584.4	589.0	803.7	1162.2	1590.9	470.1	491.9
	7	406.7	579.3	584.2	588.9	820.3	1169.4	1596.3	470.1	491.7
	8	406.3	579.0	584.1	588.7	801.2	1158.5	1588.8	470.2	491.8

Model	tree	Gastropoda upper 95% HPD	Gnathifera lower 95% HPD	Gnathifera mean 95% HPD	Gnathifera upper 95% HPD	Gnathostomata lower 95% HPD	Gnathostomata mean 95% HPD	Gnathostomata upper 95% HPD	Hemichordata lower 95% HPD
AC	1	528.3	542.7	549.6	556.0	445.5	458.2	469.4	504.4
	2	528.4	542.8	549.6	556.2	445.5	458.3	469.4	504.4
	3	528.0	543.1	550.1	556.6	445.4	458.1	469.3	504.4
	4	528.5	543.1	550.2	556.7	445.4	458.1	469.4	504.3
	5	528.8	540.5	547.9	554.1	445.5	458.4	469.6	504.4
	6	528.9	540.4	547.9	554.3	445.5	458.4	469.6	504.4
	7	528.4	541.2	548.5	554.8	445.2	458.3	469.4	504.3
	8	528.5	541.3	548.5	555.0	445.4	458.3	469.5	504.4
IR	1	517.6	447.9	516.0	559.7	435.5	453.0	469.0	504.3
	2	517.7	448.8	516.5	559.6	435.5	453.0	469.0	504.3
	3	517.4	451.5	518.0	562.1	435.5	453.0	469.0	504.3
	4	517.6	450.9	517.9	562.8	435.7	453.0	469.1	504.3
	5	517.6	447.1	514.4	557.9	435.5	453.0	468.9	504.3
	6	517.7	447.5	514.4	557.8	435.8	453.0	469.3	504.3
	7	517.4	447.2	515.6	558.8	435.7	453.0	469.1	504.3
	8	517.5	447.1	515.5	558.6	435.7	453.0	469.1	504.3

Model	tree	Hemichordata mean 95% HPD	Hemichordata upper 95% HPD	Lobopoda lower 95% HPD	Lobopoda mean 95% HPD	Lobopoda upper 95% HPD	Mammalia lower 95% HPD	Mammalia mean 95% HPD	Mammalia upper 95% HPD	Mandibulata lower 95% HPD
AC	1	515.4	530.0	538.6	543.5	548.4	180.0	227.3	257.1	526.6
	2	515.5	530.0	538.7	543.5	548.4	181.0	227.4	257.1	526.6
	3	514.3	527.9	538.9	543.9	548.9	180.8	227.6	257.1	526.8
	4	514.3	527.7	538.9	543.9	548.9	181.2	227.6	257.3	526.7
	5	518.5	535.8	538.1	542.8	547.7	179.9	227.0	257.0	526.3
	6	518.5	536.1	537.9	542.8	547.6	180.2	226.8	257.3	526.3
	7	516.7	532.7	538.2	543.1	547.9	180.2	227.2	257.1	526.4
	8	516.7	532.8	538.3	543.2	547.9	180.2	227.1	256.9	526.4
IR	1	515.1	532.3	533.2	543.8	554.4	164.1	205.4	248.8	520.7
	2	515.2	532.7	533.1	543.8	554.5	164.1	205.7	248.9	520.6
	3	513.7	528.9	533.9	545.0	556.2	164.1	205.5	248.9	520.9
	4	513.8	529.1	534.0	544.9	556.3	164.1	205.5	248.7	520.9
	5	517.2	537.3	532.4	542.6	552.9	164.1	205.4	248.8	520.4
	6	517.2	537.7	532.4	542.5	552.9	164.1	205.6	249.0	520.3
	7	515.2	533.0	533.3	543.6	554.4	164.1	205.5	248.8	520.5
	8	515.3	533.3	532.8	543.6	554.1	164.1	205.5	249.0	520.5

Model	tree	Mandibulata mean 95% HPD	Mandibulata upper 95% HPD	Metazoa lower 95% HPD	Metazoa mean 95% HPD	Metazoa upper 95% HPD	Mollusca lower 95% HPD	Mollusca mean 95% HPD	Mollusca upper 95% HPD	Nematoda-Arthropoda lower
AC	1	529.8	532.2	590.2	594.3	598.6	537.5	541.1	544.9	546.3
	2	529.8	532.3	596.6	602.4	609.1	537.7	541.2	545.0	546.4
	3	529.9	532.3	590.2	594.2	598.6	537.8	541.4	545.3	547.0
	4	529.9	532.3	596.6	602.4	609.2	537.8	541.5	545.4	547.2
	5	529.6	532.1	590.3	594.3	598.6	537.1	540.5	544.1	545.2
	6	529.6	532.2	596.5	602.3	609.1	537.1	540.5	543.9	545.1
	7	529.7	532.1	590.3	594.4	598.7	537.4	540.7	544.4	545.7
	8	529.7	532.2	596.5	602.3	609.1	537.4	540.7	544.4	545.7
IR	1	526.8	531.8	594.8	603.3	610.5	537.1	543.2	549.2	541.7
	2	526.7	531.9	597.1	605.1	611.0	537.0	543.2	549.2	541.8
	3	527.0	531.9	594.8	603.4	610.3	537.4	543.6	549.3	543.2
	4	527.0	531.9	597.1	605.1	610.9	537.5	543.6	549.4	542.9
	5	526.5	531.8	594.7	603.3	610.3	536.8	542.7	549.2	540.3
	6	526.5	531.8	597.1	605.0	610.9	536.8	542.7	549.1	540.2
	7	526.7	531.8	594.9	603.4	610.4	537.0	543.1	549.2	541.5
	8	526.7	531.8	597.1	605.1	610.9	537.0	543.1	549.2	541.4

Model	tree	Nematoda-Arthropoda mean	Nematoda-Arthropoda upper	Nephrozoa lower 95% HPD	Nephrozoa mean 95% HPD	Nephrozoa upper 95% HPD	Olfactores lower 95% HPD	Olfactores mean 95% HPD	Olfactores upper 95% HPD	Osteichthyes lower 95% HPD
AC	1	551.3	556.3	564.4	569.3	574.2	539.8	548.7	557.0	420.7
	2	551.3	556.3	564.6	569.5	574.3	540.2	548.8	557.2	420.7
	3	552.0	557.1	NA	NA	NA	540.3	549.1	557.5	420.7
	4	552.1	557.3	NA	NA	NA	540.4	549.2	557.7	420.6
	5	549.9	554.8	562.4	567.3	572.3	543.1	551.2	559.4	420.7
	6	550.0	554.8	562.4	567.5	572.3	543.1	551.4	559.4	420.7
	7	550.5	555.4	563.5	568.4	573.2	543.5	551.6	560.1	420.6
	8	550.5	555.4	563.6	568.5	573.2	543.3	551.7	559.8	420.6
IR	1	552.8	563.5	568.7	575.4	581.8	517.3	533.1	549.2	420.7
	2	552.7	563.8	568.8	575.4	581.9	517.3	533.1	549.0	420.7
	3	554.6	565.8	NA	NA	NA	517.3	534.7	551.6	420.7
	4	554.5	565.8	NA	NA	NA	517.3	534.8	551.8	420.7
	5	551.0	561.3	565.5	572.3	579.1	517.4	535.7	553.0	420.7
	6	550.9	561.2	565.0	572.2	578.9	517.3	535.5	552.7	420.7
	7	552.5	563.1	568.0	575.0	581.4	517.4	536.8	554.7	420.7
	8	552.4	563.3	568.1	574.9	581.6	517.3	536.8	554.6	420.7

Model	tree	Osteichthyes mean 95% HPD	Osteichthyes upper 95% HPD	Pancrustacea lower 95% HPD	Pancrustacea mean 95% HPD	Pancrustacea upper 95% HPD	Placozoa-other-Metazoa [illegible]	Placozoa-other-Metazoa [illegible]	Placozoa-other-Metazoa [illegible]
AC	1	428.2	440.0	514.0	515.9	518.8	584.1	587.5	591.1
	2	428.2	440.1	514.0	515.9	518.9	584.4	587.7	591.1
	3	428.1	439.9	514.0	515.8	518.7	583.8	587.3	590.9
	4	428.1	440.0	514.0	515.8	518.7	584.1	587.5	591.0
	5	428.3	440.2	514.0	516.0	519.0	584.3	587.6	591.2
	6	428.3	440.2	514.0	516.0	519.0	584.6	587.8	591.2
	7	428.2	440.1	514.0	515.9	518.9	584.1	587.5	591.1
	8	428.2	440.1	514.0	515.9	518.9	584.3	587.6	591.1
IR	1	430.2	442.6	514.0	517.6	523.1	583.7	587.8	591.4
	2	430.2	442.6	514.0	517.7	523.2	583.6	587.7	591.4
	3	430.1	442.5	514.0	517.7	523.2	583.4	587.6	591.4
	4	430.2	442.6	514.0	517.7	523.2	583.4	587.5	591.3
	5	430.3	442.6	514.0	517.6	523.1	583.8	587.9	591.5
	6	430.3	442.7	514.0	517.6	523.0	583.7	587.8	591.4
	7	430.2	442.7	514.0	517.6	523.1	583.5	587.7	591.3
	8	430.3	442.7	514.0	517.6	523.0	583.4	587.6	591.3

Model	tree	Porifera lower 95% HPD	Porifera mean 95% HPD	Porifera upper 95% HPD	Porifera-other-Metazoa [illegible]	Porifera-other-Metazoa [illegible]	Porifera-other-Metazoa [illegible]	Protostomia lower 95% HPD	Protostomia mean 95% HPD	Protostomia upper 95% HPD
AC	1	515.0	542.4	566.8	NA	NA	NA	556.9	561.7	566.7
	2	514.9	541.4	565.0	587.8	591.1	594.5	557.0	561.8	566.7
	3	515.0	541.4	565.7	NA	NA	NA	558.1	563.0	567.8
	4	515.0	540.6	563.7	587.8	591.1	594.6	558.2	563.2	568.0
	5	515.0	543.2	567.7	NA	NA	NA	554.9	559.8	564.6
	6	515.0	542.5	566.3	588.0	591.2	594.6	554.9	559.9	564.6
	7	515.0	542.5	566.7	NA	NA	NA	555.9	560.6	565.6
	8	515.0	541.6	564.9	587.9	591.2	594.7	555.9	560.7	565.5
IR	1	514.9	538.1	571.5	NA	NA	NA	562.1	569.6	576.9
	2	514.8	534.3	562.0	587.8	591.1	594.6	562.1	569.6	577.0
	3	514.9	537.7	570.5	NA	NA	NA	564.9	572.4	579.7
	4	514.9	534.0	561.8	587.7	591.1	594.5	564.9	572.4	579.7
	5	514.8	538.3	571.8	NA	NA	NA	559.3	566.9	574.1
	6	514.9	534.5	563.0	587.7	591.1	594.5	559.1	566.8	574.1
	7	514.9	538.0	571.4	NA	NA	NA	561.7	569.2	576.7
	8	514.9	534.1	562.1	587.8	591.1	594.6	561.5	569.1	576.6

Model	tree	Pycnogonida-other-Chelicerates lower 95% HPD	Pycnogonida-other-Chelicerates mean 95% HPD	Pycnogonida-other-Chelicerates upper 95% HPD	Rotifera lower 95% HPD	Rotifera mean 95% HPD	Rotifera upper 95% HPD	Spiralia lower 95% HPD	Spiralia mean 95% HPD	Spiralia upper 95% HPD
AC	1	513.8	521.7	528.4	434.2	503.0	539.1	553.4	558.1	563.0
	2	513.6	521.7	528.3	434.3	503.1	538.4	553.4	558.2	563.0
	3	513.4	521.4	528.2	432.0	502.2	538.4	554.4	559.1	564.0
	4	513.3	521.3	528.1	433.0	502.2	538.2	554.4	559.2	564.0
	5	514.2	521.9	528.6	437.2	504.0	538.4	551.5	556.2	560.9
	6	513.9	521.9	528.3	430.9	503.2	537.9	551.6	556.2	561.0
	7	514.1	521.9	528.6	434.9	503.7	538.3	552.2	556.9	561.6
	8	514.0	521.9	528.5	439.4	504.4	538.3	552.2	556.9	561.6
IR	1	508.9	517.5	528.9	220.0	383.5	523.2	554.2	562.7	571.5
	2	508.9	517.6	529.0	215.7	384.4	522.0	553.8	562.7	571.3
	3	508.9	517.6	529.2	227.5	387.1	522.2	555.9	565.0	573.8
	4	508.9	517.7	529.3	226.5	388.0	522.1	555.8	564.9	573.8
	5	508.9	517.3	528.2	219.4	383.6	521.0	552.1	560.4	568.9
	6	508.8	517.3	528.5	223.1	384.8	520.8	552.0	560.3	569.0
	7	508.8	517.5	528.7	223.4	384.0	524.3	553.4	562.3	570.8
	8	508.9	517.5	528.8	223.6	385.0	522.6	553.4	562.2	570.9

Model	tree	Tetrapoda lower 95% HPD	Tetrapoda mean 95% HPD	Tetrapoda upper 95% HPD	Vertebrata lower 95% HPD	Vertebrata mean 95% HPD	Vertebrata upper 95% HPD	Xenacoelomorpha lower 95% HPD	Xenacoelomorpha mean 95% HPD
AC	1	343.9	348.9	352.1	496.8	506.5	519.0	550.8	560.9
	2	343.9	348.9	352.1	496.8	506.6	519.1	550.9	561.1
	3	344.1	348.9	352.1	496.9	506.4	518.7	529.3	545.6
	4	344.1	348.9	352.1	496.9	506.4	518.9	528.8	545.7
	5	343.7	348.8	352.0	496.9	508.0	521.7	551.3	561.4
	6	343.9	348.8	352.0	496.9	508.0	521.6	551.6	561.8
	7	344.0	348.9	352.1	496.9	507.9	521.4	539.1	552.9
	8	343.9	348.9	352.1	496.9	507.9	521.5	540.0	552.9
IR	1	337.7	344.9	351.2	496.8	506.0	520.8	378.4	500.3
	2	337.8	344.9	351.3	496.8	506.0	520.7	377.0	500.5
	3	337.7	344.9	351.2	496.8	506.3	521.7	348.7	480.7
	4	337.7	344.9	351.2	496.8	506.4	521.7	346.7	480.3
	5	337.7	344.9	351.2	496.8	506.8	522.9	375.2	500.7
	6	337.7	344.9	351.2	496.8	506.9	523.2	377.5	500.9
	7	337.7	344.9	351.2	496.8	507.0	523.7	356.2	488.5
	8	337.7	344.9	351.2	496.8	507.1	523.6	356.1	488.2

Model	tree	Xenacoelomorpha upper 95% HPD	Xenambulacraria lower 95%HPD	Xenambulacraria mean 95%HPD	Xenambulacraria upper 95%HPD
AC	1	570.3	NA	NA	NA
	2	570.4	NA	NA	NA
	3	558.7	556.1	561.8	567.3
	4	558.7	556.3	562.0	567.6
	5	571.4	NA	NA	NA
	6	571.8	NA	NA	NA
	7	564.7	562.0	567.2	572.4
	8	565.1	562.3	567.4	572.6
IR	1	573.7	NA	NA	NA
	2	573.7	NA	NA	NA
	3	554.6	544.7	559.1	572.0
	4	555.0	544.7	559.0	572.3
	5	574.2	NA	NA	NA
	6	574.8	NA	NA	NA
	7	564.4	552.0	566.5	578.9
	8	564.2	551.9	566.4	578.8

Supplementary Table S1: Age estimates from all of the hypotheses tested under the uniform calibration scheme. Lower, upper and mean 95% HPD intervals are provided for all named nodes in all trees.

Model	tree	Acari-Arenacea lower 95% HPD	Acari-Arenacea mean 95% HPD	Acari-Arenacea upper 95% HPD	Ambulacraria lower 95% HPD	Ambulacraria mean 95% HPD	Ambulacraria upper 95% HPD	Amniota lower 95% HPD	Amniota mean 95% HPD
AC	1	437.0	475.3	505.8	542.6	550.3	557.6	317.8	320.3
	2	437.6	475.5	506.2	542.7	550.0	557.5	317.8	320.4
	3	437.1	474.5	505.3	541.1	548.7	556.2	317.8	320.3
	4	437.5	474.7	505.7	540.9	548.4	555.9	317.8	320.3
	5	438.0	476.4	507.4	547.7	556.1	564.0	317.8	320.4
	6	438.0	476.7	507.5	547.7	555.8	563.9	317.8	320.4
	7	438.2	476.0	507.3	546.1	554.4	562.3	317.8	320.4
	8	438.2	476.2	507.4	546.0	554.2	562.0	317.8	320.4
IR	1	434.0	459.3	491.3	528.0	543.4	558.6	317.8	322.4
	2	434.2	459.4	491.8	528.0	543.3	558.7	317.8	322.5
	3	434.0	459.2	491.3	524.3	539.1	553.5	317.8	322.4
	4	434.0	459.3	491.3	524.3	538.9	553.7	317.9	322.4
	5	434.0	459.5	491.8	532.3	549.7	567.9	317.8	322.5
	6	433.9	459.6	491.6	531.9	549.6	567.1	317.9	322.5
	7	434.1	459.2	491.2	527.6	544.2	560.9	317.8	322.4
	8	434.1	459.4	491.2	527.3	543.9	560.6	317.8	322.5

Model	tree	Amniota upper 95% HPD	Annelida lower 95% HPD	Annelida mean 95% HPD	Annelida upper 95% HPD	Annelida-Mollusca lower 95% HPD	Annelida-Mollusca mean 95% HPD	Annelida-Mollusca upper 95% HPD	Bilateria lower 95% HPD	Bilateria mean 95% HPD
AC	1	324.6	518.9	531.9	541.3	545.0	549.6	554.4	565.1	572.3
	2	324.6	519.1	532.0	541.4	544.9	549.4	554.0	564.8	571.8
	3	324.6	518.8	532.0	541.7	545.7	550.3	555.1	563.2	570.0
	4	324.6	519.3	532.1	541.9	545.4	550.1	554.8	562.9	569.6
	5	324.7	519.7	531.9	540.7	543.9	548.3	552.8	566.0	573.0
	6	324.7	519.8	531.9	540.5	543.8	548.1	552.6	565.8	572.6
	7	324.6	519.6	531.9	541.1	544.4	548.9	553.5	564.0	570.8
	8	324.6	519.6	531.9	540.9	544.2	548.7	553.3	563.5	570.4
IR	1	329.2	513.7	525.9	540.5	541.8	550.1	558.7	567.9	577.0
	2	329.2	513.6	526.0	540.4	541.7	549.9	558.5	567.6	576.7
	3	329.1	513.5	526.2	541.3	542.7	551.5	560.8	567.3	576.2
	4	329.1	513.5	526.3	541.5	542.7	551.4	560.7	566.7	575.9
	5	329.2	513.7	525.4	539.4	540.6	548.6	556.3	568.4	577.3
	6	329.2	513.6	525.5	539.3	540.8	548.5	556.4	568.3	577.1
	7	329.1	513.7	525.7	540.2	541.7	549.8	558.3	567.4	576.6
	8	329.2	513.6	525.7	540.1	541.6	549.7	558.2	567.4	576.3

Model	tree	Bilateria upper 95% HPD	Bivalve-Gastropoda lower 95% HPD	Bivalve-Gastropoda mean 95% HPD	Bivalve-Gastropoda upper 95% HPD	Capitellid-Polychaete-Leech lower 95% HPD	Capitellid-Polychaete-Leech mean 95% HPD	Capitellid-Polychaete-Leech upper 95% HPD	Capsospora-Choanozoa lower 95% HPD
AC	1	579.3	531.7	533.0	535.0	507.7	525.2	538.3	624.5
	2	578.7	531.7	533.0	535.0	507.5	525.3	538.0	623.5
	3	577.0	531.7	533.0	535.1	507.0	525.1	538.3	625.1
	4	576.5	531.7	533.0	535.0	507.4	525.2	538.4	623.7
	5	580.3	531.7	532.9	534.7	509.0	525.6	537.7	625.0
	6	579.9	531.7	532.9	534.7	509.2	525.6	537.5	624.7
	7	577.8	531.7	532.9	534.8	508.4	525.4	537.7	625.6
	8	577.3	531.7	532.9	534.8	508.7	525.5	537.9	624.1
IR	1	586.1	531.8	533.9	537.5	428.0	494.0	534.5	672.5
	2	585.7	531.7	533.9	537.4	427.0	493.8	534.9	665.5
	3	585.5	531.8	534.0	537.8	429.6	494.6	536.3	669.9
	4	585.0	531.8	534.0	537.8	429.9	494.7	536.5	667.3
	5	586.5	531.8	533.8	537.2	425.0	492.1	534.2	668.4
	6	586.4	531.8	533.8	537.2	426.4	492.9	534.5	662.8
	7	585.6	531.8	533.9	537.4	428.8	493.6	534.8	671.4
	8	585.5	531.8	533.9	537.4	428.0	493.3	535.8	666.5

Model	tree	Capsospora-Choanozoa mean 95% HPD	Capsospora-Choanozoa upper 95% HPD	Chaetognatha lower 95% HPD	Chaetognatha mean 95% HPD	Chaetognatha upper 95% HPD	Choanoflagellata-Metazoa lower 95% HPD	Choanoflagellata-Metazoa mean 95% HPD
AC	1	680.7	749.1	97.0	344.9	506.2	607.2	636.6
	2	675.9	741.5	104.4	351.3	512.6	606.0	633.5
	3	680.5	748.5	100.4	342.8	504.9	607.0	636.8
	4	675.7	740.5	93.9	344.2	508.6	606.6	633.6
	5	680.2	749.3	100.8	351.3	510.2	607.4	636.7
	6	675.7	742.4	109.5	356.9	509.6	607.0	633.7
	7	680.8	749.1	105.2	349.8	511.1	606.7	637.2
	8	675.8	740.6	102.6	350.4	510.6	606.6	633.8
IR	1	785.3	918.2	50.1	218.8	427.2	621.0	697.0
	2	779.4	906.6	54.8	223.5	435.4	620.4	691.9
	3	786.2	915.2	50.9	216.8	423.9	622.1	698.0
	4	780.5	908.2	49.4	223.1	431.6	619.7	692.6
	5	783.9	913.2	53.2	220.4	435.2	622.8	696.3
	6	778.4	904.0	51.9	220.4	432.4	619.9	691.3
	7	785.6	915.7	52.7	219.9	428.9	621.0	697.4
	8	781.0	909.1	49.4	221.4	430.9	619.2	692.9

Model	tree	Choanoflagellata-Metazoa upper 95% HPD	Chordata lower 95% HPD	Chordata mean 95% HPD	Chordata upper 95% HPD	Cnidaria lower 95% HPD	Cnidaria mean 95% HPD	Cnidaria upper 95% HPD	Copepoda-Branchiopoda lower
AC	1	673.9	550.5	557.5	564.1	561.2	569.7	577.5	496.9
	2	664.5	550.5	557.2	563.9	561.3	569.5	577.2	496.9
	3	673.0	551.3	558.3	565.1	561.2	569.5	577.2	496.8
	4	664.6	551.1	558.0	564.8	561.1	569.3	576.8	496.9
	5	673.0	553.5	559.9	566.5	561.4	570.4	578.6	496.9
	6	665.3	553.2	559.6	566.2	561.3	570.2	578.1	496.9
	7	673.4	554.2	560.7	567.4	561.4	570.3	578.2	496.9
	8	664.9	554.0	560.4	567.1	561.5	570.1	577.9	496.9
IR	1	788.5	529.7	545.5	560.8	560.9	566.6	574.9	496.7
	2	780.2	529.5	545.3	560.5	560.8	566.6	574.9	496.8
	3	790.6	531.3	547.9	564.3	560.8	566.5	574.8	496.7
	4	781.0	530.6	547.7	563.6	560.9	566.4	574.6	496.7
	5	789.4	531.6	549.2	564.6	560.9	566.7	575.1	496.8
	6	780.1	531.9	549.1	565.1	560.9	566.7	575.1	496.7
	7	789.5	532.7	551.2	567.8	560.8	566.6	574.9	496.7
	8	780.8	533.1	550.9	567.9	560.8	566.5	574.8	496.7

Model	tree	Copepoda-Branchiopoda mean	Copepoda-Branchiopoda upper	Ctenophora lower 95% HPD	Ctenophora mean 95% HPD	Ctenophora upper 95% HPD	Ctenophora-other-Metazoa lower	Ctenophora-other-Metazoa mean
AC	1	501.8	507.3	161.1	402.4	534.5	584.2	594.0
	2	501.9	507.3	243.4	433.0	549.8	NA	NA
	3	501.7	507.1	162.3	397.8	534.8	584.1	593.9
	4	501.8	507.2	213.8	427.4	552.8	NA	NA
	5	502.0	507.4	187.2	411.8	538.8	584.8	594.7
	6	502.0	507.5	209.2	432.6	553.1	NA	NA
	7	501.9	507.3	176.7	407.9	536.7	584.7	594.6
	8	501.9	507.4	219.7	431.7	552.0	NA	NA
IR	1	502.1	509.3	130.1	336.0	537.6	585.4	596.7
	2	502.2	509.5	129.3	353.6	549.0	NA	NA
	3	502.1	509.3	124.7	330.4	531.4	585.4	596.7
	4	502.1	509.4	127.2	349.8	544.8	NA	NA
	5	502.1	509.5	125.2	337.3	539.7	585.2	596.7
	6	502.2	509.4	128.8	348.2	545.4	NA	NA
	7	502.1	509.2	126.1	329.3	533.5	585.5	596.8
	8	502.1	509.4	126.9	349.6	545.4	NA	NA

Model	tree	Ctenophora-other-Metazoa upper 95%	Cyclostomata lower 95% HPD	Cyclostomata mean 95% HPD	Cyclostomata upper 95% HPD	Deuterostomia lower 95% HPD	Deuterostomia mean 95% HPD	Deuterostomia upper 95% HPD
AC	1	604.0	373.3	443.8	492.0	557.4	563.8	570.3
	2	NA	373.1	444.3	492.5	557.2	563.5	569.9
	3	604.0	373.6	443.6	491.9	558.6	565.1	571.8
	4	NA	373.3	444.3	491.8	558.3	564.7	571.4
	5	604.6	372.3	443.5	493.8	NA	NA	NA
	6	NA	372.2	444.1	494.3	NA	NA	NA
	7	604.7	372.1	443.4	493.2	NA	NA	NA
	8	NA	372.2	443.8	493.6	NA	NA	NA
IR	1	608.1	357.7	410.4	472.0	549.2	561.5	573.3
	2	NA	358.0	410.5	472.5	549.1	561.2	573.0
	3	608.2	357.6	410.4	471.8	553.1	565.4	577.1
	4	NA	357.2	410.2	471.0	552.8	565.1	577.0
	5	607.8	357.7	410.6	472.5	NA	NA	NA
	6	NA	357.6	410.9	473.1	NA	NA	NA
	7	608.2	357.6	410.7	472.4	NA	NA	NA
	8	NA	357.8	410.9	472.7	NA	NA	NA

Model	tree	Ecdysozoa lower 95% HPD	Ecdysozoa mean 95% HPD	Ecdysozoa upper 95% HPD	Echinodermata lower 95% HPD	Echinodermata mean 95% HPD	Echinodermata upper 95% HPD	Euarchontoglires lower 95% HPD
AC	1	549.4	555.0	560.7	508.6	516.1	526.5	65.1
	2	549.3	554.7	560.4	508.7	516.1	526.5	65.0
	3	550.5	556.2	562.0	508.6	515.5	525.4	65.2
	4	550.2	555.9	561.7	508.6	515.5	525.2	65.1
	5	548.2	553.6	559.0	508.6	517.8	529.9	65.1
	6	548.0	553.3	558.7	508.6	517.9	529.9	64.8
	7	548.9	554.3	560.0	508.7	517.1	528.7	65.2
	8	548.5	554.0	559.5	508.7	517.2	528.6	65.0
IR	1	545.7	556.1	566.3	508.5	516.2	527.8	64.6
	2	545.3	555.9	566.0	508.5	516.3	528.1	64.7
	3	547.4	558.4	569.3	508.5	515.3	525.8	64.5
	4	547.0	558.2	569.0	508.6	515.3	525.9	64.6
	5	543.6	553.9	563.3	508.6	517.4	530.9	64.8
	6	544.1	553.7	563.5	508.5	517.5	531.1	64.6
	7	545.4	555.8	566.0	508.5	516.4	528.4	64.8
	8	545.4	555.6	565.9	508.5	516.4	528.5	64.7

Model	tree	Euarchontoglires mean 95% HPD	Euarchontoglires upper 95% HPD	Euarthropoda lower 95% HPD	Euarthropoda mean 95% HPD	Euarthropoda upper 95% HPD	Eumetabola lower 95% HPD	Eumetabola mean 95% HPD
AC	1	92.9	130.9	528.4	533.5	538.7	324.5	375.0
	2	92.9	130.6	528.4	533.4	538.5	323.5	374.9
	3	92.9	131.3	528.7	533.8	539.1	325.7	375.1
	4	92.9	131.0	528.6	533.7	538.9	324.3	375.3
	5	93.1	131.4	528.1	533.0	537.9	323.8	374.8
	6	93.1	130.8	528.1	532.8	537.8	325.0	374.8
	7	93.0	130.8	528.3	533.3	538.4	324.3	374.9
	8	93.0	131.1	528.2	533.1	538.2	324.9	375.0
IR	1	88.1	127.5	523.1	531.2	539.7	319.4	354.2
	2	88.2	127.5	523.0	531.1	539.8	319.6	354.3
	3	87.8	126.5	523.4	531.8	540.7	319.5	354.3
	4	87.9	126.6	523.3	531.7	540.7	319.7	354.4
	5	88.3	127.6	522.8	530.5	538.7	319.7	354.3
	6	88.4	127.9	522.8	530.5	538.8	319.6	354.4
	7	88.1	127.1	523.2	531.1	539.8	319.6	354.3
	8	88.1	127.3	523.0	531.0	539.5	319.6	354.4

Model	tree	Eumetabola upper 95% HPD	Eumetazoa lower 95% HPD	Eumetazoa mean 95% HPD	Eumetazoa upper 95% HPD	Fungi-Holozoa lower 95% HPD	Fungi-Holozoa mean 95% HPD	Fungi-Holozoa upper 95% HPD	Gastropoda lower 95% HPD	Gastropoda mean 95% HPD
AC	1	414.1	576.2	584.5	592.7	642.3	728.4	837.9	474.1	502.1
	2	413.4	575.8	583.8	592.0	640.1	724.4	832.8	473.7	502.1
	3	414.1	575.9	584.2	592.4	642.6	727.4	836.3	473.7	501.5
	4	413.6	575.1	583.5	591.4	641.7	722.5	830.9	473.8	501.5
	5	413.9	577.0	585.2	593.6	643.1	727.6	838.2	474.6	503.7
	6	415.4	576.2	584.5	592.6	640.6	723.6	829.8	474.5	503.8
	7	413.7	576.6	584.8	593.2	641.3	728.3	837.1	474.4	503.0
	8	415.0	576.0	584.1	592.3	640.9	723.1	830.5	474.1	503.1
IR	1	394.2	575.1	584.1	593.8	716.2	875.8	1053.9	469.8	483.3
	2	394.6	574.6	583.7	592.9	714.6	871.2	1045.1	469.8	483.4
	3	393.8	574.6	583.8	593.1	718.3	876.5	1050.6	469.8	483.3
	4	394.3	574.0	583.4	592.5	714.1	872.0	1045.4	469.9	483.3
	5	394.1	575.0	584.2	593.5	716.9	874.7	1049.7	469.9	483.4
	6	394.7	574.7	583.9	593.2	712.9	869.6	1043.8	469.8	483.4
	7	394.4	574.7	584.0	593.2	716.8	876.1	1046.1	469.9	483.3
	8	394.2	574.3	583.6	592.7	714.8	872.4	1045.7	469.8	483.3

Model	tree	Gastropoda upper 95% HPD	Gnathifera lower 95% HPD	Gnathifera mean 95% HPD	Gnathifera upper 95% HPD	Gnathostomata lower 95% HPD	Gnathostomata mean 95% HPD	Gnathostomata upper 95% HPD	Hemichordata lower 95% HPD
AC	1	523.8	541.3	548.3	554.9	440.0	453.2	466.8	503.7
	2	523.6	541.1	548.1	554.6	439.9	453.2	466.9	503.8
	3	523.0	541.4	548.7	555.4	440.0	453.0	466.5	503.8
	4	523.2	541.3	548.6	555.3	440.1	453.1	466.5	503.7
	5	524.8	539.7	546.8	553.3	439.7	453.2	467.0	503.9
	6	524.8	539.7	546.7	553.1	440.0	453.2	467.4	503.9
	7	524.3	540.2	547.4	554.0	439.8	453.1	466.9	503.7
	8	524.2	540.1	547.2	553.8	439.9	453.1	467.0	503.7
IR	1	502.3	445.5	513.5	555.4	431.6	446.0	462.6	503.5
	2	502.5	445.8	513.9	554.6	431.6	446.0	462.5	503.5
	3	502.2	446.8	514.8	557.0	431.7	446.0	462.3	503.5
	4	502.3	447.7	515.1	557.7	431.7	446.0	462.4	503.4
	5	502.4	440.5	511.0	553.0	431.7	446.0	462.5	503.6
	6	502.5	441.8	511.5	552.4	431.6	446.0	462.5	503.5
	7	502.2	444.7	512.8	554.8	431.7	446.0	462.6	503.3
	8	502.4	444.0	512.9	554.7	431.6	446.0	462.5	503.5

Model	tree	Hemichordata mean 95% HPD	Hemichordata upper 95% HPD	Lobopoda lower 95% HPD	Lobopoda mean 95% HPD	Lobopoda upper 95% HPD	Mammalia lower 95% HPD	Mammalia mean 95% HPD	Mammalia upper 95% HPD	Mandibulata lower 95% HPD
AC	1	513.6	526.4	537.2	542.8	548.6	164.1	210.6	260.1	525.0
	2	513.7	526.8	537.1	542.7	548.3	164.1	210.4	259.5	525.1
	3	512.7	524.8	537.6	543.4	549.2	164.0	211.2	259.8	525.5
	4	512.7	524.8	537.5	543.2	549.0	164.0	210.8	259.7	525.3
	5	515.8	531.0	536.7	542.1	547.6	164.0	210.2	259.6	524.8
	6	515.9	531.2	536.6	541.9	547.4	164.0	210.0	259.8	524.8
	7	514.4	528.6	537.0	542.5	548.1	164.1	210.6	260.1	525.0
	8	514.5	528.6	536.8	542.3	547.8	164.0	210.2	259.2	525.0
IR	1	513.1	527.4	530.0	539.7	549.0	163.2	191.9	235.5	517.8
	2	513.1	527.7	530.0	539.6	549.0	163.3	192.0	235.2	518.1
	3	511.9	524.5	530.5	540.8	550.8	163.2	191.9	235.1	518.1
	4	512.0	524.8	530.5	540.6	550.7	163.2	192.0	235.7	518.1
	5	514.7	531.7	529.7	538.6	547.3	163.3	192.0	236.2	517.8
	6	514.7	531.5	529.6	538.5	547.3	163.3	192.0	235.2	517.8
	7	513.2	527.8	530.0	539.6	548.9	163.2	191.9	234.9	517.9
	8	513.2	527.8	529.9	539.4	548.7	163.1	191.9	235.0	518.0

Model	tree	Mandibulata mean 95% HPD	Mandibulata upper 95% HPD	Metazoa lower 95% HPD	Metazoa mean 95% HPD	Metazoa upper 95% HPD	Mollusca lower 95% HPD	Mollusca mean 95% HPD	Mollusca upper 95% HPD	Nematoda-Arthropoda lower
AC	1	529.8	534.6	586.8	597.4	608.3	537.3	540.4	543.7	544.8
	2	529.7	534.6	591.5	603.9	616.8	537.2	540.3	543.5	544.5
	3	530.2	535.2	586.6	597.4	608.1	537.5	540.7	544.0	545.5
	4	530.1	535.1	591.4	603.9	616.6	537.4	540.6	543.9	545.1
	5	529.4	534.1	587.3	598.1	608.8	536.9	539.8	543.0	543.6
	6	529.3	534.0	591.9	604.6	617.3	536.8	539.7	542.8	543.3
	7	529.6	534.5	587.0	598.0	608.7	537.1	540.1	543.3	544.3
	8	529.5	534.4	591.9	604.4	617.2	537.0	540.0	543.1	543.9
IR	1	524.2	530.9	592.3	607.3	622.5	535.0	540.5	546.6	537.8
	2	524.2	531.0	595.2	611.2	628.2	534.9	540.5	546.5	537.4
	3	524.6	531.3	592.8	607.4	623.3	535.1	541.0	547.3	538.6
	4	524.5	531.3	595.0	611.3	628.3	535.1	541.0	547.3	538.7
	5	523.9	530.3	592.7	607.3	622.9	534.8	540.0	545.9	536.2
	6	523.8	530.4	594.9	611.1	628.0	534.7	540.0	545.6	536.5
	7	524.2	530.8	592.5	607.5	623.0	535.0	540.4	546.5	537.8
	8	524.1	530.9	595.2	611.2	628.5	534.9	540.4	546.4	537.5

Model	tree	Nematoda-Arthropoda mean 95% HPD	Nematoda-Arthropoda upper 95% HPD	Nephrozoa lower 95% HPD	Nephrozoa mean 95% HPD	Nephrozoa upper 95% HPD	Olfactores lower 95% HPD	Olfactores mean 95% HPD	Olfactores upper 95% HPD	Osteichthyes lower 95% HPD
AC	1	550.2	556.0	561.9	568.4	575.3	537.2	546.3	554.8	420.4
	2	550.0	555.5	561.5	568.0	574.7	537.0	546.2	554.6	420.4
	3	551.1	557.0	NA	NA	NA	537.4	546.7	555.5	420.4
	4	550.9	556.5	NA	NA	NA	537.2	546.5	555.0	420.4
	5	548.9	554.3	559.9	566.4	573.0	540.7	549.1	557.7	420.4
	6	548.7	554.0	559.7	566.0	572.5	540.8	549.0	557.7	420.4
	7	549.5	555.2	561.1	567.6	574.3	540.8	549.5	558.1	420.4
	8	549.3	554.7	560.7	567.1	573.8	540.9	549.4	558.0	420.4
IR	1	548.1	558.5	563.4	572.2	581.4	517.0	529.2	543.4	420.4
	2	548.0	558.3	563.2	571.9	580.8	517.1	529.2	543.3	420.4
	3	549.8	560.8	NA	NA	NA	517.2	530.4	545.6	420.4
	4	549.6	560.8	NA	NA	NA	517.0	530.3	545.3	420.5
	5	546.4	556.0	559.6	568.4	577.0	517.3	531.3	546.6	420.5
	6	546.3	556.1	559.7	568.2	577.0	517.2	531.2	546.7	420.4
	7	547.9	558.3	562.9	571.7	580.8	517.3	532.2	548.4	420.4
	8	547.7	558.2	562.5	571.3	580.5	517.2	532.1	548.3	420.5

Model	tree	Osteichthyes mean 95% HPD	Osteichthyes upper 95% HPD	Pancrustacea lower 95% HPD	Pancrustacea mean 95% HPD	Pancrustacea upper 95% HPD	Placozoa-other-Metazoa [illegible]	Placozoa-other-Metazoa [illegible]	Placozoa-other-Metazoa [illegible]
AC	1	425.3	433.5	513.8	515.8	519.1	580.3	589.4	598.3
	2	425.4	433.7	513.8	515.8	519.1	579.4	588.3	597.0
	3	425.3	433.3	513.8	515.8	519.1	580.0	589.1	598.0
	4	425.3	433.5	513.8	515.8	519.1	579.1	588.1	596.7
	5	425.4	433.6	513.8	515.8	519.0	580.9	589.9	599.1
	6	425.4	433.5	513.8	515.8	518.9	580.1	589.0	597.7
	7	425.3	433.5	513.8	515.8	519.0	580.7	589.7	598.8
	8	425.4	433.5	513.8	515.8	519.0	580.0	588.7	597.6
IR	1	426.6	436.0	513.8	516.4	520.5	579.6	589.6	599.7
	2	426.7	436.0	513.8	516.4	520.6	579.4	589.1	599.1
	3	426.6	435.9	513.8	516.4	520.7	579.4	589.4	599.4
	4	426.7	435.9	513.8	516.4	520.7	579.2	588.9	599.2
	5	426.7	436.3	513.8	516.3	520.4	580.1	589.7	599.9
	6	426.7	436.2	513.8	516.3	520.4	579.5	589.3	599.2
	7	426.7	435.9	513.8	516.3	520.4	579.5	589.6	599.5
	8	426.7	436.1	513.8	516.4	520.4	579.1	589.1	599.0

Model	tree	Porifera lower 95% HPD	Porifera mean 95% HPD	Porifera upper 95% HPD	Porifera-other-Metazoa [illegible]	Porifera-other-Metazoa [illegible]	Porifera-other-Metazoa [illegible]	Protostomia lower 95% HPD	Protostomia mean 95% HPD	Protostomia upper 95% HPD
AC	1	514.7	536.3	561.9	NA	NA	NA	554.6	560.5	566.5
	2	514.8	535.9	560.6	582.8	592.3	601.9	554.5	560.2	566.1
	3	514.6	536.0	560.5	NA	NA	NA	555.9	562.0	568.0
	4	514.7	535.5	558.8	582.8	592.3	601.8	555.6	561.6	567.6
	5	514.8	537.0	563.3	NA	NA	NA	553.0	558.6	564.4
	6	514.8	536.6	561.7	583.6	593.1	602.7	552.7	558.4	564.0
	7	514.7	536.7	562.0	NA	NA	NA	553.8	559.6	565.4
	8	514.7	536.3	560.1	583.6	593.0	602.7	553.6	559.2	565.1
IR	1	514.1	533.1	561.1	NA	NA	NA	556.8	565.5	574.4
	2	514.3	531.5	557.3	584.9	595.8	607.1	556.6	565.3	574.0
	3	514.2	532.8	560.7	NA	NA	NA	559.5	568.7	577.9
	4	514.1	531.2	556.3	584.9	595.8	607.4	559.3	568.4	577.6
	5	514.3	533.3	562.0	NA	NA	NA	554.3	562.5	571.1
	6	514.3	531.5	557.5	584.6	596.0	607.1	553.9	562.3	570.7
	7	514.2	533.0	561.2	NA	NA	NA	556.5	565.1	574.0
	8	514.2	531.3	556.8	584.8	596.0	607.4	556.1	564.8	573.7

Model	tree	Pycnogonida-other-Chelicerates lower 95% [illegible]	Pycnogonida-other-Chelicerates mean 95% [illegible]	Pycnogonida-other-Chelicerates upper 95% [illegible]	Rotifera lower 95% HPD	Rotifera mean 95% HPD	Rotifera upper 95% HPD	Spiralia lower 95% HPD	Spiralia mean 95% HPD	Spiralia upper 95% HPD
AC	1	512.8	521.7	529.5	434.8	501.8	537.0	551.3	556.9	562.4
	2	512.9	521.6	529.3	435.3	501.8	538.1	551.3	556.6	562.2
	3	512.8	521.6	529.7	428.2	499.7	536.7	552.4	558.0	563.7
	4	512.7	521.6	529.6	432.9	500.4	537.7	552.1	557.7	563.3
	5	513.1	521.6	529.3	436.5	502.7	537.6	549.8	555.0	560.4
	6	513.2	521.6	529.5	436.7	502.9	536.8	549.6	554.8	560.1
	7	513.3	521.8	529.6	438.5	502.7	537.2	550.5	555.8	561.3
	8	513.2	521.7	529.5	434.3	502.2	537.1	550.3	555.5	561.0
IR	1	508.3	515.8	525.7	224.9	383.1	518.3	549.5	558.5	567.6
	2	508.2	515.9	525.7	228.9	383.9	519.0	549.5	558.3	567.5
	3	508.3	516.0	526.1	232.2	384.6	518.5	551.1	560.8	570.4
	4	508.3	516.0	526.1	234.5	385.4	521.3	550.8	560.6	570.0
	5	508.4	515.6	525.2	222.8	380.2	518.8	547.6	556.1	564.7
	6	508.2	515.7	525.2	223.2	382.1	519.6	547.6	555.9	564.4
	7	508.2	515.8	525.6	227.6	382.5	518.7	549.2	558.1	567.2
	8	508.2	515.8	525.5	226.4	383.5	519.7	548.9	557.8	566.8

Model	tree	Tetrapoda lower 95% HPD	Tetrapoda mean 95% HPD	Tetrapoda upper 95% HPD	Vertebrata lower 95% HPD	Vertebrata mean 95% HPD	Vertebrata upper 95% HPD	Xenacoelomorpha lower 95% HPD	Xenacoelomorpha mean 95% HPD
AC	1	340.6	349.7	358.6	496.0	504.2	515.3	547.4	559.0
	2	340.7	349.5	358.7	496.1	504.2	515.4	546.9	558.7
	3	340.8	349.9	358.9	496.0	504.0	515.0	525.1	542.7
	4	340.6	349.7	358.7	496.0	504.0	515.2	525.1	542.5
	5	340.4	349.4	358.5	496.1	505.3	517.5	547.1	559.1
	6	340.1	349.3	358.3	496.2	505.4	517.9	547.1	559.2
	7	340.5	349.6	358.6	496.2	505.3	517.7	533.0	550.2
	8	340.5	349.5	358.4	496.0	505.3	517.5	532.9	550.0
IR	1	336.9	342.3	349.5	495.9	504.2	516.3	383.4	500.1
	2	336.9	342.3	349.5	495.9	504.3	516.8	374.4	499.1
	3	336.9	342.3	349.5	495.9	504.4	517.1	343.1	477.0
	4	336.9	342.3	349.5	495.9	504.4	517.2	342.2	476.6
	5	336.9	342.3	349.5	495.8	504.8	517.9	375.9	500.1
	6	336.9	342.3	349.5	495.8	504.9	518.2	378.5	500.3
	7	336.9	342.3	349.5	495.8	505.0	518.2	352.7	485.6
	8	336.9	342.3	349.5	495.8	505.0	518.4	351.6	485.4

Model	tree	Xenacoelomorpha upper 95% HPD	Xenambulacraria lower 95%HPD	Xenambulacraria mean 95%HPD	Xenambulacraria upper 95%HPD
AC	1	569.8	NA	NA	NA
	2	569.2	NA	NA	NA
	3	557.1	553.6	560.2	566.9
	4	557.2	553.2	559.9	566.6
	5	571.2	NA	NA	NA
	6	571.1	NA	NA	NA
	7	562.8	559.3	566.2	572.9
	8	562.6	559.2	565.9	572.7
IR	1	572.5	NA	NA	NA
	2	572.0	NA	NA	NA
	3	547.3	539.2	553.5	567.8
	4	547.8	538.5	553.3	567.5
	5	573.6	NA	NA	NA
	6	572.7	NA	NA	NA
	7	558.3	545.4	561.6	576.4
	8	558.1	545.3	561.3	576.2

Supplementary Table S2: Age estimates from all of the hypotheses tested under the skew Normal calibration scheme. Lower, upper and mean 95% HPD intervals are provided for all named nodes in all trees.

Model	tree	Acari-Arenacea lower 95% HPD	Acari-Arenacea mean 95% HPD	Acari-Arenacea upper 95% HPD	Ambulacraria lower 95% HPD	Ambulacraria mean 95% HPD	Ambulacraria upper 95% HPD	Amniota lower 95% HPD	Amniota mean 95% HPD
AC	1	460.3	490.9	516.0	548.1	555.2	561.8	312.7	319.7
	2	460.8	491.0	516.3	548.2	555.1	561.9	312.7	319.8
	3	459.4	490.1	515.4	546.6	553.6	560.4	312.6	319.6
	4	459.6	490.3	515.3	546.5	553.5	560.3	312.5	319.7
	5	461.2	491.8	516.1	553.7	560.6	567.3	312.9	319.8
	6	461.4	492.0	516.3	553.6	560.5	567.1	312.8	319.9
	7	461.3	491.4	516.2	552.1	559.1	566.0	312.7	319.7
	8	461.8	491.7	516.1	552.0	559.0	565.8	312.9	319.8
IR	1	437.9	479.2	517.3	539.8	553.4	566.8	316.5	324.0
	2	437.3	479.6	517.5	539.8	553.3	566.6	316.4	324.0
	3	436.7	479.0	517.6	535.6	549.2	562.5	316.5	324.0
	4	436.0	479.2	516.1	535.7	549.2	562.5	316.4	324.0
	5	436.7	479.1	517.0	544.1	558.8	572.9	316.6	324.0
	6	437.6	479.6	517.4	544.0	558.7	572.8	316.5	324.1
	7	437.7	478.9	518.4	539.0	553.6	567.8	316.3	324.0
	8	453.7	487.4	516.9	547.5	557.2	566.4	314.1	321.2

Model	tree	Amniota upper 95% HPD	Annelida lower 95% HPD	Annelida mean 95% HPD	Annelida upper 95% HPD	Annelida-Mollusca lower 95% HPD	Annelida-Mollusca mean 95% HPD	Annelida-Mollusca upper 95% HPD	Bilateria lower 95% HPD	Bilateria mean 95% HPD
AC	1	326.7	524.0	534.7	543.9	546.6	551.8	556.6	569.8	575.0
	2	326.6	524.3	534.8	544.1	546.6	551.6	556.6	569.4	574.6
	3	326.5	524.0	534.8	544.4	547.4	552.4	557.4	567.7	573.0
	4	326.6	523.9	534.9	544.2	547.3	552.3	557.3	567.4	572.6
	5	326.9	524.1	534.2	543.4	545.3	550.2	555.2	570.1	575.5
	6	326.8	524.1	534.3	543.4	545.2	550.1	555.0	570.0	575.1
	7	326.6	523.9	534.3	543.4	545.9	550.8	555.8	568.3	573.5
	8	327.0	524.3	534.4	543.6	545.7	550.7	555.6	568.1	573.2
IR	1	331.6	515.8	535.2	553.1	546.1	555.3	564.2	573.6	579.9
	2	331.4	515.5	535.4	552.9	546.2	555.2	564.2	573.2	579.7
	3	331.5	515.8	535.7	553.6	547.2	556.5	566.0	572.8	579.4
	4	331.4	515.8	536.0	553.6	547.2	556.4	565.9	572.9	579.1
	5	331.7	515.5	534.3	551.7	544.9	553.8	562.2	574.0	580.2
	6	331.5	515.8	534.5	552.0	545.1	553.8	562.3	573.6	580.0
	7	331.3	515.4	534.8	552.3	546.1	554.9	563.9	573.1	579.6
	8	328.4	521.2	534.6	546.4	545.7	552.1	558.3	569.5	575.2

Model	tree	Bilateria upper 95% HPD	Bivalve-Gastropoda lower 95% HPD	Bivalve-Gastropoda mean 95% HPD	Bivalve-Gastropoda upper 95% HPD	Capitellid-Polychaete-Leech lower 95%	Capitellid-Polychaete-Leech mean 95%	Capitellid-Polychaete-Leech upper 95%	Capsospora-Choanozoa lower 95% HPD
AC	1	580.4	523.1	529.5	535.8	514.2	528.1	540.7	656.0
	2	579.8	523.2	529.6	535.8	514.0	528.3	540.5	649.4
	3	578.1	523.3	529.7	536.0	513.7	528.0	541.0	658.0
	4	577.8	523.5	529.7	536.2	514.0	528.2	541.0	649.6
	5	580.6	522.8	529.2	535.4	514.5	528.0	540.1	658.9
	6	580.4	523.0	529.2	535.5	514.3	528.1	540.2	645.1
	7	578.7	522.9	529.3	535.5	514.2	527.9	540.1	660.7
	8	578.4	523.1	529.4	535.7	514.4	528.0	540.1	647.6
IR	1	586.2	525.0	532.8	540.2	438.1	502.7	544.2	844.8
	2	586.0	525.2	532.8	540.3	437.8	503.1	544.0	834.3
	3	585.8	525.2	532.8	540.6	439.8	503.6	545.1	841.7
	4	585.7	525.2	532.9	540.7	439.6	503.3	545.1	829.4
	5	586.6	524.9	532.6	540.2	435.7	500.9	542.5	843.1
	6	586.4	525.1	532.7	540.3	433.8	501.0	542.7	836.5
	7	585.9	525.0	532.7	540.2	438.7	501.9	544.8	845.6
	8	580.8	523.7	530.5	537.0	488.1	519.4	541.5	705.3

Model	tree	Capsospora-Choanozoa mean 95% HPD	Capsospora-Choanozoa upper 95% HPD	Chaetognatha lower 95% HPD	Chaetognatha mean 95% HPD	Chaetognatha upper 95% HPD	Choanoflagellata-Metazoa lower 95% HPD	Choanoflagellata-Metazoa mean 95% HPD
AC	1	959.1	1241.0	113.6	343.6	508.1	610.1	769.6
	2	952.0	1244.9	111.9	348.3	511.8	607.9	752.4
	3	960.3	1241.1	111.0	340.8	506.8	610.9	772.9
	4	948.5	1238.9	107.1	343.5	509.2	609.1	753.7
	5	959.3	1244.7	112.4	345.1	510.8	609.1	769.6
	6	954.5	1243.2	109.6	349.7	511.9	608.0	754.1
	7	958.1	1245.8	105.9	341.9	509.6	610.8	769.1
	8	948.5	1237.3	112.4	348.1	514.7	608.6	753.7
IR	1	1081.2	1315.5	59.2	228.4	440.7	689.7	908.1
	2	1072.9	1317.3	55.7	234.6	452.0	672.6	894.0
	3	1080.5	1321.5	54.4	229.7	440.9	684.4	906.6
	4	1073.1	1307.3	55.7	231.9	443.9	673.0	892.1
	5	1082.9	1322.9	58.4	230.8	443.8	681.9	909.9
	6	1077.2	1318.0	56.6	231.9	446.5	665.7	897.9
	7	1081.8	1319.6	55.4	228.8	437.6	686.3	907.3
	8	988.6	1262.2	91.1	307.7	487.1	629.6	798.8

Model	tree	Choanoflagellata-Metazoa upper 95% HPD	Chordata lower 95% HPD	Chordata mean 95% HPD	Chordata upper 95% HPD	Cnidaria lower 95% HPD	Cnidaria mean 95% HPD	Cnidaria upper 95% HPD	Copepoda-Branchiopoda lower [illegible]
AC	1	1038.1	555.7	561.6	567.2	566.0	573.5	580.5	495.1
	2	1038.4	555.6	561.4	567.1	566.1	573.4	580.2	495.2
	3	1043.5	556.5	562.3	568.0	565.9	573.3	580.2	495.1
	4	1038.6	556.4	562.1	567.9	566.1	573.1	580.0	495.2
	5	1043.6	557.8	563.4	568.9	566.5	574.0	581.0	495.4
	6	1044.5	557.7	563.2	568.6	566.6	573.8	580.7	495.5
	7	1039.1	558.6	564.2	569.8	566.6	573.8	580.8	495.2
	8	1037.6	558.5	564.0	569.4	566.7	573.7	580.5	495.3
IR	1	1139.1	542.6	555.9	568.8	560.3	570.5	580.8	495.3
	2	1127.0	541.8	555.8	568.4	560.0	570.5	580.5	495.0
	3	1132.2	543.7	558.0	570.9	559.8	570.3	580.3	494.7
	4	1117.5	543.9	558.0	571.3	559.8	570.3	580.1	494.9
	5	1138.1	544.9	559.0	571.4	560.1	570.7	580.9	494.6
	6	1124.9	544.9	559.0	571.5	560.2	570.7	580.7	495.1
	7	1138.3	546.5	560.7	574.0	559.9	570.5	580.3	494.8
	8	1064.7	554.2	562.8	570.9	564.5	572.6	580.6	495.1

Model	tree	Copepoda-Branchiopoda mean 95% HPD	Copepoda-Branchiopoda upper 95% HPD	Ctenophora lower 95% HPD	Ctenophora mean 95% HPD	Ctenophora upper 95% HPD	Ctenophora-other-Metazoa lower 95% HPD	Ctenophora-other-Metazoa mean 95% HPD
AC	1	503.9	512.3	190.2	405.0	536.6	588.1	594.6
	2	504.0	512.4	197.8	422.2	545.9	NA	NA
	3	503.8	512.3	175.6	398.2	535.5	588.2	594.7
	4	503.9	512.5	191.5	420.3	543.5	NA	NA
	5	504.1	512.6	184.7	406.5	539.7	588.4	594.9
	6	504.2	512.7	212.8	431.8	553.1	NA	NA
	7	504.0	512.5	173.8	400.8	538.4	588.4	594.9
	8	504.1	512.4	207.3	426.9	547.2	NA	NA
IR	1	505.5	516.1	143.7	339.8	532.0	587.3	594.5
	2	505.6	515.9	145.4	360.3	554.9	NA	NA
	3	505.4	515.6	140.9	341.2	541.2	587.3	594.6
	4	505.5	515.7	143.8	357.8	553.4	NA	NA
	5	505.5	515.5	139.3	345.6	541.0	587.5	594.6
	6	505.6	515.8	146.5	367.9	560.6	NA	NA
	7	505.4	515.6	143.5	342.1	537.4	587.3	594.6
	8	504.6	513.5	200.5	406.9	550.6	NA	NA

Model	tree	Ctenophora-other-Metazoa upper 95%	Cyclostomata lower 95% HPD	Cyclostomata mean 95% HPD	Cyclostomata upper 95% HPD	Deuterostomia lower 95% HPD	Deuterostomia mean 95% HPD	Deuterostomia upper 95% HPD
AC	1	601.1	421.4	467.5	507.4	562.2	567.3	572.6
	2	NA	420.3	467.7	507.8	561.8	567.0	572.2
	3	601.2	420.6	467.1	506.4	563.3	568.5	573.7
	4	NA	421.1	467.5	507.3	562.9	568.2	573.3
	5	601.3	420.7	469.2	510.0	NA	NA	NA
	6	NA	422.0	469.7	511.1	NA	NA	NA
	7	601.3	421.1	468.8	510.0	NA	NA	NA
	8	NA	421.9	469.2	510.4	NA	NA	NA
IR	1	601.8	384.4	447.9	506.2	559.1	568.3	577.2
	2	NA	382.9	448.0	505.8	558.9	568.1	577.0
	3	601.9	383.2	447.9	505.2	562.3	571.4	579.9
	4	NA	382.5	448.2	505.8	562.2	571.2	579.7
	5	602.1	383.7	449.1	507.7	NA	NA	NA
	6	NA	383.9	449.4	507.6	NA	NA	NA
	7	601.8	384.7	448.7	508.4	NA	NA	NA
	8	NA	409.1	462.6	509.3	NA	NA	NA

Model	tree	Ecdysozoa lower 95% HPD	Ecdysozoa mean 95% HPD	Ecdysozoa upper 95% HPD	Echinodermata lower 95% HPD	Echinodermata mean HPD	Echinodermata upper 95% HPD	Euarchontoglires lower 95% HPD
AC	1	553.3	558.3	563.4	510.4	524.1	537.6	75.3
	2	553.1	558.1	563.2	510.6	524.2	537.5	76.5
	3	554.4	559.4	564.5	509.4	523.1	535.9	76.0
	4	554.2	559.2	564.3	509.6	523.2	536.1	77.0
	5	551.8	556.7	561.7	512.4	526.9	541.0	76.1
	6	551.6	556.5	561.5	512.4	527.0	541.2	76.7
	7	552.4	557.4	562.4	511.7	525.9	539.6	77.1
	8	552.3	557.2	562.2	511.5	526.0	539.5	76.3
IR	1	555.3	563.6	572.2	509.2	525.0	541.7	65.2
	2	554.9	563.4	571.7	509.1	525.0	541.4	65.7
	3	556.7	565.5	574.1	507.0	523.5	538.7	65.2
	4	556.6	565.4	574.0	507.6	523.6	539.1	65.0
	5	553.7	561.6	569.7	509.4	526.3	543.0	66.9
	6	553.2	561.5	569.5	509.7	526.4	543.2	66.5
	7	554.9	563.2	571.6	508.6	524.9	541.0	65.5
	8	553.1	559.2	565.3	510.9	525.7	540.4	72.7

Model	tree	Euarchontoglires mean 95% HPD	Euarchontoglires upper 95% HPD	Euarthropoda lower 95% HPD	Euarthropoda mean 95% HPD	Euarthropoda upper 95% HPD	Eumetabola lower 95% HPD	Eumetabola mean 95% HPD
AC	1	114.3	150.3	530.3	536.0	541.6	350.7	386.2
	2	114.3	151.9	530.3	535.9	541.6	350.0	386.3
	3	114.1	151.4	530.6	536.3	542.0	350.8	386.3
	4	114.2	152.7	530.4	536.2	541.9	350.5	386.3
	5	114.4	151.3	529.8	535.4	540.9	349.8	386.2
	6	114.5	152.3	529.8	535.3	540.9	350.0	386.2
	7	114.3	152.5	530.0	535.7	541.2	350.2	386.2
	8	114.4	151.7	529.8	535.6	541.0	349.9	386.2
IR	1	110.3	153.3	528.8	539.0	549.4	335.0	372.8
	2	110.3	154.0	528.9	539.0	549.7	335.9	372.7
	3	109.8	153.9	529.1	539.6	550.5	336.5	372.8
	4	110.1	153.8	529.0	539.6	550.4	334.8	372.8
	5	110.5	154.8	528.4	538.2	548.4	335.9	372.7
	6	110.5	155.1	528.5	538.3	548.6	336.3	372.9
	7	110.1	153.8	528.7	538.8	549.1	335.7	372.8
	8	113.0	152.8	529.6	536.7	543.9	346.2	381.8

Model	tree	Eumetabola upper 95% HPD	Eumetazoa lower 95% HPD	Eumetazoa mean 95% HPD	Eumetazoa upper 95% HPD	Fungi-Holozoa lower 95% HPD	Fungi-Holozoa mean 95% HPD	Fungi-Holozoa upper 95% HPD	Gastropoda lower 95% HPD	Gastropoda mean 95% HPD
AC	1	421.6	580.6	586.1	591.6	741.0	1084.2	1394.3	480.8	498.7
	2	421.5	579.9	585.3	590.8	737.3	1080.1	1405.5	480.6	498.8
	3	421.1	580.4	585.9	591.3	743.6	1083.3	1399.7	480.5	498.5
	4	421.4	579.7	585.2	590.7	724.7	1074.7	1393.3	480.7	498.5
	5	421.6	580.9	586.4	591.8	743.6	1083.5	1395.9	481.3	499.1
	6	421.4	580.2	585.6	591.1	733.2	1081.6	1404.1	481.2	499.2
	7	421.3	580.6	586.1	591.5	741.4	1081.9	1399.1	481.0	499.0
	8	420.9	580.0	585.4	590.9	727.7	1075.4	1392.3	481.2	499.0
IR	1	407.8	578.9	585.1	591.0	945.2	1212.4	1480.2	472.5	491.0
	2	409.4	578.7	584.9	591.1	941.8	1207.3	1476.5	472.5	491.1
	3	409.4	578.7	584.9	591.1	951.3	1210.9	1476.2	472.5	490.9
	4	408.7	578.6	584.6	590.8	948.3	1206.8	1474.5	472.8	491.0
	5	409.7	579.2	585.3	591.4	959.5	1214.8	1488.2	472.4	491.0
	6	409.8	578.9	585.0	591.2	947.1	1211.2	1480.6	472.0	491.1
	7	408.9	578.9	585.0	591.3	954.0	1212.1	1478.8	472.6	491.0
	8	418.1	579.5	585.2	590.8	794.3	1118.0	1419.9	478.4	496.4

Model	tree	Gastropoda upper 95% HPD	Gnathifera lower 95% HPD	Gnathifera mean 95% HPD	Gnathifera upper 95% HPD	Gnathostomata lower 95% HPD	Gnathostomata mean 95% HPD	Gnathostomata upper 95% HPD	Hemichordata lower 95% HPD
AC	1	515.7	544.0	550.9	557.8	445.0	457.4	470.0	500.8
	2	516.0	543.7	550.8	557.7	444.8	457.4	469.9	501.4
	3	515.6	544.5	551.6	558.3	444.8	457.2	469.8	499.0
	4	515.9	544.5	551.5	558.2	444.8	457.2	469.9	499.4
	5	516.6	541.4	549.0	556.1	444.9	457.5	470.5	503.9
	6	516.8	541.4	548.9	556.1	444.8	457.6	470.3	504.5
	7	516.2	542.1	549.6	557.0	444.8	457.5	470.2	500.8
	8	516.4	542.0	549.5	556.6	445.0	457.5	470.4	501.8
IR	1	509.7	451.9	518.2	562.2	438.1	452.6	467.3	497.7
	2	509.5	450.1	518.5	560.7	438.0	452.6	467.7	498.2
	3	509.4	451.9	519.3	563.7	437.7	452.6	467.3	495.6
	4	509.5	453.4	519.4	563.8	437.8	452.6	467.5	495.6
	5	509.5	447.6	515.6	559.3	438.4	452.7	467.9	500.2
	6	509.5	448.6	516.2	560.3	437.7	452.6	467.6	499.7
	7	509.4	450.8	517.0	560.6	437.8	452.6	467.6	497.2
	8	514.4	511.0	538.8	558.3	442.5	455.9	469.2	500.6

Model	tree	Hemichordata mean 95% HPD	Hemichordata upper 95% HPD	Lobopoda lower 95% HPD	Lobopoda mean 95% HPD	Lobopoda upper 95% HPD	Mammalia lower 95% HPD	Mammalia mean 95% HPD	Mammalia upper 95% HPD	Mandibulata lower 95% HPD
AC	1	521.5	540.3	540.3	545.9	551.6	188.6	225.5	260.7	526.4
	2	521.7	540.5	540.2	545.8	551.4	189.0	225.2	261.1	526.3
	3	519.4	537.9	540.8	546.5	552.2	189.8	225.7	261.7	526.5
	4	519.6	538.1	540.5	546.3	552.1	189.9	225.4	262.0	526.5
	5	525.8	545.8	539.6	545.0	550.6	189.1	225.2	261.5	525.9
	6	526.1	546.0	539.4	544.9	550.3	188.1	225.0	260.6	525.8
	7	523.1	542.4	539.8	545.4	550.9	189.0	225.4	261.2	526.1
	8	523.4	543.2	539.8	545.3	550.9	188.6	225.2	261.2	526.0
IR	1	522.7	546.9	538.5	548.8	558.5	172.2	210.7	250.5	519.5
	2	523.0	547.2	538.7	548.7	558.6	171.6	210.8	250.1	519.6
	3	519.9	543.6	539.6	549.8	560.3	172.2	210.6	250.0	519.8
	4	520.0	543.0	539.6	549.8	560.1	172.5	210.9	249.4	519.8
	5	525.9	551.2	537.8	547.6	557.1	172.3	210.8	249.5	519.4
	6	526.1	551.2	538.1	547.6	557.4	172.2	210.8	250.1	519.6
	7	522.5	547.0	538.2	548.5	558.1	171.8	210.7	249.5	519.7
	8	523.2	544.8	539.4	546.4	553.4	183.5	220.4	257.5	523.9

Model	tree	Mandibulata mean 95% HPD	Mandibulata upper 95% HPD	Metazoa lower 95% HPD	Metazoa mean 95% HPD	Metazoa upper 95% HPD	Mollusca lower 95% HPD	Mollusca mean 95% HPD	Mollusca upper 95% HPD	Nematoda-Arthropoda lower 95% HPD
AC	1	532.0	537.8	590.6	597.9	605.3	534.4	539.8	545.0	548.2
	2	531.9	537.6	595.0	604.0	613.5	534.4	539.8	545.1	548.1
	3	532.3	538.0	590.8	598.0	605.4	534.7	540.1	545.4	549.1
	4	532.3	538.1	595.1	604.2	613.5	534.7	540.1	545.4	549.0
	5	531.5	537.1	590.9	598.1	605.5	533.8	539.1	544.4	547.0
	6	531.5	537.1	595.2	604.1	613.6	533.8	539.0	544.3	546.7
	7	531.7	537.4	590.8	598.2	605.5	534.0	539.3	544.6	547.5
	8	531.7	537.4	595.3	604.2	613.6	533.9	539.3	544.6	547.5
IR	1	526.8	534.0	594.3	604.6	615.9	534.6	541.6	548.9	547.4
	2	526.8	534.0	595.9	607.0	618.9	534.5	541.6	548.9	547.3
	3	527.0	534.2	594.0	604.7	615.7	534.7	541.9	549.3	548.7
	4	527.0	534.3	595.7	607.1	619.0	534.6	541.9	549.1	548.5
	5	526.6	533.7	594.1	604.6	615.7	534.4	541.3	548.5	546.2
	6	526.6	533.8	595.8	607.0	618.8	534.2	541.3	548.4	546.3
	7	526.8	534.0	594.2	604.7	615.9	534.5	541.5	548.7	547.4
	8	530.0	536.3	595.5	605.1	615.5	534.2	540.0	546.0	547.4

Model	tree	Nematoda-Arthropoda mean 95% HPD	Nematoda-Arthropoda upper 95% HPD	Nephrozoa lower 95% HPD	Nephrozoa mean 95% HPD	Nephrozoa upper 95% HPD	Olfactores lower 95% HPD	Olfactores mean 95% HPD	Olfactores upper 95% HPD	Osteichthyes lower 95% HPD
AC	1	553.5	558.7	566.3	571.4	576.6	543.1	551.4	559.3	417.6
	2	553.3	558.6	565.9	571.1	576.2	543.2	551.3	559.3	417.8
	3	554.4	559.7	NA	NA	NA	543.4	551.7	559.7	417.8
	4	554.2	559.6	NA	NA	NA	543.5	551.7	559.6	417.7
	5	552.0	557.3	564.2	569.4	574.4	545.7	553.7	561.4	417.9
	6	551.9	556.9	564.1	569.1	574.1	545.9	553.6	561.4	418.0
	7	552.7	557.9	565.4	570.5	575.6	546.0	554.1	561.9	417.6
	8	552.5	557.7	565.1	570.2	575.3	545.8	554.0	561.6	417.8
IR	1	556.8	565.9	569.6	576.3	582.5	524.9	541.3	557.9	420.2
	2	556.7	565.7	569.4	576.0	582.4	524.2	541.3	557.4	420.0
	3	558.3	567.8	NA	NA	NA	525.2	542.7	559.2	420.1
	4	558.2	567.5	NA	NA	NA	525.5	542.8	559.5	419.9
	5	555.2	564.0	566.6	573.2	579.6	526.1	543.6	560.1	420.1
	6	555.2	564.2	566.4	573.0	579.4	526.2	543.7	560.1	420.0
	7	556.5	565.9	569.1	575.7	582.2	526.6	544.7	561.4	420.3
	8	553.8	560.4	566.4	571.9	577.6	539.7	550.9	562.1	418.7

Model	tree	Osteichthyes mean 95% HPD	Osteichthyes upper 95% HPD	Pancrustacea lower 95% HPD	Pancrustacea mean 95% HPD	Pancrustacea upper 95% HPD	Placozoa-other-Metazoa lower 95%	Placozoa-other-Metazoa mean 95%	Placozoa-other-Metazoa upper 95%
AC	1	428.5	439.3	510.6	517.0	523.2	584.7	590.4	596.4
	2	428.6	439.4	510.6	517.0	523.4	583.5	589.3	595.0
	3	428.4	439.4	510.6	517.0	523.4	584.4	590.2	596.0
	4	428.5	439.3	510.7	517.0	523.5	583.4	589.2	595.0
	5	428.6	439.6	510.5	516.9	523.1	584.8	590.6	596.4
	6	428.7	439.6	510.6	517.0	523.4	584.0	589.6	595.4
	7	428.5	439.3	510.6	516.9	523.4	584.7	590.4	596.2
	8	428.6	439.5	510.6	517.0	523.3	583.8	589.4	595.2
IR	1	431.1	442.1	510.5	517.6	524.7	582.6	589.2	595.4
	2	431.2	442.0	510.4	517.6	524.7	582.5	588.9	595.2
	3	431.1	442.1	510.4	517.6	524.7	582.6	589.1	595.5
	4	431.1	442.0	510.5	517.7	524.9	582.3	588.7	595.1
	5	431.2	442.1	510.3	517.5	524.5	583.1	589.3	595.8
	6	431.2	442.1	510.5	517.6	524.6	582.6	589.0	595.4
	7	431.2	442.1	510.5	517.6	524.9	582.6	589.2	595.5
	8	429.5	440.4	510.6	517.2	523.8	583.4	589.2	595.3

Model	tree	Porifera lower 95% HPD	Porifera mean 95% HPD	Porifera upper 95% HPD	Porifera-other-Metazoa lower 95% HPD	Porifera-other-Metazoa mean 95% HPD	Porifera-other-Metazoa upper 95% HPD	Protostomia lower 95% HPD	Protostomia mean 95% HPD	Protostomia upper 95% HPD
AC	1	524.1	549.7	571.7	NA	NA	NA	558.7	563.8	568.7
	2	525.6	549.5	570.9	586.7	592.9	599.1	558.5	563.5	568.5
	3	524.3	548.8	571.4	NA	NA	NA	560.1	565.1	570.1
	4	525.4	548.7	570.0	586.8	593.0	599.3	559.8	564.9	569.8
	5	525.2	550.3	573.0	NA	NA	NA	556.9	561.8	566.8
	6	526.1	550.2	571.6	587.1	593.2	599.4	556.7	561.5	566.4
	7	524.1	549.6	572.0	NA	NA	NA	557.8	562.6	567.7
	8	525.8	549.6	571.1	587.1	593.2	599.4	557.5	562.4	567.3
IR	1	517.3	547.3	577.1	NA	NA	NA	564.1	570.8	577.6
	2	516.1	544.5	572.3	586.7	593.9	601.0	563.7	570.6	577.5
	3	517.2	546.9	576.1	NA	NA	NA	566.3	573.4	580.3
	4	516.2	544.3	572.4	586.7	593.9	601.0	566.1	573.2	580.2
	5	517.8	547.4	576.5	NA	NA	NA	561.6	568.3	575.0
	6	515.9	544.8	572.4	587.0	594.0	601.3	561.3	568.2	574.9
	7	516.4	547.2	576.0	NA	NA	NA	563.4	570.4	577.1
	8	522.0	547.9	570.7	587.1	593.5	600.1	559.3	565.0	570.5

Model	tree	Pycnogonida-other-Chelicerates lower 95%	Pycnogonida-other-Chelicerates mean 95%	Pycnogonida-other-Chelicerates upper 95%	Rotifera lower 95% HPD	Rotifera mean 95% HPD	Rotifera upper 95% HPD	Spiralia lower 95% HPD	Spiralia mean 95% HPD	Spiralia upper 95% HPD
AC	1	517.3	525.4	533.1	435.2	502.6	540.4	555.1	560.0	564.9
	2	517.4	525.4	533.2	437.6	503.7	540.4	554.9	559.7	564.7
	3	517.2	525.4	533.4	432.4	501.3	540.0	556.1	561.1	565.9
	4	517.1	525.4	533.3	436.2	502.5	540.0	555.9	560.8	565.8
	5	517.3	525.3	532.9	436.5	502.6	540.2	553.0	557.9	562.8
	6	517.4	525.3	532.8	436.4	502.9	539.7	553.0	557.8	562.6
	7	517.6	525.4	533.2	433.8	501.9	539.5	553.8	558.7	563.6
	8	517.4	525.4	533.1	436.0	502.4	539.9	553.7	558.5	563.4
IR	1	506.3	524.0	539.7	216.7	385.6	522.4	555.4	564.0	571.8
	2	507.3	524.1	540.8	215.9	386.6	526.4	555.5	563.9	571.9
	3	506.8	524.2	540.8	215.8	386.0	526.9	557.3	565.9	574.4
	4	507.0	524.3	541.1	213.2	386.3	522.0	557.3	565.8	574.4
	5	506.3	523.5	539.0	204.5	380.6	523.1	554.1	561.9	569.8
	6	506.7	523.6	539.5	202.0	382.3	523.9	553.8	561.8	569.7
	7	506.6	523.8	540.1	215.3	383.6	523.3	555.3	563.5	571.5
	8	513.9	524.9	535.6	360.9	463.4	535.5	554.2	560.2	566.2

Model	tree	Tetrapoda lower 95% HPD	Tetrapoda mean 95% HPD	Tetrapoda upper 95% HPD	Vertebrata lower 95% HPD	Vertebrata mean 95% HPD	Vertebrata upper 95% HPD	Xenacoelomorpha lower 95% HPD	Xenacoelomorpha mean 95% HPD
AC	1	341.9	348.7	355.8	492.8	510.4	527.5	553.1	562.9
	2	341.7	348.6	355.7	492.7	510.5	527.5	553.2	562.9
	3	341.8	348.8	355.8	492.4	510.0	527.0	531.7	548.7
	4	341.7	348.7	355.8	492.7	510.2	527.6	531.4	548.6
	5	341.7	348.6	355.7	494.4	512.4	530.0	553.7	563.4
	6	341.4	348.5	355.4	494.4	512.7	530.2	553.6	563.4
	7	341.8	348.7	355.7	494.6	512.3	530.2	544.0	555.4
	8	341.6	348.6	355.5	494.3	512.5	530.0	543.8	555.3
IR	1	336.2	343.7	351.3	488.4	513.4	536.5	374.7	502.6
	2	336.1	343.7	351.1	489.5	513.6	537.3	375.5	502.6
	3	336.2	343.7	351.1	489.0	514.0	537.4	347.5	484.0
	4	336.2	343.7	351.2	489.6	514.3	538.2	341.8	483.1
	5	336.3	343.7	351.3	490.3	515.0	538.8	376.3	503.4
	6	336.2	343.7	351.2	490.1	515.3	539.2	374.5	503.2
	7	336.3	343.7	351.3	490.3	515.3	539.7	352.6	490.4
	8	339.8	347.0	354.2	493.3	513.5	533.5	480.6	533.7

Model	tree	Xenacoelomorpha upper 95% HPD	Xenambulacraria lower 95%HPD	Xenambulacraria mean 95%HPD	Xenambulacraria upper 95%HPD
AC	1	572.3	NA	NA	NA
	2	572.1	NA	NA	NA
	3	560.5	558.6	564.1	569.6
	4	560.3	558.4	563.9	569.4
	5	573.2	NA	NA	NA
	6	572.9	NA	NA	NA
	7	566.8	564.0	569.3	574.6
	8	566.6	563.8	569.1	574.4
IR	1	574.8	NA	NA	NA
	2	575.9	NA	NA	NA
	3	556.7	549.9	561.7	572.7
	4	555.0	549.3	561.6	572.2
	5	576.7	NA	NA	NA
	6	576.6	NA	NA	NA
	7	564.4	555.4	567.8	578.9
	8	566.2	561.1	568.6	576.0

Supplementary Table S3: Age estimates from all of the hypotheses tested under the Normal calibration scheme. Lower, upper and mean 95% HPD intervals are provided for all named nodes in all trees.

Supplementary Calibration justifications

Node: Fungi - Holozoa | 1631.2 – 573.51

Node calibrated: Opisthokonta, the clade composed of Fungi and Metazoa.

Fossil taxon: *Charnia masoni* (LEIUG 2328 from Bed B of North Quarry, Charnwood Forest, UK).

Minimum age: The earliest occurrence of *Charnia masoni* is in the Drook Formation of Mistaken Point, Newfoundland, which has been dated to 574.17 Ma ± 0.66 Myr, yielding a minimum age constraint of 573.51 Ma (*62*).

Minimum justification: Rangeomorphs such as *Charnia masoni* are interpreted as stem eumetazoans, positioned after Placozoa and before Cnidaria/Ctenophora in the metazoan tree (*22*). Given the regular and predictable morphology of *Charnia*, the consistent differentiation of new elements, and evidence of transitions in the primary developmental node, Dunn and colleagues (*22*) established that *Charnia* was at least total group metazoan. Additionally, they determined that *Charnia* has body regionalisation, a trait found in all animals except placozoans and poriferans, and two body axes as seen in cnidarians and ctenophores.

Maximum age: 1631.5 Ma, based on the maximum age of the Chuanlinggou Formation, dated to 1625.3 ± 6.2 Myr (*146*).

Maximum justification: Following Betts et al. (*147*).

Node: Filasteria - Choanozoa | 1631.2 – 573.51

Node calibrated: The clade defined by *Capsaspora, Homo*, their last common ancestor and all of its descendants.

Fossil taxon: *Charnia masoni* (LEIUG 2328 from Bed B of North Quarry, Charnwood Forest, UK).

Minimum age: The earliest occurrence of *Charnia masoni* is in the Drook Formation of Mistaken Point, Newfoundland, which has been dated to 574.17 Ma ± 0.66 Myr, yielding a minimum age constraint of 573.51 Ma (*62*).

Minimum justification: Rangeomorphs such as *Charnia masoni* are interpreted as stem eumetazoans, positioned after Placozoa and before Cnidaria/Ctenophora in the metazoan tree (*22*). Given the regular and predictable morphology of *Charnia*, the consistent differentiation of new elements, and evidence of transitions in the primary developmental node, Dunn and colleagues (*22*) established that *Charnia* was at least total group metazoan. Additionally, they determined that *Charnia* has body regionalisation, a trait found in all animals except placozoans and poriferans, and two body axes as seen in cnidarians and ctenophores.

Maximum age: 1631.5 Ma, based on the maximum age of the Chuanlinggou Formation, dated to 1625.3 ± 6.2 Myr (*146*).

Maximum justification: Following Betts et al. (*147*).

Node: Choanoflagellata - Metazoa | 1631.2 – 573.51

Node calibrated: Choanozoa, the clade composed of choanoflagellates and metazoans.

Fossil taxon: *Charnia masoni* (LEIUG 2328 from Bed B of North Quarry, Charnwood Forest, UK).

Minimum age: The earliest occurrence of *Charnia masoni* is in the Drook Formation of Mistaken Point, Newfoundland, which has been dated to 574.17 Ma ± 0.66 Myr, yielding a minimum age constraint of 573.51 Ma (*62*).

Minimum justification: Rangeomorphs such as *Charnia masoni* are interpreted as stem eumetazoans, positioned after Placozoa and before Cnidaria/Ctenophora in the metazoan tree (*22*). Given the regular and predictable morphology of *Charnia*, the consistent differentiation of new elements, and evidence of transitions in the primary developmental node, Dunn and colleagues (*22*) established that *Charnia* was at least total group metazoan. Additionally, they determined that *Charnia* has body regionalisation, a trait found in all animals except placozoans and poriferans, and two body axes as seen in cnidarians and ctenophores.

Maximum age: 1631.5 Ma, based on the maximum age of the Chuanlinggou Formation, dated to 1625.3 ± 6.2 Myr (*146*).

Node calibrated: The clade defined by *Capsaspora, Homo*, their last common ancestor and all of its descendants.

Fossil taxon: *Charnia masoni* (LEIUG 2328 from Bed B of North Quarry, Charnwood Forest, UK).

Minimum age: The earliest occurrence of *Charnia masoni* is in the Drook Formation of Mistaken Point, Newfoundland, which has been dated to 574.17 Ma ± 0.66 Myr, yielding a minimum age constraint of 573.51 Ma (*62*).

Minimum justification: Rangeomorphs such as *Charnia masoni* are interpreted as stem eumetazoans, positioned after Placozoa and before Cnidaria/Ctenophora in the metazoan tree (*22*). Given the regular and predictable morphology of *Charnia*, the consistent differentiation of new elements, and evidence of transitions in the primary developmental node, Dunn and colleagues (*22*) established that *Charnia* was at least total group metazoan. Additionally, they determined that *Charnia* has body regionalisation, a trait found in all animals except placozoans and poriferans, and two body axes as seen in cnidarians and ctenophores.

Maximum age: 1631.5 Ma, based on the maximum age of the Chuanlinggou Formation, dated to 1625.3 ± 6.2 Myr (*146*).

Node: Crown Metazoa | 609 - 573.51Ma

Node calibrated: Porifera, Ctenophora, Placozoa, Cnidaria and Bilateria. The arrangement of these nodes within the clade varies by tree, but all together form Metazoa.

Fossil taxon: *Charnia masoni* (LEIUG 2328 from Bed B of North Quarry, Charnwood Forest, UK).

Minimum age: The earliest occurrence of *Charnia masoni* is in the Drook Formation of Mistaken Point, Newfoundland, which has been dated to 574.17 Ma ± 0.66 Myr, yielding a minimum age constraint of 573.51 Ma (*62*).

Minimum justification: Rangeomorphs such as *Charnia masoni* are interpreted as stem eumetazoans, positioned after Placozoa and before Cnidaria/Ctenophora in the metazoan tree (*22*). Given the regular and predictable morphology of *Charnia*, the consistent differentiation of

new elements, and evidence of transitions in the primary developmental node, Dunn and colleagues (*22*) established that *Charnia* was at least total group metazoan. Additionally, they determined that *Charnia* has body regionalisation, a trait found in all animals except placozoans and poriferans, and two body axes as seen in cnidarians and ctenophores.

Maximum age: 609 Ma Lantian Biota (55)

Maximum justification: The Lantian Biota yields some of the oldest known macrofossils and specimens have been interpreted as algae and putative animals (54). However, the putative animals have not been investigated enough to determine whether they are in fact metazoans, or whether they are algae. The extensive macrofossils of algae demonstrate adequate fossilisation conditions, and we can therefore say that crown metazoans were absent.

Discussion: There are several alternatives that could have been used for this node. *Eoandromeda* has been suggested to be a stem-group ctenophore from the Doushantuo Formation of southern China (*16*), but others have suggested it is too different from other stem ctenophores to be one itself, although it may be a stem coelenterate (*20*). Others have suggested a protistan affinity or possibly stem-group eumetazoan (*18, 19*). Although the Doushantuo Formation is dated at 585.7 ± 2.8 Ma, making it older than *Charnia*, the uncertainty around its affinity means it is not a suitable calibration.

Possible sponge biomarkers from between the Sturtian and Marinoan glaciations (minimum age of 634.97 Ma) have also been discovered and suggested as the earliest evidence of metazoans (*3*), but more recent work has demonstrated that these biomarkers can also be produced by bacteria (*5, 149*). Vermiform microstructures from ~890 million year old reefs have also been suggested to be from sponges, but other possibilities such as branching cyanobacteria or algae have not yet been ruled out (2).

Dickinsonia and *Kimberella*, both from the White Sea Biota, are also putative animals. *Kimberella* has features of a bilateral metazoan including an anterior-posterior axis and possible surrounding feeding traces, while *Dickinsonia* has been interpreted as a placozoan-grade

organism (*150, 157*). However, being placazoan-grade does not necessarily mean it was within the placazoan clade, and although *Kimberella* has previously been interpreted as a stem mollusc, others have suggested a coelenterate affinity (*67*) or interpreted as simply a probable bilaterian (*68*). Regardless, both *Kimberella* and *Dickinsonia* are younger than *Charnia* at 552.3 Ma (*17*).

Trace fossils that were interpreted as evidence of locomotion, likely from metazoan-grade organisms such as cnidarian polyps, from 565 Ma are both younger than *Charnia* and of uncertain origin (*167*).

Node: Crown Eumetazoa | 590.8 - 561.1 Ma

Node calibrated: Cnidaria, Bilateria, Placozoa, and either Ctenophora or Porifera, depending on tree hypothesis. The calibration does not change by tree (it is not based on either a ctenophore or a poriferan)

Fossil taxon: *Auroralumina attenboroughii (*GSM 106119) from Bed B, Bradgate Formation, Charnian Supergroup of North Quarry, Charnwood Forest, UK (*59*)

Minimum age: Bed B, Bradgate Formation, Charnian Supergroup, 563 ±1.9 Ma, so 561.1 Ma (*65*). More recently, the U-Pb-derived age of 556.6 Ma ± 6.4 Myr was suggested (*64*), but the associated uncertainty entirely encompasses the original, more precise date, and so we follow the earlier date from Wilby *et al.* (*65*).

Minimum justification: Formal character analysis and phylogenetic tests placed *Auroralumina attenboroughii* as a crown-cnidarian, likely a stem-group medusozoan (*59*).

Maximum age: Weng'an Biota, 590.8 Ma (*17*). Yang *et al.* (*17*) date the biota based on Re-Os data to 587.2 Ma ±3.6 Myr, yielding a maximum age constraint of 590.8 Ma.

Maximum justification: The Weng'an Biota yields exceptionally preserved macrofossils of algae but nothing that can definitively be placed into crown Metazoa. Embryo-like fossils preserved

here have been suggested to be total group metazoans (*99*), but this is still uncertain, and others have suggested they are non-metazoan holozoans (*11*).

Discussion: There are several alternatives that could have been used for this node. *Eoandromeda* has been suggested to be a stem-group ctenophore from the Doushantuo Formation of southern China (*16*), but others have suggested it is too different from other stem ctenophores to be one itself, although it may be a stem coelenterate (*20*). Others have suggested a protistan affinity or possibly stem-group eumetazoan (*18, 19*), meaning it is too uncertain to be a reliable minimum calibration.

Dickinsonia and *Kimberella*, both from the White Sea Biota, are also putative animals. *Kimberella* has features of a bilateral metazoan including an anterior-posterior axis and possible surrounding feeding traces, while *Dickinsonia* has been interpreted as a placozoan-grade organism (*150, 157*). However, being placazoan-grade does not necessarily mean it was within the placazoan clade, and although *Kimberella* has previously been interpreted as a stem mollusc, others have suggested a coelenterate affinity (*67*) or interpreted it as simply a probable bilaterian (*68*). Regardless, both *Kimberella* and *Dickinsonia* are younger than *Auroralumina* at 552.3 Ma (*17*).

Trace fossils that were interpreted as evidence of locomotion, likely from metazoan-grade organisms such as cnidarian polyps, from 565 Ma are both younger than *Auroralumina* and of uncertain origin (*167*).

The Lantian Biota also has extensive macrofossils without any definitive metazoans, but it is older than the Weng'an Biota (*17*), making the Weng'an a more reasonable choice.

Node: Crown Bilateria | 590.8 - 532 Ma

Node calibrated: Xenacoelomorpha and Nephrozoa (Protostomes + Deuterostomes)

Fossil taxon: *Aldanella janjiahensis* (YXII102-02), often synonymised with *Aldanella attleborensis*) from the Lower Cambrian Dahai Member of Zhujiaqing Formation, Xiaotan, Yongshan County, Yunnan (*31*)

Minimum age: 532 Ma (*31*). The fossil is found within the *Anabarites trisulcatus-Protohertzina anabarica* Assemblage Biozone, which falls within the age range of 537-532 Ma (*72*).

Minimum justification: The dextrally coiled *Aldanella* was assigned to Pelagiellida, and based on the morphology and preservation of muscle scars can be identified as a stem-group gastropod (*71*).

Maximum age: Weng'an Biota, 590.8 Ma (*17*). Yang *et al.* (*17*) date the biota based on Re-Os data to 587.2 Ma ±3.6 Myr, yielding a maximum age constraint of 590.8 Ma.

Maximum justification: The Weng'an Biota yields exceptionally preserved macrofossils of algae but nothing that can definitively be placed into crown Metazoa. Embryo-like fossils preserved here have been suggested to be total group metazoans (*99*), but this is still uncertain, and others have suggested they are non-metazoan holozoans (*11*).

Discussion: *Kimberella* from the White Sea Biota, is also a putative bilaterian. *Kimberella* has features of a bilateral metazoan including an anterior-posterior axis and possible surrounding feeding traces, but has also been suggested to be a coelenterate (*67*) or interpreted as simply a probable bilaterian (*68*). This uncertainty makes *Kimberella* an unsuitable choice for the minimum calibration. Similarly, the jaw apparatus of *Redkinia,* from the mid-Vendian of the Russian Platform, has variably been interpreted as being from a polychaete, a stem-arthropod, or a crown arthropod, and the age of the formation it comes from has not been adequately dated (*151, 70*).

Amiskwia sagittiformis is a possible chaetognath or crown gnathiferan, meaning a crown bilaterian (*152, 153*), but it is much younger than *Aldanella* at 508 Ma.

The Lantian Biota also has extensive macrofossils without any definitive metazoans, but it is older than the Weng'an Biota (*17*), making the Weng'an a more reasonable choice.

Node: Crown Protostomia | 590.8 - 532 Ma

Node calibrated: Lophotrochozoa (Spiralia) and Ecdysozoa

Fossil taxon: *Aldanella janjiahensis* (YXII102-02), often synonymised with *Aldanella attleborensis*) from the Lower Cambrian Dahai Member of Zhujiaqing Formation, Xiaotan, Yongshan County, Yunnan (*31*).

Minimum age: 532 Ma (*31*). The fossil is found within the *Anabarites trisulcatus-Protohertzina anabarica* Assemblage Biozone, which falls within the age range of 537-532 Ma (*72*).

Minimum justification: The dextrally coiled *Aldanella* was assigned to Pelagiellida, and based on the morphology and preservation of muscle scars can be identified as a stem-group gastropod (*71*).

Maximum age: Weng'an Biota, 590.8 Ma (*17*). Yang *et al.* (*17*) date the biota based on Re-Os data to 587.2 Ma ±3.6 Myr, yielding a maximum age constraint of 590.8 Ma.

Maximum justification: The Weng'an Biota yields exceptionally preserved macrofossils of algae but nothing that can definitively be placed into crown Metazoa. Embryo-like fossils preserved here have been suggested to be total group metazoans (*99*), but this is still uncertain, and others have suggested they are non-metazoan holozoans (*11*).

Discussion: *Kimberella* from the White Sea Biota, is also a putative protostome. *Kimberella* has features of a bilateral metazoan including an anterior-posterior axis and possible surrounding feeding traces, but has also been suggested to be a coelenterate (*67*) or interpreted as simply a probable bilaterian (*68*). This uncertainty makes *Kimberella* an unsuitable choice for the minimum calibration. Similarly, the jaw apparatus of *Redkinia,* from the mid-Vendian of the Russian Platform, has variably been interpreted as being from a polychaete, a stem-arthropod, or a crown arthropod, and the age of the formation it comes from has not been adequately dated (*151, 70*).

Namacalathus is a goblet-shaped stalked organism that has been interpreted as a lophotrochozoan (*154, 155*). However, there have been numerous other interpretations for this animal, ranging from cnidarian to stem eumetazoan, and this uncertainty makes it an unsuitable calibration (*154*).

The Lantian Biota also has extensive macrofossils without any definitive metazoans, but it is older than the Weng'an Biota (*17*), making the Weng'an a more reasonable choice.

Node: Spiralia | 590.8 - 532 Ma

Node calibrated: Last common ancestor of the clade encompassing Annelida, Mollusca and Chaetognatha.

Fossil taxon: *Aldanella janjiahensis* (YXII102-02), often synonymised with *Aldanella attleborensis*) from the Lower Cambrian Dahai Member of Zhujiaqing Formation, Xiaotan, Yongshan County, Yunnan (*31*).

Minimum age: 532 Ma (*31*). The fossil is found within the *Anabarites trisulcatus-Protohertzina anabarica* Assemblage Biozone, which falls within the age range of 537-532 Ma (*72*).

Minimum justification: The dextrally coiled *Aldanella* was assigned to Pelagiellida, and based on the morphology and preservation of muscle scars can be identified as a stem-group gastropod (*71*).

Maximum age: Weng'an Biota, 590.8 Ma (*17*). Yang *et al.* (*17*) date the biota based on Re-Os data to 587.2 Ma ±3.6 Myr, yielding a maximum age constraint of 590.8 Ma.

Maximum justification: The Weng'an Biota yields exceptionally preserved macrofossils of algae but nothing that can definitively be placed into crown Metazoa. Embryo-like fossils preserved here have been suggested to be total group metazoans (*99*), but this is still uncertain, and others have suggested they are non-metazoan holozoans (*11*).

Discussion: *Dannychaeta tucolus* is described as the oldest crown annelid, placed within Palaeoannelida (a polychaete group) (*156*). However, it is younger than *Aldanella* at 514 Ma.

The Lantian Biota also has extensive macrofossils without any definitive metazoans, but it is older than the Weng'an Biota (*17*), making the Weng'an a more reasonable choice.

Node: Capitellid-Polychaete-Leech | 590.8 - 514 Ma

Our minimum constraint follows (*157*)

Node calibrated: Last common ancestor of the clade encompassing *Capitella*, Polychaeta and *Clitellata*.

Fossil taxon: *Dannychaeta tucolus* (YKLP 11382), Yunnan Key Laboratory for Palaeobiology (*156*)

Minimum age: 514 Ma, from the Canglangpu Formation, Cambrian stage 3, Hongjingshao Member, southwest of Guanshan reservoir, Chenggong, Kunming, China (*156*),

Minimum justification: *Dannychaeta* has shared characteristics with extant Magelonidae including a well-defined spade-shaped prostomium, a wide and stout thorax, and biramous parapodia with parapodial lamellae. Phylogenetic analyses placed it within crown Annelida (*156*)

Maximum age: Weng'an Biota, 590.8 Ma (*17*). Yang *et al.* (*17*) date the biota based on Re-Os data to 587.2 Ma ±3.6 Myr, yielding a maximum age constraint of 590.8 Ma.

Maximum justification: The Weng'an Biota yields exceptionally preserved macrofossils of algae but nothing that can definitively be placed into crown Metazoa. Embryo-like fossils preserved here have been suggested to be total group metazoans (*99*), but this is still uncertain, and others have suggested they are non-metazoan holozoans (*11*).

Discussion: The scolecodont *'Xanioprion' viivei* is suggested to be the jaw apparatus of a polychaete annelid, however it is younger than *Dannychaeta* at 476.5 Ma (*157*).

The Lantian Biota also has extensive macrofossils without any definitive metazoans, but it is older than the Weng'an Biota (*17*), making the Weng'an a more reasonable choice.

Node: Crown Mollusca | 549 - 532 Ma

Our minimum and maximum constraints follow (*157*)

Node calibrated: Last common ancestor of the clade encompassing Bivalvia, Gastropoda and Cephalopoda.

Fossil taxon: *Aldanella janjiahensis* (YXII102-02), often synonymised with *Aldanella attleborensis*) from the Lower Cambrian Dahai Member of Zhujiaqing Formation, Xiaotan, Yongshan County, Yunnan (*31*).

Minimum age: 532 Ma (*31*). The fossil is found within the *Anabarites trisulcatus-Protohertzina anabarica* Assemblage Biozone, which falls within the age range of 537-532 Ma (*72*).

Minimum justification: The dextrally coiled *Aldanella* was assigned to Pelagiellida, and based on the morphology and preservation of muscle scars can be identified as a stem-group gastropod (*71*).

Maximum age: Nama Group, 549 Ma (*78*)

Maximum justification: The Nama Group is an open marine community that yields some of the earliest animal skeletal remains such as *Cloudina*. However, there are no crown group molluscs present, despite preservation clearly being capable of preserving hard remains (*78, 157*)

Discussion: Internal relationships among molluscs are still uncertain, making it difficult to determine whether fossils belong to the stem or crown (*157*). *Aldanella* is definitively gastropod in affinity due to the dextrally coiled shell.

The Weng'an Biota yields exceptionally preserved macrofossils of algae but nothing that can definitively be placed into crown Metazoa. Embryo-like fossils preserved here have been suggested to be total group metazoans (*99*), but this is still uncertain, and others have suggested they are non-metazoan holozoans (*11*).

Node: Bivalve-Gastropod | 549 - 532 Ma

Our minimum and maximum constraints follow (*157*) for crown Mollusca

Node calibrated: Last common ancestor of the clade encompassing Bivalvia and Gastropoda.

Fossil taxon: *Aldanella janjiahensis* (YXII102-02), often synonymised with *Aldanella attleborensis*) from the Lower Cambrian Dahai Member of Zhujiaqing Formation, Xiaotan, Yongshan County, Yunnan (*31*).

Minimum age: 532 Ma (*31*). The fossil is found within the *Anabarites trisulcatus-Protohertzina anabarica* Assemblage Biozone, which falls within the age range of 537-532 Ma (*72*)

Minimum justification: The dextrally coiled *Aldanella* was assigned to Pelagiellida, and based on the morphology and preservation of muscle scars can be identified as a stem-group gastropod (*71*).

Maximum age: Nama Group, 549 Ma (*78*)

Maximum justification: The Nama Group is an open marine community that yields some of the earliest animal skeletal remains such as *Cloudina*. However, there are no crown group molluscs present, despite preservation clearly being capable of preserving hard remains (*78, 157*)

Discussion: Internal relationships among molluscs are still uncertain, making it difficult to determine whether fossils belong to the stem or crown (*157*). *Aldanella* is definitively gastropod in affinity due to the dextrally coiled shell.

The Weng'an Biota yields exceptionally preserved macrofossils of algae but nothing that can definitively be placed into crown Metazoa. Embryo-like fossils preserved here have been suggested to be total group metazoans (*99*), but this is still uncertain, and others have suggested they are non-metazoan holozoans (*11*).

Node: Crown Gastropoda | 531.5 - 470.2 Ma

Our minimum and maximum constraints follow (*91*)

Node calibrated: Last common ancestor of the clade encompassing all extant Gastropoda.

Fossil taxon: *Turritoma acrea* (GSC 585, Geological Survey of Canada) (*158*)

Minimum age: 470.2 Ma (*91, 158*).

Minimum justification: *Turritoma acrea* is the oldest accepted member of the Eotomarioidea, a crown gastropod group (*91, 158*).

Maximum age: 531.5 Ma (*91*).

Maximum justification: *Latouchella* is described as the oldest Mollusc, and since it can be preserved as a fossil but no crown gastropods are found from the same age, it demonstrates that gastropods were not present yet (*91*).

Discussion: Internal relationships among molluscs are still uncertain, making it difficult to determine whether fossils belong to the stem or crown (*91*).

The Weng'an Biota yields exceptionally preserved macrofossils of algae but nothing that can definitively be placed into crown Metazoa. Embryo-like fossils preserved here have been suggested to be total group metazoans (*99*), but this is still uncertain, and others have suggested they are non-metazoan holozoans (*11*).

Node: Crown Ecdysozoa | 590.8 - 531.8 Ma

Node calibrated: Last common ancestor of the clade encompassing Arthropoda, Onychophora, Nematoda and Priapulida.

Fossil taxon: *Eopriapulites* (CGM16, deposited in the Geological Museum of Chang'an University, China) from the Kuanchuanpu Formation (*29, 159*).

Minimum age: The early Cambrian Kuanchuanpu Formation has been dated to 536.4 - 531.8 Ma (*72*), yielding a minimum age of 531.8 Ma for this node.

Minimum justification: Several fossils from the Kuanchuanpu Formation can be placed into total group Scalidophora, including *Eopriapulites* and *Eokinorhynchus*, and putative exuviae have also been discovered (*29, 159*). Together these present definitive evidence for the presence of crown Ecdysozoans in the earliest Cambrian.

Maximum age: Weng'an Biota, 590.8 Ma (*17*). Yang *et al.* (*17*) date the biota based on Re-Os data to 587.2 Ma ±3.6 Myr, yielding a maximum age constraint of 590.8 Ma.

Maximum justification: The Weng'an Biota yields exceptionally preserved macrofossils of algae but nothing that can definitively be placed into crown Metazoa. Embryo-like fossils preserved

here have been suggested to be total group metazoans (*99*), but this is still uncertain, and others have suggested they are non-metazoan holozoans (*11*).

Discussion: *Rusophycus* trace fossils are widely accepted to have been created by arthropod-grade organisms on the basis of the perceived bilateral symmetry and evidence of segmented limbs within the traces (*157, 160, 161*). Dated at 528.82 Ma, these traces are slightly younger than the scalidophores from the Kuanchuanpu Formation (*162*).

The Lantian Biota also has extensive macrofossils without any definitive metazoans, but it is older than the Weng'an Biota (*17*), making the Weng'an a more reasonable choice.

Node: Cryptovermes: Nematoda-Arthropoda | 590.8-528.82 Ma

Node calibrated: Last common ancestor of the clade encompassing Arthropoda, Onychophora and Nematoda.

Fossil taxon: *Rusophycus* (GSC85983, Geological Survey of Canada) trace fossils (*160, 161*).

Minimum age: Records of *Rusophycus* begin in Member 2 of the Chapel Island Formation of the southwestern Burin Peninsula of southeastern Newfoundland, which defines the base of the *Rusophycus avalonensis* biozone. This can be dated through U-Pb series to 530.02 Ma ± 1.2 Myr, yielding a minimum age of 528.82 Ma (*72, 162, 163*).

Minimum justification: *Rusophycus* trace fossils are widely accepted to have been created by arthropod-grade organisms on the basis of the perceived bilateral symmetry and evidence of segmented limbs within the traces (*157, 160, 161*).

Maximum age: Weng'an Biota, 590.8 Ma (*17*). Yang *et al.* (*17*) date the biota based on Re-Os data to 587.2 Ma ±3.6 Myr, yielding a maximum age constraint of 590.8 Ma.

Maximum justification: The Weng'an Biota yields exceptionally preserved macrofossils of algae but nothing that can definitively be placed into crown Metazoa. Embryo-like fossils preserved here have been suggested to be total group metazoans (*99*), but this is still uncertain, and others have suggested they are non-metazoan holozoans (*11*).

Discussion: The Lantian Biota also has extensive macrofossils without any definitive metazoans, but it is older than the Weng'an Biota (*17*), making the Weng'an a more reasonable choice.

Node: Crown Lobopodia | 590.8 - 528.82 Ma

Our minimum constraint follows (*157*)

Node calibrated: Last common ancestor of the clade encompassing Onychophora and Arthropoda.

Fossil taxon: *Rusophycus* (GSC85983, Geological Survey of Canada) trace fossils (*160, 161*).

Minimum age: Records of *Rusophycus* begin in Member 2 of the Chapel Island Formation of the southwestern Burin Peninsula of southeastern Newfoundland, which defines the base of the *Rusophycus avalonensis* biozone. This can be dated through U-Pb series to 530.02 Ma ± 1.2 Myr, yielding a minimum age of 528.82 Ma (*72, 162, 163*).

Minimum justification: *Rusophycus* trace fossils are widely accepted to have been created by arthropod-grade organisms on the basis of the perceived bilateral symmetry and evidence of segmented limbs within the traces (*157, 160, 161*).

Maximum age: Weng'an Biota, 590.8 Ma (*17*). Yang *et al.* (*17*) date the biota based on Re-Os data to 587.2 Ma ±3.6 Myr, yielding a maximum age constraint of 590.8 Ma.

Maximum justification: The Weng'an Biota yields exceptionally preserved macrofossils of algae but nothing that can definitively be placed into crown Metazoa. Embryo-like fossils preserved here have been suggested to be total group metazoans (*99*), but this is still uncertain, and others have suggested they are non-metazoan holozoans (*11*).

Discussion: The Lantian Biota also has extensive macrofossils without any definitive metazoans, but it is older than the Weng'an Biota (*17*), making the Weng'an a more reasonable choice.

Node: Crown Euarthropoda | 590.8 - 514 Ma

Our minimum constraint follows (*160*).

Node calibrated: Last common ancestor of the clade encompassing Chelicerata, Myriapoda, Crustacea and Hexapoda.

Fossil taxon: *Yicaris dianensis* (YKLP10840, Yunnan Key Laboratory for Palaeobiology, Yunnan University, China) and *Wujicaris muellerei* (YKLP11951) (*164, 165*).

Minimum age: 514 Ma Yu'anshan Formation, Xiaotan section, Yongshan, Yunnan Province (*160, 164*).

Minimum justification: Both *Yicaris* and *Wujicaris* have limb characteristics that place them within crown group Crustacea, and phylogenetic and morphological analyses have confirmed this placement (*160, 164*).

Maximum age: Weng'an Biota, 590.8 Ma (*17*). Yang *et al.* (*17*) date the biota based on Re-Os data to 587.2 Ma ±3.6 Myr, yielding a maximum age constraint of 590.8 Ma.

Maximum justification: The Weng'an Biota yields exceptionally preserved macrofossils of algae but nothing that can definitively be placed into crown Metazoa. Embryo-like fossils preserved here have been suggested to be total group metazoans (*99*), but this is still uncertain, and others have suggested they are non-metazoan holozoans (*11*).

Discussion: The Lantian Biota also has extensive macrofossils without any definitive metazoans, but it is older than the Weng'an Biota (*17*), making the Weng'an a more reasonable choice.

Node: Crown Mandibulata | 531.22 - 514 Ma

Our minimum and maximum constraints follow (*160*).

Node calibrated: Last common ancestor of the clade encompassing Myriapoda, Crustacea and Hexapoda.

Fossil taxon: *Yicaris dianensis* (YKLP10840, Yunnan Key Laboratory for Palaeobiology, Yunnan University, China) and *Wujicaris muellerei* (YKLP11951) (*164, 165*).

Minimum age: 514 Ma Yu'anshan Formation, Xiaotan section, Yongshan, Yunnan Province (*160, 164*).

Minimum justification: Both *Yicaris* and *Wujicaris* have limb characteristics that place them within crown group Crustacea, and phylogenetic and morphological analyses have confirmed this placement (*160, 164*).

Maximum age: Records of *Rusophycus* begin in Member 2 of the Chapel Island Formation of the southwestern Burin Peninsula of southeastern Newfoundland, which defines the base of the *Rusophycus avalonensis* biozone. This can be dated through U-Pb series to 530.02 Ma ± 1.2 Myr, yielding a maximum age of 531.22 Ma (*72, 162, 163*)

Maximum justification: *Rusophycus* trace fossils are widely accepted to have been created by arthropod-grade organisms on the basis of the perceived bilateral symmetry and evidence of segmented limbs within the traces (*157, 160, 161*). However, despite the presence of arthropods at this time, there is no evidence of mandibulates, providing a maximum calibration.

Discussion: The Weng'an Biota yields exceptionally preserved macrofossils of algae but nothing that can definitively be placed into crown Metazoa. Embryo-like fossils preserved here have been suggested to be total group metazoans (*99*), but this is still uncertain, and others have suggested they are non-metazoan holozoans (*11*). However, this is older than *Rusophycus* trace fossils.

Node: Crown Pancrustacea | 531.22 - 514 Ma

Our minimum and maximum constraints follow (*160*)

Node calibrated: Last common ancestor of the clade encompassing Myriapoda, Crustacea and Hexapoda.

Fossil taxon: *Yicaris dianensis* (YKLP10840, Yunnan Key Laboratory for Palaeobiology, Yunnan University, China) and *Wujicaris muellerei* (YKLP11951) (*164, 165*).

Minimum age: 514 Ma Yu'anshan Formation, Xiaotan section, Yongshan, Yunnan Province (*160, 164*).

Minimum justification: Both *Yicaris* and *Wujicaris* have limb characteristics that place them within crown group Crustacea, and phylogenetic and morphological analyses have confirmed this placement (*160, 164*).

Maximum age: Records of *Rusophycus* begin in Member 2 of the Chapel Island Formation of the southwestern Burin Peninsula of southeastern Newfoundland, which defines the base of the *Rusophycus avalonensis* biozone. This can be dated through U-Pb series to 530.02 Ma ± 1.2 Myr, yielding a maximum age of 531.22 Ma (*72, 162, 163*).

Maximum justification: *Rusophycus* trace fossils are widely accepted to have been created by arthropod-grade organisms on the basis of the perceived bilateral symmetry and evidence of segmented limbs within the traces (*157, 160, 161*). However, despite the presence of arthropods at this time, there is no evidence of pancrustaceans, providing a maximum calibration.

Discussion: The Weng'an Biota yields exceptionally preserved macrofossils of algae but nothing that can definitively be placed into crown Metazoa. Embryo-like fossils preserved here have been suggested to be total group metazoans (*99*), but this is still uncertain, and others have suggested they are non-metazoan holozoans (*11*). However, this is older than *Rusophycus* trace fossils.

Node: Copepoda-Branchiopoda | 531.22 - 497 Ma

Our minimum and maximum constraints follow (*160*).

Node calibrated: Last common ancestor of the clade encompassing copepods and branchiopods.

Fossil taxon: *Rehbachiella* (UB644, University of Bonn, Germany) (*166*).

Minimum age: *Rehbachiella* is an Orsten fossil, which come from the lowest zone of the late Cambrian Alum Shale. This has been dated to 499 Ma ± 2 Myr, yielding a minimum age of 497 Ma (*160, 167*).

Minimum justification: *Rehbachiella* is well described as a branchiopod, preserved as a complete larva and extensively described (*166*). The Orsten lagerstatte yields very well-preserved fossils.

Maximum age Records of *Rusophycus* begin in Member 2 of the Chapel Island Formation of the southwestern Burin Peninsula of southeastern Newfoundland, which defines the base of the *Rusophycus avalonensis* biozone. This can be dated through U-Pb series to 530.02 Ma ± 1.2 Myr, yielding a maximum age of 531.22 Ma (*72, 162, 163*).

Maximum justification: *Rusophycus* trace fossils are widely accepted to have been created by arthropod-grade organisms on the basis of the perceived bilateral symmetry and evidence of segmented limbs within the traces (*157, 160, 161*). However, despite the presence of arthropods at this time, there is no evidence of copepods, providing a maximum calibration.

Discussion: The Weng'an Biota yields exceptionally preserved macrofossils of algae but nothing that can definitively be placed into crown Metazoa. Embryo-like fossils preserved here have been suggested to be total group metazoans (*99*), but this is still uncertain, and others have suggested they are non-metazoan holozoans (*11*). However, this is older than *Rusophycus* trace fossils.

Node: Crown Hexapoda | 514 - 405 Ma

Our minimum and maximum constraints follow (*160*)

Node calibrated: Last common ancestor of the clade encompassing Insecta and Entognatha.

Fossil taxon: *Rhyniella praecursor* (NHMUKIN.27765, Natural History Museum, London, UK) (*160, 59*).

Minimum age: *Rhyniella* is from the Early Devonian Rhynie Chert, Scotland. The Rhynie Chert has been dated to the Pragian to Emsian, and radiometric dating suggested a maximum age of 411 Ma. The Pragian-Emsian boundary can be used as the age of the Rhynie Chert, yielding an age of 407.6 Ma ± 2.6 Myr, or 405 Ma (*160*).

Minimum justification: Originally interpreted as an insect (within crown Hexapoda), *Rhyniella* was then re-interpreted as a collembolan within the extant entomobryomorph family Isotomidae (*59*).

Maximum age: The Emu Bay Shale of Kangaroo Island, South Australia has been correlated based on trilobite biostratigraphy to the upper part of the *P. janeae* Zone in mainland South Australia, which is correlated to the Canglangpuan Stage in South China and the late Botoman in Siberia. It can therefore by dated to the Cambrian Series 2, Stage 4, and has a maximum age of 514 Ma (*160*).

Maximum justification: The maximum comes from the oldest chelicerate, *Wisangocaris barbarahardyae* from the Emu Bay Shale (*160*). The preservation of a chelicerate suggests exceptional preservation at the site, but no crown hexapods have been found.

Discussion: The Weng'an Biota yields exceptionally preserved macrofossils of algae but nothing that can definitively be placed into crown Metazoa. Embryo-like fossils preserved here have been suggested to be total group metazoans (*99*), but this is still uncertain, and others have suggested they are non-metazoan holozoans (*11*). However, this is older than the Emu Bay Shale.

Node: Crown Insecta | 514 - 405 Ma

Our minimum and maximum constraints follow (*160*)

Node calibrated: Insecta (Orthoptera, Hemiptera, Coleoptera)

Fossil taxon: *Rhyniognatha hirsti* (BMNHIN.38234, Natural History Museum, London, UK) (*169*).

Minimum age: *Rhyniognatha* is from the Early Devonian Rhynie Chert, Scotland. The Rhynie Chert has been dated to the Pragian to Emsian, and radiometric dating suggested a maximum age of 411 Ma. The Pragian-Emsian boundary can be used as the age of the Rhynie Chert, yielding an age of 407.6 Ma ± 2.6 Myr, or 405 Ma (*160*).

Minimum justification: *Rhynioognatha* has preserved articulated mandibles that are diagnostic of crown Insecta (*160, 169*).

Maximum age: The Emu Bay Shale of Kangaroo Island, South Australia has been correlated based on trilobite biostratigraphy to the upper part of the *P. janeae* Zone in mainland South Australia, which is correlated to the Canglangpuan Stage in South China and the late Botoman in Siberia. It can therefore by dated to the Cambrian Series 2, Stage 4, and has a maximum age of 514 Ma (*160*).

Maximum justification: The maximum comes from the oldest chelicerate, *Wisangocaris barbarahardyae* from the Emu Bay Shale (*160*). The preservation of a chelicerate suggests exceptional preservation at the site, but no crown insects have been found.

Discussion: The Weng'an Biota yields exceptionally preserved macrofossils of algae but nothing that can definitively be placed into crown Metazoa. Embryo-like fossils preserved here have been suggested to be total group metazoans (*99*), but this is still uncertain, and others have suggested they are non-metazoan holozoans (*11*). However, this is older than the Emu Bay Shale.

Node: Crown Eumetabola | 411 - 319.9 Ma

Our minimum and maximum constraints follow (*160*).

Node calibrated: Eumetabola (Hemiptera and Coleoptera).

Fossil taxon: *Protoprosbole straeleni* (IRNSBa9885, Institut Royal Des Sciences Naturelles de Belgique, Brussels, Belgium) (*170*).

Minimum age: *Protoprosbole* is found in the Charbonnage de Monceau-Fontaine, Charleroi Coal Basin of Belgium, likely in the latest Marsdenian strata in the *Bilinguites superbilinguis* R2c2 subzone of goniatite ammonoid stratigraphy, yielding an age of ~319.9 Ma (*160, 171*).

Minimum justification: Although the position of *Protoprosbole* has been debated, conservatively it can be described as a stem group Condylognatha (thus crown Eumetabola and Neoptera) due to its morphology, including wing venation (*170*).

Maximum age: The Rhynie Chert has been dated to the Pragian to Emsian, and radiometric dating suggested a maximum age of 411 Ma (*160*).

Maximum justification: The hexapod *Rhyniella praecursor* from the Rhynie Chert (411 Ma) serves as the maximum age calibration. The Rhynie Chert preserves many species including this hexapod, but nothing that can be placed within crown Eumetabola.

Node: Pycnogonida-other Chelicerates (Crown Chelicerata) | 590.8 - 509 Ma

Our minimum constraint follows (*160*).

Node calibrated: Last common ancestor of the clade encompassing Pycnogonida (sea spiders) and Euchelicerata.

Fossil taxon: *Wisangocaris barbarahardyae* (SAMP45427, South Australian Museum, Adelaide, Australia), a mid-Cambrian fossil from Emu Bay Shale, south Australia.

Minimum age: The Emu Bay Shale of Kangaroo Island, South Australia has been correlated based on trilobite biostratigraphy to the upper part of the *P. janeae* Zone in mainland South Australia, which is correlated to the Canglangpuan Stage in South China and the late Botoman in Siberia. It can therefore by dated to the Cambrian Series 2, Stage 4, and has a minimum age of 509 Ma (*160, 172*).

Minimum justification: *Wisangocaris barbarahardyae* has been resolved as more closely related to Euchelicerata than to Pantopoda through phylogenetic and morphological studies, placing it within crown Chelicerata (*160, 172*).

Maximum age: Weng'an Biota, 590.8 Ma (*17*). Yang *et al.* (*17*) date the biota based on Re-Os data to 587.2 Ma ±3.6 Myr, yielding a maximum age constraint of 590.8 Ma.

Maximum justification: The Weng'an Biota yields exceptionally preserved macrofossils of algae but nothing that can definitively be placed into crown Metazoa. Embryo-like fossils preserved here have been suggested to be total group metazoans (*99*), but this is still uncertain, and others have suggested they are non-metazoan holozoans (*11*).

Discussion: The Lantian Biota also has extensive macrofossils without any definitive metazoans, but it is older than the Weng'an Biota (*17*), making the Weng'an a more reasonable choice.

Node: Acari- Araneae | 590.8 - 435.15 Ma

Our minimum constraint follows (*160*).

Node calibrated: Last common ancestor of the clade encompassing Acariformes and Araneae (Arachnida).

Fossil taxon: *Palaeophonus loudonensis* (NMS1897.122.196, National Museum of Scotland, Edinburgh, Scotland).

Minimum age: *Palaeophonus* is found in the Gutterford Burn section of the "Eurypterid Bed" (Reservoir Formation) of the Pentland Hills, Midlothian, Scotland, which has been dated to the Llandovery based on graptolite fauna (the *Oktavites spiralis* Biozone). The biozone has a minimum age of 435.15 Ma (*160*).

Minimum justification: *Palaeophonus* has been included in phylogenetic analyses which resolved it as total-group Scorpiones, meaning it is crown-Arachnida (*160, 173*).

Maximum age: Weng'an Biota, 590.8 Ma (*17*). Yang *et al.* (*17*) date the biota based on Re-Os data to 587.2 Ma ±3.6 Myr, yielding a maximum age constraint of 590.8 Ma.

Maximum justification: The Weng'an Biota yields exceptionally preserved macrofossils of algae but nothing that can definitively be placed into crown Metazoa. Embryo-like fossils preserved here have been suggested to be total group metazoans (*99*), but this is still uncertain, and others have suggested they are non-metazoan holozoans (*11*).

Discussion: The Lantian Biota also has extensive macrofossils without any definitive metazoans, but it is older than the Weng'an Biota (*17*), making the Weng'an a more reasonable choice.

Node: Crown Deuterostomia | 590.8 - 517.32 Ma

Node calibrated: Last common ancestor of the clade encompassing chordates, echinoderms and hemichordates (when monophyletic)

Fossil taxon: Chengjiang fossils such as *Haikouichthyes ercaicunensis* (HZ-f-12-127, Yunnan Institute of Geological Sciences, Kunming, China) (*174*).

Minimum age: The age of the Chengjiang biota has been determined through CA-ID-TIMS U-Pb dating, yielding an age of 518.03 Ma ± 0.69/0.71 Myr and a minimum calibration age of 517.32 Ma (*175*).

Minimum justification: *Haikouichthyes* is widely accepted as at least a crown-chordate and possibly a crown-vertebrate, on the basis of its branchial structures, myomeres, and a notochord (*174, 176*).

Maximum age: Weng'an Biota, 590.8 Ma (*17*). Yang *et al.* (*17*) date the biota based on Re-Os data to 587.2 Ma ±3.6 Myr, yielding a maximum age constraint of 590.8 Ma.

Maximum justification: The Weng'an Biota yields exceptionally preserved macrofossils of algae but nothing that can definitively be placed into crown Metazoa. Embryo-like fossils preserved here have been suggested to be total group metazoans (*99*), but this is still uncertain, and others have suggested they are non-metazoan holozoans (*11*).

Discussion: Isolated pelmatozoan columnals with characteristic morphology and stereom structure of the echinoderm total group have been found, however they are dated to 515.5 Ma and are therefore younger than *Haikouichthyes* (*177, 178*).

The Lantian Biota also has extensive macrofossils without any definitive metazoans, but it is older than the Weng'an Biota (*17*), making the Weng'an a more reasonable choice.

Node: Crown Chordata | 590.8 - 517.32 Ma

Node calibrated: Last common ancestor of the clade encompassing vertebrates, tunicates, and cephalochordates.

Fossil taxon: Chengjiang fossils such as *Haikouichthyes ercaicunensis* (HZ-f-12-127, Yunnan Institute of Geological Sciences, Kunming, China) (*174*).

Minimum age: The age of the Chengjiang biota has been determined through CA-ID-TIMS U-Pb dating, yielding an age of 518.03 Ma ± 0.69/0.71 Myr and a minimum calibration age of 517.32 Ma (*175*).

Minimum justification: *Haikouichthyes* is widely accepted as at least a crown-chordate and possibly a crown-vertebrate, on the basis of its branchial structures, myomeres, and a notochord (*174, 176*).

Maximum age: Weng'an Biota, 590.8 Ma (*17*). Yang *et al.* (*17*) date the biota based on Re-Os data to 587.2 Ma ±3.6 Myr, yielding a maximum age constraint of 590.8 Ma.

Maximum justification: The Weng'an Biota yields exceptionally preserved macrofossils of algae but nothing that can definitively be placed into crown Metazoa. Embryo-like fossils preserved here have been suggested to be total group metazoans (*99*), but this is still uncertain, and others have suggested they are non-metazoan holozoans (*11*).

Discussion: The Lantian Biota also has extensive macrofossils without any definitive metazoans, but it is older than the Weng'an Biota (*17*), making the Weng'an a more reasonable choice.

Node: Crown Olfactores | 590.8 - 517.32 Ma

Node calibrated: Last common ancestor of the clade encompassing tunicates and Vertebrata-Craniata.

Fossil taxon: Chengjiang fossils such as *Haikouichthyes ercaicunensis* (HZ-f-12-127, Yunnan Institute of Geological Sciences, Kunming, China) (*174*).

Minimum age: The age of the Chengjiang biota has been determined through CA-ID-TIMS U-Pb dating, yielding an age of 518.03 Ma ± 0.69/0.71 Myr and a minimum calibration age of 517.32 Ma (*175*).

Minimum justification: *Haikouichthyes* is widely accepted as at least a crown-chordate and possibly a crown-vertebrate, on the basis of its branchial structures, myomeres, and a notochord (*174, 176*).

Maximum age: Weng'an Biota, 590.8 Ma (*17*). Yang *et al.* (*17*) date the biota based on Re-Os data to 587.2 Ma ±3.6 Myr, yielding a maximum age constraint of 590.8 Ma.

Maximum justification: The Weng'an Biota yields exceptionally preserved macrofossils of algae but nothing that can definitively be placed into crown Metazoa. Embryo-like fossils preserved here have been suggested to be total group metazoans (*99*), but this is still uncertain, and others have suggested they are non-metazoan holozoans (*11*).

Discussion: The Lantian Biota also has extensive macrofossils without any definitive metazoans, but it is older than the Weng'an Biota (*17*), making the Weng'an a more reasonable choice.

Node: Crown Vertebrata | 590.8 - 497 Ma

Node calibrated: Last common ancestor of the clade encompassing Cyclostomata and Gnathostomata.

Fossil taxon: *Furnishina begminata* (GMPKU2589; Geological Museum, Peking University, Beijing, China).

Minimum age: *Furnishina* is found in the middle Cambrian Huaqiao Formation at Wangcun Section, northern Hunan Province, China, dated to 497 Ma (*83*).

Minimum justification: Conodonts are tooth-like 'elements' that have been interpreted as coming from vertebrates, possibly stem gnathostomes (*179*). Soft tissue fossils show notochords, segmental trunk musculature, a caudal fin, paired sensory organs, and gill pouches, all of which support a vertebrate affinity. The oldest conodont is *Furnishina*, a paraconodont in the euconodont lineage (*180*).

Maximum age: Weng'an Biota, 590.8 Ma (*17*). Yang *et al.* (*17*) date the biota based on Re-Os data to 587.2 Ma ±3.6 Myr, yielding a maximum age constraint of 590.8 Ma.

Maximum justification: The Weng'an Biota yields exceptionally preserved macrofossils of algae but nothing that can definitively be placed into crown Metazoa. Embryo-like fossils preserved

here have been suggested to be total group metazoans (*99*), but this is still uncertain, and others have suggested they are non-metazoan holozoans (*11*).

Discussion: *Arandaspis priontolepis* is a total-group gnathostome, making it crown-group Vertebrata, however it is dated to 457.5 Ma and is therefore younger than *Furnishina* (*157*).

The Lantian Biota also has extensive macrofossils without any definitive metazoans, but it is older than the Weng'an Biota (*17*), making the Weng'an a more reasonable choice.

Node: Crown Gnathostomata | 468.4 - 431.7 Ma

Node calibrated: Last common ancestor of the clade encompassing Osteichthyes and Chondrichthyes.

Fossil taxon: *Shenacanthus vermiformis* (V300000; Institute of Vertebrate Palaeontology and Palaeoanthropology) (*181*).

Minimum age: *Shenacanthus* is found in the early Silurian (Telychian) Huixingshao Formation of Chongqing, South China (*181*). This has been established as Llandovery in age, and so a minimum age constraint can be established based on the Llandovery-Wenlock boundary. This has been dated to 432.9 Ma ± 1.2 Myr (*182*), yielding a minimum age of 431.7 Ma.

Minimum justification: *Shenacanthus* is resolved as a derived stem-chondrichthyan in a phylogenetic analysis by (*181*).

Maximum age: Ostracoderm assemblages from the Stairway Sandstone Formation of Mt Watt, Amadeus Basin, Northern Territory, Australia, dated to 467.3 Ma ± 1.1 Myr, yielding a maximum age of 468.4 Ma (*157*).

Maximum justification: Ostracoderms are stem group gnathostomes, and their presence in the Ordovician demonstrates the potential for preservation of gnathostomes. However, no crown representatives have been found.

Discussion: *Guiyu oneiros* has lobe-finned fish synapmorphies which identify it as a stem-sarcopterygian and therefore crown Osteichthyes and crown Gnathostomata, however, it is dated younger than *Shenacanthus* at 420.7 Ma (*157*).

Scales from possible chondrichthyans have also been found from the Stairway Sandstone, but dated slightly older than *Shenacanthus* at 457.7 Ma. However, these are difficult to place phylogenetically and this uncertainty makes them unsuitable for use as calibrations (*157*).

Nerepisacanthus is an acanthodian, making it crown gnathostome and possibly stem chondrichthyan (*183*). However, it is found in the Bertie Formation which is dated to 423 Ma ± 2.3 Myr to 4.19.2 Ma ± 3.2 Myr, making it either the same age or younger than *Guiyu*, and much younger than *Shenacanthus*.

Node: Crown Osteichthyes | 444.9 - 420.7 Ma

Our minimum and maximum constraints follow (*157*)

Node calibrated: Last common ancestor of the clade encompassing Actinopterygii and Sarcopterygii.

Fossil taxon: *Guiyu oneiros* (IVPP V15541, Institute of Vertebrate Paleontology and Paleoanthropology, Beijing)

Minimum age: *Guiyu* is found in the Kuanti Formation, Xiaoxiang Reservoir, Quijing, Yunnan, China. Based on conodont index fossils, the formation has been correlated with the Ludlow-Pridoli boundary , which has been dated to 423 Ma ± 2.3 Myr, yielding a minimum age of 420.7 Ma (*182*).

Minimum justification: *Guiyu* has lobe-finned fish synapomorphies which identify it as a stem sarcopterygian and therefore crown Osteichthyes (*157, 184*)

Maximum age: The base of the Llandovery, dated at 443.4 Ma ± 1.5 Myr, or 444.9 Ma, can be used as the soft maximum calibration (*182*).

Maximum justification: There are records of diverse jawless fishes in the Silurian rocks, but nothing that can be identified as crown Osteichthyes (*157*)

Node: Crown Tetrapoda | 351 - 337 Ma

Our minimum and maximum constraints follow (*157*).

Node calibrated: Last common ancestor of the clade encompassing amphibians, reptiles, birds and mammals.

Fossil taxon: *Lethiscus stocki* (MCZ 2185, Museum of Comparative Zoology, Harvard).

Minimum age: *Lethiscus* has been found in the Wardie Shales of the Wardie shore, near Edinburgh, Scotland. The Wardie Shales have been assigned to the Holkerian regional stage based on fossil fishes and palynomorphs, resulting in an age of 343.5-337 Ma (*185*). This gives a minimum age estimate of 337 Ma.

Minimum justification: *Lethiscus* is either a Lepospondyli (within Batrachomorpha) or a Reptilimorpha, but since both are crown Tetrapoda it can be safely interpreted as a crown tetrapod (*157, 186*).

Maximum age: Whatcheeriids from the Ivorian regional Western European stage are dated to 351-346.5 Ma.

Maximum justification: Whatcheeriids such as *Whatcheeria* and *Pederpes* are stem tetrapods, phylogenetically distant from the crown. They therefore demonstrate adequate fossilisation potential, with no evidence of crown tetrapods found at the same time.

Discussion: Both baphetids and colosteids are nearer to the crown (but still stem) of Tetrapoda, and could therefore be better maximum calibrations. However, they are both younger than *Lethiscus* (*157*).

Node: Crown Amniota | 332.9 - 318 Ma

Our minimum and maximum constraints follow (*157*).

Node calibrated: Last common ancestor of the clade encompassing lizards, turtles, birds and mammals.

Fossil taxon: *Hylonomus lyelli* (NHMUK R4168, Natural History Museum, London).

Minimum age: *Hylonomus* was recovered from division 4, section XV, coal group 15 of the Joggins Formation of Nova Scotia. Although much debated, the current age for the Joggins Formation is estimated to be 319-318 Ma, giving a minimum age of 318 Ma (*157, 185*).

Minimum justification: *Hylonomus lyelli* is a eureptilian (stem diapsid) based on morphological synapomorphies, which makes it a crown amniote (*187*).

Maximum age: The Little Cliff Shale of the East Kirkton locality is assigned to the Brigantian, with a maximum age of 332.9 Ma (*157, 185*)

Maximum justification: The East Kirkton locality yields diverse batrachomorphs and reptiliomorphs, but no diapsids or synapsids, allowing its use as the maximum age calibration (*150*).

Node: Crown Mammalia | 252.2 - 164.1 Ma

Our minimum and maximum constraints follow (*188*).

Node calibrated: Last common ancestor of the clade encompassing monotremes, marsupials and placentals.

Fossil taxon: *Ambondro mahabo* (UA-10602, University of Antananarivo, Madagascar) and other Bathonian australosphenidans (*189*).

Minimum age: *Ambondro* comes from the upper part of the Isalo "Group" of Madagascar, which has been dated to the Bathonian, middle Jurassic (*189*). We therefore use the top of the Bathonian (165.3 Ma ± 1.2 Myr, or 164.1 Ma) as the minimum age calibration (*185, 190*).

Maximum age: The Permian-Triassic boundary, 251.9 Ma ± 0.3 Myr, or 252.2 Ma (*188, 191*).

Minimum justification: *Ambondro* and related are placed within the monotreme clade Australosphenida (*150, 192*).

Maximum justification: The use of the Permian-Triassic boundary allows for the possibility of haramiyids being crown mammals (*188*).

Discussion: Haramiyids have recently been interpreted as crown Mammalia on the basis of similarities in the inner ear structure (*193, 194*); however, not everyone agrees (*195*) and Bayesian phylogenetics tend to find haramiyids outside of crown Mammalia (*196*). The oldest haramiyids are from the Ørsted Dal Formation, with an age of 201.3 Ma ± 0.2 Myr (*197*), and so are encompassed by the soft maximum of the Permian-Triassic boundary.

Node: Crown Euarchontoglires | 162.5 - 65.79 Ma

Our maximum constraint follows (*188*).

Node calibrated: Last common ancestor of the clade encompassing Euarchonta and Glires.

Fossil taxon: *Purgatorius mckeeveri* (UCMP 157977; University of California Museum of Palaeontology, Berkeley, California, USA) from 'Harley's Point' UCMP locality V77087, early Puercan (Pu1) (lowermost Palaeocene) Tullock Member, Fort Union Formation, Garfield County, Montana, USA (*198*).

Minimum age: $^{40}Ar/^{39}Ar$ dating on a tuff within the overlying sediments yielded an age of 65.844 Ma ± 0.033/0.054 Myr, or 65.790 Ma for the minimum age (*198*)

Maximum age: The Oxfordian/top of the Callovian at the Daxigou site of the Tiaojishan Formation, Liaoning Province, dated to 162.5 Ma (*150, 188, 190*).

Minimum justification: Plesiadapids such as *Purgatorius* are widely accepted as a sister group to Primates, nested within crown Euarchontoglires (*150, 199*).

Maximum justification: The divergence of Eutheria from other mammals represented by *Juramaia* is used as the maximum age calibration (*150*).

Node: Crown Cyclostomata | 590.8 - 358.5 Ma

Our minimum constraint follows (*150*).

Node calibrated: Last common ancestor of the clade encompassing hagfishes and lampreys

Fossil taxon: *Priscomyzon riniensis* (AM5750, Albany Museum, Grahamstown, Eastern Cape, South Africa).

Minimum age: The Witpoort Formation (Witteberg Group) at Waterloo Farm, Grahamstown, South Africa has been dated to the Famennian, and so we can use the Devonian-Carboniferous boundary of 358.9 Ma ± 0.4 Myr, or 385.5 Ma (*150, 200*).

Maximum age: Weng'an Biota, 590.8 Ma (*17*). Yang *et al.* (*17*) date the biota based on Re-Os data to 587.2 Ma ±3.6 Myr, yielding a maximum age constraint of 590.8 Ma.

Minimum justification: The unequivocally total-group petromyzontid (and therefore crown-group Cyclosotmata) *Priscomyzon riniensis* is used as the minimum calibration (*150, 201*).

Maximum justification: The Weng'an Biota yields exceptionally preserved macrofossils of algae but nothing that can definitively be placed into crown Metazoa. Embryo-like fossils preserved here have been suggested to be total group metazoans (*99*), but this is still uncertain, and others have suggested they are non-metazoan holozoans (*11*).

Discussion: The Lantian Biota also has extensive macrofossils without any definitive metazoans, but it is older than the Weng'an Biota (*17*), making the Weng'an a more reasonable choice.

Node: Crown Xenambulacraria | 590.8 - 515.5 Ma

Our minimum constraint follows (*157*) for Ambulacraria.

Node calibrated: Last common ancestor of the clade encompassing Hemichordata, Echinodermata and Xenacoelomorpha.

Fossil taxon: Isolated pelmatozoan columnals (BMNH EE6828-EE6846, Natural History Museum, London, UK).

Minimum age: 515.5 Ma, from the Micmacca Breccia, which encompasses the upper part of the *Cephalopyge notabilis* Biozone, the *Ornamentaspis frequens* Biozone and the lower part of the *Kymataspis arenosa* Biozone, which has been dated to the latest Atdabanian of 517.0 Ma ± 1.5 Myr, or 515.5 ma (*157*).

Maximum age: Weng'an Biota, 590.8 Ma (*17*). Yang *et al.* (*17*) date the biota based on Re-Os data to 587.2 Ma ±3.6 Myr, yielding a maximum age constraint of 590.8 Ma.

Minimum justification: The isolated pelmatzoan columnals have characteristic morphology and stereom structure of the Echinodermata total group (*157, 177, 178*).

Maximum justification: The Weng'an Biota yields exceptionally preserved macrofossils of algae but nothing that can definitively be placed into crown Metazoa. Embryo-like fossils preserved here have been suggested to be total group metazoans (*99*), but this is still uncertain, and others have suggested they are non-metazoan holozoans (*11*).

Node: Crown Ambulacraria | 590.8 - 515.5 Ma

Our minimum constraint follows (*157*).

Node calibrated: Last common ancestor of the clade encompassing Hemichordata and Echinodermata.

Fossil taxon: Isolated pelmatozoan columnals (BMNH EE6828-EE6846, Natural History Museum, London, UK).

Minimum age: 515.5 Ma, from the Micmacca Breccia, which encompasses the upper part of the *Cephalopyge notabilis* Biozone, the *Ornamentaspis frequens* Biozone and the lower part of the *Kymataspis arenosa* Biozone, which has been dated to the latest Atdabanian of 517.0 Ma ± 1.5 Myr, or 515.5 ma (*157*).

Maximum age: Weng'an Biota, 590.8 Ma (*17*). Yang *et al.* (*17*) date the biota based on Re-Os data to 587.2 Ma ±3.6 Myr, yielding a maximum age constraint of 590.8 Ma.

Minimum justification: The isolated pelmatzoan columnals have characteristic morphology and stereom structure of the Echinodermata total group (*157, 177, 178*).

Maximum justification: The Weng'an Biota yields exceptionally preserved macrofossils of algae but nothing that can definitively be placed into crown Metazoa. Embryo-like fossils preserved here have been suggested to be total group metazoans (*99*), but this is still uncertain, and others have suggested they are non-metazoan holozoans (*11*).

Node: Crown Echinodermata | 549 - 509 Ma

Our minimum and maximum constraints follow (*157*).

Node calibrated: Last common ancestor of the clade encompassing all living echinoderms.

Fossil taxon: *Stromatocystites walcotti* (USNM 66483, Smithsonian Institute, National Museum of Natural History, Washington, US).

Minimum age: 509 Ma, from the *Olenellus* Beds, Taconian, upper Lower Cambrian, eastern arm of Bonne Bay, western coast of Newfoundland, which has been correlated with Series 2, Stage 4 of the Cambrian and dated to 509 Ma (*157, 202*).

Maximum age: The Nama Group, 549 Ma (*78, 157*).

Minimum justification: *Stromatocysties walcotti* is interpreted as a crown echinoderm based on synapomorphies such as a pentaradial body plan, a water vascular system, and a stereom skeleton (*150, 203*).

Maximum justification: The Nama Group records an open marine community that includes the earliest animal skeletal remains (e.g. *Cloudina*), but no echinoderms (*157*).

Node: Crown Echinozoa | 549 - 449.6 Ma

Node calibrated: Last common ancestor of the clade encompassing echinoids and holothuroids.

Fossil taxon: *Bothriocidaris pahleni* (BMNH E83655, Natural History Museum, London, UK).

Minimum age: The Caradoc (Ordovician), dated to 458.18-449.6 Ma (*204*).

Maximum age: The Nama Group, 549 Ma (*78, 157*).

Minimum justification: The bothriocidarids are a stem group of echinoids, and therefore are crown Echinozoa. They are found in the upper Ordovician Caradoc series (*205*).

Maximum justification: The Nama Group records an open marine community that includes the earliest animal skeletal remains (e.g. *Cloudina*), but no echinoderms or echinozoans (*157*).

Node: Crown Hemichordata | 590.8 - 504.5 Ma

Our minimum constraint follows (*157*).

Node calibrated: Last common ancestor of the clade encompassing pterobranchs and enteropneusts.

Fossil taxon: *Rhabdotubus johansoni* (SMNH Cn 67217, Swedish Museum of Natural History, Stockholm, Sweden).

Minimum age: Cambrian Series 3, Age 5, dated to 504.5 Ma (*72, 157*).

Maximum age: Weng'an Biota, 590.8 Ma (*17*). Yang *et al.* (*17*) date the biota based on Re-Os data to 587.2 Ma ±3.6 Myr, yielding a maximum age constraint of 590.8 Ma.

Minimum justification: *Rhabdotubus* shows cortical banding that demonstrate it is at least total-group, perhaps crown-group, Ptereobranchia, which means it is crown-group Hemichordata (*206*).

Maximum justification: The Weng'an Biota yields exceptionally preserved macrofossils of algae but nothing that can definitively be placed into crown Metazoa. Embryo-like fossils preserved here have been suggested to be total group metazoans (*99*), but this is still uncertain, and others have suggested they are non-metazoan holozoans (*11*).

Discussion: The fossil *Sokoloviina* has been suggested to be a pterobranch (*207*), however, there have been few detailed analyses of this fossil and the specimen is fragmented, making its affinity uncertain.

Node: Crown Cnidaria | 590.8 - 561.1 Ma

Node calibrated: Last common ancestor of the clade encompassing Anthozoa and Medusozoa.

Fossil taxon: *Auroralumina attenboroughii (*GSM 106119) (*59*).

Minimum age: Bed B, Bradgate Formation, Charnian Supergroup, 563 ±1.9 Ma, so 561.1 Ma (*65*). More recently, the U-Pb-derived age of 556.6 Ma ± 6.4 Myr was suggested (*64*), but the associated uncertainty entirely encompasses the original, more precise date, and so we follow the earlier date from Wilby *et al.* (*65*).

Minimum justification: Formal character analysis and phylogenetic tests placed *Auroralumina attenboroughii* as a crown-cnidarian, likely a stem-group medusozoan (*59*).

Maximum age: Weng'an Biota, 590.8 Ma (*17*). Yang *et al.* (*17*) date the biota based on Re-Os data to 587.2 Ma ±3.6 Myr, yielding a maximum age constraint of 590.8 Ma.

Maximum justification: The Weng'an Biota yields exceptionally preserved macrofossils of algae but nothing that can definitively be placed into crown Metazoa. Embryo-like fossils preserved here have been suggested to be total group metazoans (*99*), but this is still uncertain, and others have suggested they are non-metazoan holozoans (*11*).

Discussion: The embryonic and post-embryonic stages of *Olivooides* from the Kuanchuanpu Formation demonstrate that it is a scyphozoan, however it is dated to 531.8 Ma and is therefore younger than *Auroralumina* (*72, 32*).

The Lantian Biota also has extensive macrofossils without any definitive metazoans, but it is older than the Weng'an Biota (*17*), making the Weng'an a more reasonable choice.

Node: Crown Demospongiae | 515 – 590.8 Ma

Node calibrated: Last common ancestor of the clade encompassing Heteroscleromorpha, Keratosa and Verongimorpha.

Fossil taxon: Spicules from the Sirius Passet (*208*).

Minimum age: Sirius Passet Lagerstätte, dated to 515 Ma (*209*)

Minimum justification: Spicules from the Sirius Passet are well preserved and diagnostic of the extant demosponge lineages Haploscleromorpha and/or Heteroscleromorpha. Although their affinity cannot be narrowed down to the exact group, they are at the very least total group Haploscleromorpha/Heteroscleromorpha, making them crown Demospongiae (*208*).

Maximum age: Weng'an Biota, 590.8 Ma (*17*). Yang *et al.* (*17*) date the biota based on Re-Os data to 587.2 Ma ±3.6 Myr, yielding a maximum age constraint of 590.8 Ma.

Maximum justification: The Weng'an Biota yields exceptionally preserved macrofossils of algae but nothing that can definitively be placed into crown Metazoa. Embryo-like fossils preserved here have been suggested to be total group metazoans (*99*), but this is still uncertain, and others have suggested they are non-metazoan holozoans (*11*).

Discussion: Hazeliidae such as *Vauxia gracilenta* from the Burgess Shale are also undisputed crown group demosponges, however they are younger at 505 Ma (*210*). Archaeocyathids from the Cambrian have also been suggested to be demosponges, however this affinity is not certain as no spicules have been identified and some have interpreted them as cnidarian-grade organisms (*211*).

REFERENCES AND NOTES

1. C. Darwin, *On the Origin of Species by Means of Natural Selection, or the Preservation of Favoured Races in the Struggle for Life* (John Murray, 1859).

2. E. C. Turner, Possible poriferan body fossils in early Neoproterozoic microbial reefs. *Nature* **596**, 87–91 (2021).

3. G. D. Love, E. Grosjean, C. Stalvies, D. A. Fike, J. P. Grotzinger, A. S. Bradley, A. E. Kelly, M. Bhatia, W. Meredith, C. E. Snape, S. A. Bowring, D. J. Condon, R. E. Summons, Fossil steroids record the appearance of Demospongiae during the Cryogenian period. *Nature* **457**, 718–721 (2009).

4. J. A. Zumberge, G. D. Love, P. Cardenas, E. A. Sperling, S. Gunasekera, M. Rohrssen, E. Grosjean, J. P. Grotzinger, R. E. Summons, Demosponge steroid biomarker 26-methylstigmastane provides evidence for Neoproterozoic animals. *Nat. Ecol. Evol.* **2**, 1709–1714 (2018).

5. J. B. Antcliffe, Questioning the evidence of organic compounds called sponge biomarkers. *Palaeontology* **56**, 917–925 (2013).

6. M. O. Brown, B. O. Olagunju, J. L. Giner, P. V. Welander, Sterol methyltransferases in uncultured bacteria complicate eukaryotic biomarker interpretations. *Nat. Commun.* **14**, 1859 (2023).

7. I. Bobrovskiy, J. M. Hope, B. J. Nettersheim, J. K. Volkman, C. Hallmann, J. J. Brocks, Algal origin of sponge sterane biomarkers negates the oldest evidence for animals in the rock record. *Nat. Ecol. Evol.* **5**, 165–168 (2021).

8. B. J. Nettersheim, J. J. Brocks, A. Schwelm, J. M. Hope, F. Not, M. Lomas, C. Schmidt, R. Schiebel, E. C. M. Nowack, P. De Deckker, J. Pawlowski, S. S. Bowser, I. Bobrovskiy, K. Zonneveld, M. Kucera, M. Stuhr, C. Hallmann, Putative sponge biomarkers in unicellular Rhizaria question an early rise of animals. *Nat. Ecol. Evol.* **3**, 577–581 (2019).

9. G. D. Love, J. A. Zumberge, P. Cardenas, E. A. Sperling, M. Rohrssen, E. Grosjean, J. P. Grotzinger, R. E. Summons, Sources of C(30) steroid biomarkers in Neoproterozoic-Cambrian rocks and oils. *Nat. Ecol. Evol.* **4**, 34–36 (2020).

10. Z. J. Yin, W. C. Sun, P. J. Liu, J. Y. Chen, D. J. Bottjer, J. H. Li, M. Y. Zhu, Diverse and complex developmental mechanisms of early Ediacaran embryo-like fossils from the Weng'an biota, southwest China. *Philos. Trans. R. Soc. B Biol. Sci.* **377**, 12 (2022).

11. T. Huldtgren, J. A. Cunningham, C. Yin, M. Stampanoni, F. Marone, P. C. J. Donoghue, S. Bengtson, Fossilized nuclei and germination structures identify ediacaran “animal embryos” as encysting protists. *Science* **334**, 1696–1699 (2011).

12. T. Huldtgren, J. A. Cunningham, C. Y. Yin, M. Stampanoni, F. Marone, P. C. J. Donoghue, S. Bengtson, Response to comment on “fossilized nuclei and germination structures identify ediacaran 'animal embryos' as encysting protists”. *Science* **335**, 1169 (2012).

13. L. Chen, S. Xiao, K. Pang, C. M. Zhou, X. L. Yuan, Cell differentiation and germ-soma separation in Ediacaran animal embryo-like fossils. *Nature* **516**, 238–241 (2014).

14. Z. Yin, K. Vargas, J. Cunningham, S. Bengtson, M. Y. Zhu, F. Marone, P. Donoghue, The Early Ediacaran *Caveasphaera* foreshadows the evolutionary origin of animal-like embryology. *Curr. Biol.* **29**, 4307–4314.e2 (2019).

15. Z. Yin, W. Sun, P. Liu, M. Zhu, P. C. J. Donoghue, Developmental biology of *Helicoforamina* reveals holozoan affinity, cryptic diversity, and adaptation to heterogeneous environments in the early Ediacaran Weng'an biota (Doushantuo Formation, South China). *Sci. Adv.* **6**, eabb0083 (2020).

16. F. Tang, S. Bengtson, Y. Wang, X.-L. Wang, C.-Y. Yin, Eoandromeda and the origin of Ctenophora. *Evol. Dev.* **13**, 408–414 (2011).

17. C. Yang, A. D. Rooney, D. J. Condon, X.-H. Li, D. V. Grazhdankin, F. T. Bowyer, C. Hu, F. A. Macdonald, M. Y. Zhu, The tempo of Ediacaran evolution. *Sci. Adv.* **7**, eabi9643 (2021).

18. F. Tang, C. Yin, S. Bengtson, P. Liu, Z. Wang, L. Gao, Octoradiate spiral organisms in the Ediacaran of South China. *ACTA. GEOL. SIN-ENGL.* **82**, 27–34 (2008).

19. M. Zhu, J. G. Gehling, S. Xia, Y. Zhao, M. L. Droser, Eight-armed Ediacara fossil preserved in contrasting taphonomic windows from China and Australia. *Geology* **36**, 867–870 (2008).

20. Y. Zhao, J. Vinther, L. A. Parry, F. Wei, E. Green, D. Pisani, X. Hou, G. D. Edgecombe, P. Cong, Cambrian sessile, suspension feeding stem-group ctenophores and evolution of the comb jelly body plan. *Curr. Biol.* **29**, 1112–1125.e2 (2019).

21. A. G. Liu, D. McIlroy, M. D. Brasier, First evidence for locomotion in the Ediacara biota from the 565 Ma Mistaken Point Formation, Newfoundland. *Geology* **38**, 123–126 (2010).

22. F. S. Dunn, A. G. Liu, D. V. Grazhdankin, P. Vixseboxse, J. Flannery-Sutherland, E. Green, S. Harris, P. R. Wilby, P. C. J. Donoghue, The developmental biology of *Charnia* and the eumetazoan affinity of the Ediacaran rangeomorphs. *Sci. Adv.* **7**, eabe0291 (2021).

23. F. T. Bowyer, A. Y. Zhuravlev, R. Wood, G. A. Shields, Y. Zhou, A. Curtis, S. W. Poulton, D. J. Condon, C. Yang, M. Zhu, Calibrating the temporal and spatial dynamics of the Ediacaran - Cambrian radiation of animals. *Earth Sci. Rev.* **225**, 103913 (2021).

24. M. W. Martin, D. V. Grazhdankin, S. A. Bowring, D. A. D. Evans, M. A. Fedonkin, J. L. Kirschvink, Age of Neoproterozoic bilatarian body and trace fossils, White Sea, Russia: Implications for metazoan evolution. *Science* **288**, 841–845 (2000).

25. B. Duan, X.-P. Dong, L. Porras, K. Vargas, J. A. Cunningham, P. C. J. Donoghue, The early Cambrian fossil embryo *Pseudooides* is a direct-developing cnidarian, not an early ecdysozoan. *Proc. Biol. Sci.* **284**, 20172188 (2017).

26. H. Zhang, S. Xiao, Y. Liu, X. Yuan, B. Wan, A. D. Muscente, T. Shao, H. Gong, G. Cao, Armored kinorhynch-like scalidophoran animals from the early Cambrian. *Sci. Rep.* **5**, 16521 (2015).

27. Y. Liu, S. Xiao, T. Shao, J. Broce, H. Zhang, The oldest known priapulid-like scalidophoran animal and its implications for the early evolution of cycloneuralians and ecdysozoans. *Evol. Dev.* **16**, 155–165 (2014).

28. T.-Q. Shao, Y.-H. Liu, Q. Wang, H.-Q. Zhang, H.-H. Tang, Y. Li, New material of the oldest known scalidophoran animal *Eopriapulites* sphinx. *Palaeoworld* **25**, 1–11 (2016).

29. D. Wang, J. Vannier, I. Schumann, X. Wang, X. G. Yang, T. Komiya, K. Uesugi, J. Sun, J. Han, Origin of ecdysis: Fossil evidence from 535-million-year-old scalidophoran worms. *Proc. Biol. Sci.* **286**, 20190791 (2019).

30. H. Zhang, A. Maas, D. Waloszek, New material of scalidophoran worms in Orsten-type preservation from the Cambrian Fortunian Stage of South China. *J. Paleo.* **92**, 14–25 (2018).

31. M. Steiner, G. Li, Y. Qian, M. Zhu, B.-D. Erdtmann, Neoproterozoic to early Cambrian small shelly fossil assemblages and a revised biostratigraphic correlation of the Yangtze Platform (China). *Palaeogeogr. Palaeoclimatol. Palaeoecol.* **254**, 67–99 (2007).

32. X.-P. Dong, J. A. Cunningham, S. Bengtson, C.-W. Thomas, J. Liu, M. Stampanoni, P. C. J. Donoghue, Embryos, polyps and medusae of the Early Cambrian scyphozoan *Olivooides*. *Proc. Biol. Sci.* **280**, 20130071 (2013).

33. J. Y. Chen, J. W. Schopf, D. J. Bottjer, C. Y. Zhang, A. B. Kudryavtsev, A. B. Tripathi, X. Q. Wang, Y. H. Yang, X. Gao, Y. Yang, Raman spectra of a Lower Cambrian ctenophore embryo from southwestern Shaanxi, China. *Proc. Natl. Acad. Sci. U.S.A.* **104**, 6289–6292 (2007).

34. R. A. Fortey, D. E. G. Briggs, M. A. Wills, The Cambrian evolutionary 'explosion': Decoupling cladogenesis from morphological disparity. *Biol. J. Linn. Soc.* **57**, 13–33 (1996).

35. D. A. T. Harper, E. U. Hammarlund, T. P. Topper, A. T. Nielsen, J. A. Rasmussen, T.-Y. S. Park, M. P. Smith, The Sirius Passet Lagerstatte of North Greenland: A remote window on the Cambrian explosion. *J. Geol. Soc. London* **176**, 1023–1037 (2019).

36. X. Hou, D. J. Siveter, D. J. Siveter, R. J. Aldridge, P.-y. Cong, S. E. Gabbott, X.-y. Ma, M. A. Purnell, M. Williams, *The Cambrian Fossils of Chengjiang, China: The Flowering of Early Animal Life* (John Wiley & Sons Inc., 2017).

37. D. E. G. Briggs, D. H. Erwin, F. J. Collier, L. McKnight, *The Fossils of the Burgess Shale* (Smithsonian Institution Press, 1994).

38. G. E. Budd, R. P. Mann, Survival and selection biases in early animal evolution and a source of systematic overestimation in molecular clocks. *Interface Focus* **10**, 20190110 (2020).

39. B. Runnegar, The Cambrian explosion: Animals or fossils? *J. Geol. Soc. Aust.* **29**, 395–411 (1982).

40. S. E. Peters, J. M. Husson, We need a global comprehensive stratigraphic database: Here's a start. *The Sedimentary Rec.* **16**, 4–9 (2018).

41. D. C. Segessenman, S. E. Peters, Transgression–regression cycles drive correlations in Ediacaran–Cambrian rock and fossil records. *Paleobiology* **50**, 150–163 (2024).

42. J. A. Cunningham, A. G. Liu, S. Bengtson, P. C. J. Donoghue, The origin of animals: Can molecular clocks and the fossil record be reconciled? *Bioessays* **39**, 1–12 (2017).

43. D. H. Erwin, M. Laflamme, S. M. Tweedt, E. A. Sperling, D. Pisani, K. J. Peterson, The Cambrian conundrum: Early divergence and later ecological success in the early history of animals. *Science* **334**, 1091–1097 (2011).

44. M. dos Reis, Y. Thawornwattana, K. Angelis, M. J. Telford, P. C. J. Donoghue, Z. H. Yang, Uncertainty in the timing of origin of animals and the limits of precision in molecular timescales. *Curr. Biol.* **25**, 2939–2950 (2015).

45. M. Dohrmann, G. Wörheide, Dating early animal evolution using phylogenomic data. *Sci. Rep.* **7**, 3599 (2017).

46. A. C. Daley, J. B. Antcliffe, H. B. Drage, S. Pates, Early fossil record of Euarthropoda and the Cambrian explosion. *Proc. Natl. Acad. Sci. U.S.A.* **115**, 5323–5331 (2018).

47. A. J. S. Beavan, D. Pisani, P. C. J. Donoghue, Diversification dynamics of total-, stem-, and crown-groups are compatible with molecular clock estimates of divergence times. *Sci. Adv.* **7**, eabf2257 (2021).

48. G. E. Budd, R. P. Mann, Two notorious nodes: A critical examination of relaxed molecular clock age estimates of the bilaterian animals and placental mammals. *Syst. Biol.* **73**, 223–234 (2024).

49. P. C. J. Donoghue, Z. Yang, The evolution of methods for establishing evolutionary timescales. *Philos. Trans. R. Soc. Lond. B Biol. Sci.* **371**, 20160020 (2016).

50. S. M. Holland, The non-uniformity of fossil preservation. *Philos. Trans. R. Soc. Lond. B Biol. Sci.* **371**, 20150130 (2016).

51. M. Dos Reis, Z. Yang, The unbearable uncertainty of Bayesian divergence time estimation. *J. Syst. Evol.* **51**, 30–43 (2013).

52. P. C. J. Donoghue, M. J. Benton, Rocks and clocks: Calibrating the tree of life using fossils and molecules. *Trends Ecol. Evol.* **22**, 424–431 (2007).

53. G. E. Budd, R. P. Mann, The dynamics of stem and crown groups. *Sci. Adv.* **6**, eaaz1626 (2020).

54. X. L. Yuan, Z. Chen, S. H. Xiao, B. Wan, C. G. Guan, W. Wang, C. M. Zhou, H. Hua, The Lantian biota: A new window onto the origin and early evolution of multicellular organisms. *Chin. Sci. Bull.* **58**, 701–707 (2013).

55. C. Yang, Y. Li, D. Selby, B. Wan, C. Guan, C. Zhou, X.-H. Li, Implications for Ediacaran biological evolution from the ca. 602 Ma Lantian biota in China. *Geology* **50**, 562–566 (2022).

56. D. Condon, M. Zhu, S. Bowring, W. Wang, A. Yang, Y. Jin, U-Pb ages from the neoproterozoic Doushantuo Formation China. *Science* **308**, 95–98 (2005).

57. C. Zhou, X.-H. Li, S. Xiao, Z. Lan, Q. Ouyang, C. Guan, Z. Chen, A new SIMS zircon U-Pb date from the Ediacaran Doushantuo Formation: Age constraint on the Weng'an biota. *Geol. Mag.* **154**, 1193–1201 (2017).

58. J. W. Hagadorn, S. Xiao, P. C. J. Donoghue, S. Bengtson, N. J. Gostling, M. Pawlowska, E. C. Raff, R. A. Raff, F. R. Turner, Y. Chongyu, C. Zhou, X. Yuan, M. B. McFeely, M. Stampanoni, K. H. Nealson, Cellular and subcellular structure of neoproterozoic animal embryos. *Science* **314**, 291–294 (2006).

59. F. S. Dunn, C. G. Kenchington, L. A. Parry, J. W. Clark, R. S. Kendall, P. R. Wilby, A crown-group cnidarian from the Ediacaran of Charnwood Forest, UK. *Nat. Ecol. Evol.* **6**, 1095–1104 (2022).

60. T. Shao, H. Tang, Y. Liu, D. Waloszek, A. Maas, H. Zhang, Diversity of cnidarians and cycloneuralians in the Fortunian (early Cambrian) Kuanchuanpu Formation at Zhangjiagou, South China. *J. Paleo.* **92**, 115–129 (2018).

61. Y. Liu, E. Carlisle, H. Zhang, B. Yang, M. Steiner, T. Shao, B. Duan, F. Marone, S. Xiao, P. C. J. Donoghue, Saccorhytus is an early ecdysozoan and not the earliest deuterostome. *Nature* **609**, 541–546 (2022).

62. J. J. Matthews, A. G. Liu, C. Yang, D. McIlroy, B. Levell, D. J. Condon, A chronostratigraphic framework for the rise of the Ediacaran macrobiota: New constraints from Mistaken Point Ecological Reserve, Newfoundland. *Geol. Soc. Am. Bull.* **133**, 612–624 (2021).

63. M. J. Benton, P. C. J. Donoghue, R. J. Asher, M. Friedman, T. J. Near, J. Vinther, Constraints on the timescale of animal evolutionary history. *Palaeon. Electron.* **18**, 1–107 (2015).

64. S. R. Noble, D. J. Condon, J. N. Carney, P. R. Wilby, T. C. Pharaoh, T. D. Ford, U-Pb geochronology and global context of the Charnian Supergroup, UK: Constraints on the age of key Ediacaran fossil assemblages. *Geol. Soc. Am. Bull.* **127**, 250–265 (2015).

65. P. R. Wilby, J. N. Carney, M. P. A. Howe, A rich Ediacaran assemblage from eastern Avalonia: Evidence of early widespread diversity in the deep ocean. *Geology* **39**, 655–658 (2011).

66. J. A. Cunningham, K. Vargas, Z. Yin, S. Bengtson, P. C. J. Donoghue, The Weng'an biota (Doushantuo Formation): An Ediacaran window on soft-bodied and multicellular microorganisms. *J. Geol. Soc. London* **174**, 793–802 (2017).

67. B. Runnegar, Following the logic behind biological interpretations of the Ediacaran biotas. *Geol. Mag.* **159**, 1093–1117 (2022).

68. N. J. Butterfield, Hooking some stem-group "worms": Fossil lophotrochozoans in the Burgess Shale. *Bioessays* **28**, 1161–1166 (2006).

69. N. J. Butterfield, T. H. P. Harvey, Small carbonaceous fossils (SCFs): A new measure of early Paleozoic paleobiology. *Geology* **40**, 71–74 (2012).

70. B. Slater, M. S. Bohlin, Animal origins: The record from organic microfossils. *Earth Sci. Rev.* **232**, 104107 (2022).

71. B. Runnegar, Muscle scars, shell form and torsion in Cambrian and Ordovician univalved mollusks. *Lethaia* **14**, 311–322 (1981).

72. S. C. Peng, L. E. Babcock, P. Ahlberg, F. M. Gradstein, J. G. Ogg, M. D. Schmitz, G. M. Ogg, Chapter 19 - The Cambrian Period in *Geologic Time Scale* 2020 (Elsevier, 2020), pp. 565–629.

73. J. Inoue, P. C. J. Donoghue, Z. Yang, The impact of the representation of fossil calibrations on bayesian estimation of species divergence times. *Syst. Biol.* **59**, 74–89 (2010).

74. R. C. M. Warnock, Z. Yang, P. C. J. Donoghue, Exploring uncertainty in the calibration of the molecular clock. *Biol. Lett.* **8**, 156–159 (2012).

75. J. Barba-Montoya, M. dos Reis, Z. Yang, Comparison of different strategies for using fossil calibrations to generate the time prior in Bayesian molecular clock dating. *Mol. Phylogenet. Evol.* **114**, 386–400 (2017).

76. C. Nielsen, *Animal Evolution: Interrelationships of the Living Phyla* (Oxford Univ. Press, 2011).

77. G. Giribet, G. D. Edgecombe, *The Invertebrate Tree of Life* (Princeton Univ. Press, 2020).

78. S. H. Xiao, G. M. Narbonne, The Ediacaran Period in *Geologic Time Scale 2020* (2020), pp. 521–561.

79. G. Halverson, S. Porter, G. Shields, The Tonian and Cryogenian Periods in *Geologic Time Scale 2020* (2020), pp. 495–519.

80. M. S. Y. Lee, J. Soubrier, G. D. Edgecombe, Rates of lution during the Cambrian explosion. *Curr. Biol.* **23**, 1889–1895 (2013).

81. G. A. Wray, Molecular clocks and the early evolution of metazoan nervous systems. *Philos. Trans. R. Soc. Lond. B Biol. Sci.* **370**, 20150046 (2015).

82. E. A. Sperling, R. G. Stockey, The temporal and environmental context of early animal evolution: Considering all the ingredients of an “explosion”. *Integr. Comp. Biol.* **58**, 605–622 (2018).

83. X.-P. Dong, H. Zhang, Middle Cambrian through lowermost Ordovician conodonts from Hunan, South China. *J. Paleo.* **91**, 1–89 (2017).

84. B. Runnegar, A molecular-clock date for the origin of the animal phyla. *Lethaia* **15**, 199–205 (1982).

85. G. A. Wray, J. S. Levinton, L. H. Shapiro, Molecular evidence for deep precambrian divergences among metazoan phyla. *Science* **274**, 568–573 (1996).

86. N. Nikoh, N. Iwabe, K.-i. Kuma, M. Ohno, T. Sugiyama, Y. Watanabe, K. Yasui, S.-c. Zhang, K. Hori, Y. Shimura, T. Miyata, An estimate of divergence time of parazoa and eumetazoa and that of cephalochordata and vertebrata by aldolase and triose phosphate isomerase clocks. *J. Mol. Evol.* **45**, 97–106 (1997).

87. D. Y. C. Wang, S. Kumar, S. B. Hedges, Divergence time estimates for the early history of animal phyla and the origin of plants, animals and fungi. *Proc. Biol. Sci.* **266**, 163–171 (1999).

88. S. B. Hedges, J. E. Blair, M. L. Venturi, J. L. Shoe, A molecular timescale of eukaryote evolution and the rise of complex multicellular life. *BMC Evol. Biol.* **4**, 2 (2004).

89. D. Graur, W. Martin, Reading the entrails of chickens: Molecular timescales of evolution and the illusion of precision. *Trends Genet.* **20**, 80–86 (2004).

90. S. Shaul, D. Graur, Playing chicken (Gallus gallus): Methodological inconsistencies of molecular divergence date estimates due to secondary calibration points. *Gene* **300**, 59–61 (2002).

91. M. J. Benton, P. C. J. Donoghue, R. J. Asher, Calibrating and constraining molecular clocks in *Timetree of life*, S. B. Hedges, S. Kumar, Eds. (Oxford Univ. Press, 2009), pp. 1268.

92. K. J. Peterson, J. B. Lyons, K. S. Nowak, C. M. Takacs, M. J. Wargo, M. A. McPeek, Estimating metazoan divergence times with a molecular clock. *Proc. Natl. Acad. Sci. U.S.A.* **101**, 6536–6541 (2004).

93. K. J. Peterson, J. A. Cotton, J. G. Gehling, D. Pisani, The Ediacaran emergence of bilaterians: Congruence between the genetic and the geological fossil records. *Philos. Trans. R. Soc. Lond. B Biol. Sci.* **363**, 1435–1443 (2008).

94. S. Xiao, A. D. Muscente, L. Chen, C. Zhou, J. D. Schiffbauer, A. D. Wood, N. F. Polys, X. Yuan, The Weng'an biota and the Ediacaran radiation of multicellular eukaryotes. *Natl. Sci. Rev.* **1**, 498–520 (2014).

95. C. R. Marshall, Using the fossil record to evaluate timetree timescales. *Front. Genet.* **10**, 1049 (2019).

96. C. R. Marshall, Five palaeobiological laws needed to understand the evolution of the living biota. *Nat. Ecol. Evol.* **1**, 0165 (2017).

97. J. F. Parham, P. C. J. Donoghue, C. J. Bell, T. D. Calway, J. J. Head, P. A. Holroyd, J. G. Inoue, R. B. Irmis, W. G. Joyce, D. T. Ksepka, J. S. L. Patane, N. D. Smith, J. E. Tarver, M. van Tuinen, Z. Yang, K. D. Angielczyk, J. M. Greenwood, C. A. Hipsley, L. Jacobs, P. J. Makovicky, J. Muller, K. T. Smith, J. M. Theodor, R. C. M. Warnock, M. J. Benton, Best practices for justifying fossil calibrations. *Syst. Biol.* **61**, 346–359 (2012).

98. G. E. Budd, S. Jensen, The origin of the animals and a 'Savannah' hypothesis for early bilaterian evolution. *Biol. Rev.* **92**, 446–473 (2017).

99. Z. Yin, W. Sun, P. Liu, J. Chen, D. J. Bottjer, J. Li, M. Zhu, Diverse and complex developmental mechanisms of early Ediacaran embryo-like fossils from the Weng'an biota, southwest China. *Philos. Trans. R. Soc. Lond. B Biol. Sci.* **377**, 20210032 (2022).

100. W. Sun, Z. Yin, P. Liu, M. Zhu, P. Donoghue, Developmental biology of *Spiralicellula* and the Ediacaran origin of crown metazoans. *Proc.Biol. Sci.* **291**, 20240101 (2024).

101. P. F. Hoffman, D. S. Abbot, Y. Ashkenazy, D. I. Benn, J. J. Brocks, P. A. Cohen, G. M. Cox, J. R. Creveling, Y. Donnadieu, D. H. Erwin, I. J. Fairchild, D. Ferreira, J. C. Goodman, G. P. Halverson, M. F. Jansen, G. Le Hir, G. D. Love, F. A. Macdonald, A. C. Maloof, C. A. Partin, G. Ramstein, B. E. J. Rose, C. V. Rose, P. M. Sadler, E. Tziperman, A. Voigt, S. G. Warren, Snowball Earth climate dynamics and Cryogenian geology-geobiology. *Sci. Adv.* **3**, e1600983 (2017).

102. A. D. Rooney, J. V. Strauss, A. D. Brandon, F. A. Macdonald, A Cryogenian chronology: Two long-lasting synchronous Neoproterozoic glaciations. *Geology* **43**, 459–462 (2015).

103. H. J. Griffiths, R. J. Whittle, E. G. Mitchell, Animal survival strategies in Neoproterozoic ice worlds. *Glob. Chang. Biol.* **29**, 10–20 (2023).

104. S. C. Dufour, D. McIlroy, Ediacaran pre-placozoan diploblasts in the Avalonian biota: The role of chemosynthesis in the evolution of early animal life. *Geol. Soc. Lond. Spec. Publ.* **448**, 211–219 (2018).

105. J. P. Pu, S. A. Bowring, J. Ramezani, P. Myrow, T. D. Raub, E. Landing, A. Mills, E. Hodgin, F. A. Macdonald, Dodging snowballs: Geochronology of the Gaskiers glaciation and the first appearance of the Ediacaran biota. *Geology* **44**, 955–958 (2016).

106. J. R. Nursall, Oxygen as a prerequisite to the origin of the Metazoa. *Nature* **183**, 1170–1172 (1959).

107. P. E. Cloud Jr., Atmospheric and hydrospheric evolution on the primitive earth: Both secular accretion and biological and geochemical processes have affected earth's volatile envelope. *Science* **160**, 729–736 (1968).

108. N. J. Planavsky, D. Asael, A. Hofmann, C. T. Reinhard, S. V. Lalonde, A. Knudsen, X. Wang, F. Ossa Ossa, E. Pecoits, A. J. B. Smith, N. J. Beukes, A. Bekker, T. M. Johnson, K. O. Konhauser, T. W. Lyons, O. J. Rouxel, Evidence for oxygenic photosynthesis half a billion years before the Great Oxidation Event. *Nat. Geosci.* **7**, 283–286 (2014).

109. C. T. Reinhard, N. J. Planavsky, S. L. Olson, T. W. Lyons, D. H. Erwin, Earth's oxygen cycle and the evolution of animal life. *Proc. Natl. Acad. Sci. U.S.A.* **113**, 8933–8938 (2016).

110. N. J. Butterfield, Oxygen, animals and aquatic bioturbation: An updated account. *Geobiology* **16**, 3–16 (2018).

111. E. U. Hammarlund, Harnessing hypoxia as an evolutionary driver of complex multicellularity. *Interface Focus* **10**, 20190101 (2020).

112. R. G. Stockey, D. B. Cole, U. C. Farrell, H. Agić, T. H. Boag, J. J. Brocks, D. E. Canfield, M. Cheng, P. W. Crockford, H. Cui, T. W. Dahl, L. Del Mouro, K. Dewing, S. Q. Dornbos, J. F. Emmings, R. R. Gaines, T. M. Gibson, B. C. Gill, G. J. Gilleaudeau, K. Goldberg, R. Guilbaud, G. Halverson, E. U. Hammarlund, K. Hantsoo, M. A. Henderson, C. M. Henderson, M. S. W. Hodgskiss, A. J. M. Jarrett, D. T. Johnston, P. Kabanov, J. Kimmig, A.

H. Knoll, M. Kunzmann, M. A. LeRoy, C. Li, D. K. Loydell, F. A. Macdonald, J. M. Magnall, N. T. Mills, L. M. Och, B. O'Connell, A. Pagès, S. E. Peters, S. M. Porter, S. W. Poulton, S. R. Ritzer, A. D. Rooney, S. Schoepfer, E. F. Smith, J. V. Strauss, G. J. Uhlein, T. White, R. A. Wood, C. R. Woltz, I. Yurchenko, N. J. Planavsky, E. A. Sperling, Sustained increases in atmospheric oxygen and marine productivity in the Neoproterozoic and Palaeozoic eras. *Nat. Geosci.* **17**, 667–674 (2024).

113. R. Wood, A. G. Liu, F. Bowyer, P. R. Wilby, F. S. Dunn, C. G. Kenchington, J. F. H. Cuthill, E. G. Mitchell, A. Penny, Integrated records of environmental change and evolution challenge the Cambrian explosion. *Nat. Ecol. Evol.* **3**, 528–538 (2019).

114. B. J. W. Mills, A. J. Krause, I. Jarvis, B. D. Cramer, Evolution of atmospheric O2 through the Phanerozoic, revisited. *Annu. Rev. Earth Planet. Sci.* **51**, 253–276 (2023).

115. A. D. Rooney, M. D. Cantine, K. D. Bergmann, I. Gomez-Perez, B. Al Baloushi, T. H. Boag, J. F. Busch, E. A. Sperling, J. V. Strauss, Calibrating the coevolution of Ediacaran life and environment. *Proc. Natl. Acad. Sci. U.S.A.* **117**, 16824–16830 (2020).

116. L. Yuan, Y. Zhou, X. Chen, M. Zhu, S. W. Poulton, Z. Tian, D. Li, M. Thirlwall, G. A. Shields, Multiple ocean oxygenation events during the Ediacaran Period: Mo isotope evidence from the Nanhua Basin, South China. *Precambrian Res.* **388**, 107004 (2023).

117. J. F. Busch, E. B. Hodgin, A.-S. C. Ahm, J. M. Husson, F. A. Macdonald, K. D. Bergmann, J. A. Higgins, J. V. Strauss, Global and local drivers of the Ediacaran Shuram carbon isotope excursion. *Earth Planet. Sci. Lett.* **579**, 117368 (2022).

118. H. Fan, S. G. Nielsen, J. D. Owens, M. Auro, Y. Shu, D. S. Hardisty, T. J. Horner, C. N. Bowman, S. A. Young, H. Wen, Constraining oceanic oxygenation during the Shuram excursion in South China using thallium isotopes. *Geobiology* **18**, 348–365 (2020).

119. G.-Y. Wei, J. Wang, N. J. Planavsky, M. Zhao, E. W. Bolton, L. Jiang, D. Asael, W. Wei, H.-F. Ling, On the origin of Shuram carbon isotope excursion in South China and its implication for Ediacaran atmospheric oxygen levels. *Precambrian Res.* **375**, 106673 (2022).

120. F. Zhang, S. Xiao, S. J. Romaniello, D. Hardisty, C. Li, V. Melezhik, B. Pokrovsky, M. Cheng, W. Shi, T. M. Lenton, A. D. Anbar, Global marine redox changes drove the rise and fall of the Ediacara biota. *Geobiology* **17**, 594–610 (2019).

121. D. B. Mills, D. E. Canfield, Oxygen and animal evolution: Did a rise of atmospheric oxygen trigger the origin of animals? *Bioessays* **36**, 1145–1155 (2014).

122. E. A. Sperling, C. A. Frieder, A. V. Raman, P. R. Girguis, L. A. Levin, A. H. Knoll, Oxygen, ecology, and the Cambrian radiation of animals. *Proc. Natl. Acad. Sci. U.S.A.* **110**, 13451–13446 (2013).

123. B. Rannala, Z. Yang, Inferring speciation times under an episodic molecular clock. *Syst. Biol.* **56**, 453–466 (2007).

124. L. A. Hug, A. J. Roger, The impact of fossils and taxon sampling on ancient molecular dating analyses. *Mol. Biol. Evol.* **24**, 1889–1897 (2007).

125. E. Carlisle, C. M. Janis, D. Pisani, P. C. J. Donoghue, D. Silvestro, A timescale for placental mammal diversification based on Bayesian modeling of the fossil record. *Curr. Biol.* **33**, 3073–3082.e3 (2023).

126. Z. Yang, PAML 4: Phylogenetic analysis by maximum likelihood. *Mol. Biol. Evol.* **24**, 1586–1591 (2007).

127. H. Philippe, R. Derelle, P. Lopez, K. Pick, C. Borchiellini, N. Boury-Esnault, J. Vacelet, E. Renard, E. Houliston, E. Queinnec, C. Da Silva, P. Wincker, H. Le Guyader, S. Leys, D. J. Jackson, F. Schreiber, D. Erpenbeck, B. Morgenstern, G. Worheide, M. Manuel, Phylogenomics revives traditional views on deep animal relationships. *Curr. Biol.* **19**, 706–712 (2009).

128. C. W. Dunn, A. Hejnol, D. Q. Matus, K. Pang, W. E. Browne, S. A. Smith, E. Seaver, G. W. Rouse, M. Obst, G. D. Edgecombe, M. V. Sorensen, S. H. D. Haddock, A. Schmidt-Rhaesa, A. Okusu, R. M. Kristensen, W. C. Wheeler, M. Q. Martindale, G. Giribet, Broad

phylogenomic sampling improves resolution of the animal tree of life. *Nature* **452**, 745–749 (2008).

129. A. Hejnol, M. Obst, A. Stamatakis, M. Ott, G. W. Rouse, G. D. Edgecombe, P. Martinez, J. Baguna, X. Bailly, U. Jondelius, M. Wiens, W. E. G. Muller, E. Seaver, W. C. Wheeler, M. Q. Martindale, G. Giribet, C. W. Dunn, Assessing the root of bilaterian animals with scalable phylogenomic methods. *Proc. Biol. Sci.* **276**, 4261–4270 (2009).

130. J. F. Ryan, K. Pang, C. E. Schnitzler, A.-D. Nguyen, R. T. Moreland, D. K. Simmons, B. J. Koch, W. R. Francis, P. Havlak, NISC Comparative Sequencing Program, S. A. Smith, N. H. Putnam, S. H. D. Haddock, C. W. Dunn, T. G. Wolfsberg, J. C. Mullikin, M. Q. Martindale, A. D. Baxevanis, The genome of the ctenophore *Mnemiopsis leidyi* and its implications for cell type evolution. *Science* **342**, 1242592 (2013).

131. L. L. Moroz, K. M. Kocot, M. R. Citarella, S. Dosung, T. P. Norekian, I. S. Povolotskaya, A. P. Grigorenko, C. Dailey, E. Berezikov, K. M. Buckley, A. Ptitsyn, D. Reshetov, K. Mukherjee, T. P. Moroz, Y. Bobkova, F. Yu, V. V. Kapitonov, J. Jurka, Y. V. Bobkov, J. J. Swore, D. O. Girardo, A. Fodor, F. Gusev, R. Sanford, R. Bruders, E. Kittler, C. E. Mills, J. P. Rast, R. Derelle, V. V. Solovyev, F. A. Kondrashov, B. J. Swalla, J. V. Sweedler, E. I. Rogaev, K. M. Halanych, A. B. Kohn, The ctenophore genome and the evolutionary origins of neural systems. *Nature* **510**, 109–114 (2014).

132. N. V. Whelan, K. M. Kocot, L. L. Moroz, K. M. Halanych, Error, signal, and the placement of Ctenophora sister to all other animals. *Proc. Natl. Acad. Sci. U.S.A.* **112**, 5773–5778 (2015).

133. D. Pisani, W. Pett, M. Dohrmann, R. Feuda, O. Rota-Stabelli, H. Philippe, N. Lartillot, G. Wörheide, Genomic data do not support comb jellies as the sister group to all other animals. *Proc. Natl. Acad. Sci. U.S.A.* **112**, 15402–15407 (2015).

134. K. S. Pick, H. Philippe, F. Schreiber, D. Erpenbeck, D. J. Jackson, P. Wrede, M. Wiens, A. Alie, B. Morgenstern, M. Manuel, G. Worheide, Improved phylogenomic taxon sampling noticeably affects nonbilaterian relationships. *Mol. Biol. Evol.* **27**, 1983–1987 (2010).

135. H. Philippe, H. Brinkmann, D. V. Lavrov, D. T. J. Littlewood, M. Manuel, G. Wörheide, D. Baurain, Resolving difficult phylogenetic questions: Why more sequences are not enough. *PLoS Biol.* **9**, e1000602 (2011).

136. M. Giacomelli, M. E. Rossi, J. Lozano-Fernandez, R. Feuda, D. Pisani, Resolving tricky nodes in the tree of life through amino acid recoding. *iScience* **25**, 105594 (2022).

137. A. K. Redmond, A. McLysaght, Evidence for sponges as sister to all other animals from partitioned phylogenomics with mixture models and recoding. *Nat. Commun.* **12**, 1783 (2021).

138. P. Kapli, M. J. Telford, Topology-dependent asymmetry in systematic errors affects phylogenetic placement of Ctenophora and Xenacoelomorpha. *Sci. Adv.* **6**, eabc5162 (2020).

139. J. T. Cannon, B. C. Vellutini, J. Smith, F. Ronquist, U. Jondelius, A. Hejnol, Xenacoelomorpha is the sister group to Nephrozoa. *Nature* **530**, 89–93 (2016).

140. H. Philippe, A. J. Poustka, M. Chiodin, K. J. Hoff, C. Dessimoz, B. Tomiczek, P. H. Schiffer, S. Muller, D. Domman, M. Horn, H. Kuhl, B. Timmermann, N. Satoh, T. Hikosaka-Katayama, H. Nakano, M. L. Rowe, M. R. Elphick, M. Thomas-Chollier, T. Hankeln, F. Mertes, A. Wallberg, J. P. Rast, R. R. Copley, P. Martinez, M. J. Telford, Mitigating anticipated effects of systematic errors supports sister-group relationship between Xenacoelomorpha and Ambulacraria. *Curr. Biol.* **29**, 1818–1826.E6 (2019).

141. P. O. Mulhair, C. G. P. McCarthy, K. Siu-Ting, C. J. Creevey, M. J. O'Connell, Filtering artifactual signal increases support for Xenacoelomorpha and Ambulacraria sister relationship in the animal tree of life. *Curr. Biol.* **32**, 5180–5188.E3 (2022).

142. P. Kapli, P. Natsidis, D. J. Leite, M. Fursman, N. Jeffrie, I. A. Rahman, H. Philippe, R. R. Copley, M. J. Telford, Lack of support for Deuterostomia prompts reinterpretation of the first Bilateria. *Sci. Adv.* **7**, eabe2741 (2021).

143. K. J. Peterson, D. J. Eernisse, The phylogeny, evolutionary developmental biology, and paleobiology of the Deuterostomia: 25 years of new techniques, new discoveries, and new ideas. *Org. Divers. Evol.* **16**, 401–418 (2016).

144. S. Q. Le, O. Gascuel, An improved general amino acid replacement matrix. *Mol. Biol. Evol.* **25**, 1307–1320 (2008).

145. R. C. M. Warnock, Z. Yang, P. C. J. Donoghue, Testing the molecular clock using mechanistic models of fossil preservation and molecular evolution. *Proc. R. Soc. B Biol. Sci.* **284**, 20170227 (2017).

146. H. Li, S. Lu, W. Su, Z. Xiang, H. Zhou, Y. Zhang, Recent advances in the study of the Mesoproterozoic geochronology in the North China Craton. *J. Asian Earth Sci.* **72**, 216–227 (2013).

147. H. C. Betts, M. N. Puttick, J. W. Clark, T. A. Williams, P. C. J. Donoghue, D. Pisani, Integrated genomic and fossil evidence illuminates life's early evolution and eukaryote origin. *Nat. Ecol. Evol.* **2**, 1556–1562 (2018).

148. F. Tang, C. Yin, S. Bengtson, P. Liu, Z. Wang, L. Gaio, Octoradiate spiral organisms in the Ediacaran of South China. *Acta Geol. Sin.* **82**, 27–34 (2008).

149. J. H. Wei, X. Yin, P. V. Welander, Sterol synthesis in diverse bacteria. *Front. Microbiol.* **7**, 990 (2016).

150. E. A. Sperling, J. Vinther, A placozoan affinity for *Dickinsonia* and the evolution of late Proterozoic metazoan feeding modes. *Evol. Dev.* **12**, 201–209 (2010).

151. K. M. Towe, S. Bengtson, M. A. Fedonkin, H. J. Hofmann, C. Mankiewicz, B. N. Runnegar, Proterozoic and earliest Cambrian carbonaceous remains, trace and body fossils in *The Proterozoic Biosphere: A Multidisciplinary Study*, J. W. Schopf, C. Klein, Eds. (Cambridge Univ. Press, 1992), pp. 343–424.

152. J. Vinther, L. A. Parry, Bilateral jaw elements in *Amiskwia sagittiformis* bridge the morphological gap between gnathiferans and chaetognaths. *Curr. Biol.* **29**, 881–888.e1 (2019).

153. J. B. Caron, B. Cheung, Amiskwia is a large Cambrian gnathiferan with complex gnathostomulid-like jaws. *Commun. Biol.* **2**, 164 (2019).

154. A. Y. Zhuravlev, R. A. Wood, A. M. Penny, Ediacaran skeletal metazoan interpreted as a lophophorate. *Proc. Biol. Sci.* **282**, 20151860 (2015).

155. A. J. Shore, R. A. Wood, I. B. Butler, A. Y. Zhuravlev, S. McMahon, A. Curtis, F. T. Bowyer, Ediacaran metazoan reveals lophotrochozoan affinity and deepens root of Cambrian explosion. *Sci. Adv.* **7**, eabf2933 (2021).

156. H. Chen, L. A. Parry, J. Vinther, D. Zhai, X. Hou, X. Ma, A Cambrian crown annelid reconciles phylogenomics and the fossil record. *Nature* **583**, 249–252 (2020).

157. M. J. Benton, P. C. J. Donoghue, R. J. Asher, Calibrating and constraining molecular clocks in *The Timetree of Life*, S. B. Hedges, S. Kumar, Eds. (Cambridge Univ. Press, 2009), pp. 35–86.

158. D. M. Rohr, E. A. Measures, W. D. Boyce, I. Knight, Early Ordovician gastropods of the Barbace Cove Member (Boat Harbour Formation) and Catoche Formation, Western Newfoundland. *Curr. Res. Newfoundland Dep. Mines Energy* **2001**, 113–126 (2001).

159. R. J. Howard, M. Giacomelli, J. Lozano-Fernandez, G. D. Edgecombe, J. F. Fleming, R. M. Kristensen, X. Ma, J. Olesen, M. V. Sørensen, P. F. Thomsen, M. A. Wills, P. C. J. Donoghue, D. Pisani, The Ediacaran origin of Ecdysozoa: Integrating fossil and phylogenomic data. *J. Geol. Soc. London* **179**, jgs2021–jgs2107 (2022).

160. J. M. Wolfe, A. C. Daley, D. A. Legg, G. D. Edgecombe, Fossil calibrations for the arthropod tree of life. *Earth Sci. Rev.* **160**, 43–110 (2016).

161. G. E. Budd, S. Jensen, A critical reappraisal of the fossil record of bilaterian phyla. *Biol. Rev.* **74**, 253–295 (2000).

162. G. M. Narbonne, H. J. Hofmann, Ediacaran biota of the Wernecke Mountains, Yukon, Canada. *Palaeontology* **30**, 647–676 (1987).

163. C. E. Isachsen, S. A. Bowring, E. Landing, S. D. Samson, New constraint on the division of Cambrian time. *Geology* **22**, 496–498 (1994).

164. X.-g. Zhang, D. J. Siveter, D. Waloszek, A. Maas, An epipodite-bearing crown-group crustacean from the Lower Cambrian. *Nature* **449**, 595–598 (2007).

165. X.-g. Zhang, A. Maas, J. T. Haug, D. J. Siveter, D. Waloszek, A eucrustacean metanauplius from the Lower Cambrian. *Curr. Biol.* **20**, 1075–1079 (2010).

166. J. Olesen, Phylogeny of Branchiopoda (Crustacea) - character evolution and contribution of uniquely preserved fossils. *Arthro. System. Phylo.* **67**, 3–39 (2009).

167. A. T. Nielsen, T. Weidner, F. Terfelt, M. Høyberget, Upper Cambrian (Furongian) biostratigraphy in Scandinavia revisited: Definition of superzones. *GFF* **136**, 193–197 (2014).

168. P. Greenslade, P. Whalley, R. Dallai, in *Second International Seminar on Apterygota*. (Univ. of Siena, 1986), pp. 319–323.

169. M. S. Engel, D. A. Grimaldi, New light shed on the oldest insect. *Nature* **427**, 627–630 (2004).

170. A. Nel, J. Prokop, P. Nel, P. Grandcolas, D. Y. Huang, P. Roques, E. Guilbert, O. Dostal, J. Szwedo, Traits and evolution of wing venation pattern in paraneopteran insects. *J. Morphol.* **273**, 480–506 (2012).

171. C. Brauckmann, B. Brauckmann, E. Gröning, The stratigraphical position of the oldest known Pterygota (Insecta. Carboniferous, Namurian). *Annales de la Société géologique de Belgique* **117**, 47–56 (1996).

172. J. B. Jago, D. C. García-Bellido, J. G. Gehling, An early Cambrian chelicerate from the Emu Bay Shale, South Australia. *Palaeontology* **59**, 549–562 (2016).

173. D. A. Legg, M. D. Sutton, G. D. Edgecombe, Arthropod fossil data increase congruence of morphological and molecular phylogenies. *Nat. Commun.* **4**, 2485 (2013).

174. D.-G. Shu, S. C. Morris, Z.-F. Zhang, J. Han, The earliest history of the deuterostomes: The importance of the Chengjiang Fossil-Lagerstätte. *Proc. R. Soc. B Biol. Sci.* **277**, 165–174 (2010).

175. C. Yang, X.-H. Li, M. Zhu, D. J. Condon, J. Chen, Geochronological constraint on the Cambrian Chengjiang biota, South China. *J. Geol. Soc. London* **175**, 659–666 (2018).

176. S. E. Gabbott, P. C. J. Donoghue, R. S. Sansom, J. Vinther, A. Dolocan, M. A. Purnell, Pigmented anatomy in Carboniferous cyclostomes and the evolution of the vertebrate eye. *Proc. Biol. Sci.* **283**, 20161151 (2016).

177. B. J. Swalla, A. B. Smith, Deciphering deuterostome phylogeny: Molecular, morphological and palaeontological perspectives. *Philos. Trans. R. Soc. Lond. B Biol. Sci.* **363**, 1557–1568 (2008).

178. S. Clausen, A. B. Smith, Stem structure and evolution in the earliest pelmatozoan echinoderms. *Palaeontology* **82**, 737–748 (2008).

179. P. C. J. Donoghue, J. N. Keating, Early vertebrate evolution. *Palaeontology* **57**, 879–893 (2014).

180. D. J. E. Murdock, X.-P. Dong, J. E. Repetski, F. Marone, M. Stampanoni, P. C. J. Donoghue, The origin of conodonts and of vertebrate mineralized skeletons. *Nature* **502**, 546–549 (2013).

181. Y.-a. Zhu, Q. Li, J. Lu, Y. Chen, J. Wang, Z. Gai, W. Zhao, G. Wei, Y. Yu, P. E. Ahlberg, M. Zhu, The oldest complete jawed vertebrates from the early Silurian of China. *Nature* **609**, 954–958 (2022).

182. M. J. Melchin, P. M. Sadler, B. D. Cramer, The Silurian Period in *Geologic Time Scale 2020* (2020), pp. 695–732.

183. C. J. Burrow, D. Rudkin, Oldest near-complete acanthodian: The first vertebrate from the Silurian Bertie Formation Konservat-Lagerstatte, Ontario. *PLOS ONE* **9**, e104171 (2014).

184. M. Zhu, W. Zhao, L. Jia, J. Lu, T. Qiao, Q. Qu, The oldest articulated osteichthyan reveals mosaic gnathostome characters. *Nature* **458**, 469–474 (2009).

185. F. M. Gradstein, J. G. Ogg, M. Schmitz, G. Ogg, Eds., *The Geological Timescale 2012*, (Elsevier, 2012).

186. M. Ruta, M. I. Coates, D. L. J. Quicke, Early tetrapod relationships revisited. *Biol. Rev.* **78**, 251–345 (2003).

187. T. E. Meyer, J. S. Anderson, Tarsal fusion and the formation of the astragalus in *Hylonomus lyelli*, the earliest amniote, and other early tetrapods. *J. Vertebr. Paleontol.* **33**, 488–492 (2013).

188. S. Alvarez-Carretero, A. U. Tamuri, M. Battini, F. F. Nascimento, E. Carlisle, R. J. Asher, Z. Yang, P. C. J. Donoghue, M. Dos Reis, A species-level timeline of mammal evolution integrating phylogenomic data. *Nature* **602**, 263–267 (2022).

189. J. J. Flynn, J. M. Parrish, B. Rakotosamimanana, W. F. Simpson, A. R. Wyss, A Middle Jurassic mammal from Madagascar. *Nature* **401**, 57–60 (1999).

190. S. P. Hesselbo, J. G. Ogg, M. Ruhl, L. A. Hinnov, C. J. Huang, The Jurassic Period in *Geologic Time Scale 2020* (2020), pp. 955–1021.

191. M. Li, J. Ogg, Y. Zhang, C. Huang, L. Hinnov, Z.-Q. Chen, Z. Zou, Astronomical tuning of the end-Permian extinction and the Early Triassic Epoch of South China and Germany. *Earth Planet. Sci. Lett.* **441**, 10–25 (2016).

192. Z.-X. Luo, Z. Kielan-Jaworowska, R. L. Cifelli, In quest for a phylogeny of Mesozoic mammals. *Acta Palaeontol. Pol.* **47**, 1–78 (2002).

193. S. Bi, Y. Wang, J. Guan, X. Sheng, J. Meng, Three new Jurassic euharamiyidan species reinforce early divergence of mammals. *Nature* **514**, 579–584 (2014).

194. J. Wang, J. R. Wible, B. Guo, S. L. Shelley, H. Hu, S. Bi, A monotreme-like auditory apparatus in a Middle Jurassic haramiyidan. *Nature* **590**, 279–283 (2021).

195. Z. X. Luo, S. M. Gatesy, F. A. Jenkins Jr., W. W. Amaral, N. H. Shubin, Mandibular and dental characteristics of Late Triassic mammaliaform *Haramiyavia* and their ramifications for basal mammal evolution. *Proc. Natl. Acad. Sci. U.S.A.* **112**, E7101–E7109 (2015).

196. M. N. Puttick, J. E. O'Reilly, A. R. Tanner, J. F. Fleming, J. Clark, L. Holloway, J. Lozano-Fernandez, L. A. Parry, J. E. Tarver, D. Pisani, P. C. J. Donoghue, Uncertain-tree: Discriminating among competing approaches to the phylogenetic analysis of phenotype data. *Proc. Biol. Sci.* **284**, 20162290 (2017).

197. J. E. Tarver, M. dos Reis, S. Mirarab, R. J. Moran, S. Parker, J. E. O'Reilly, B. L. King, M. J. O'Connell, R. J. Asher, T. Warnow, K. J. Peterson, P. C. J. Donoghue, D. Pisani, The interrelationships of placental mammals and the limits of phylogenetic inference. *Genome Biol. Evol.* **8**, 330–344 (2016).

198. G. P. W. Mantilla, S. G. B. Chester, W. A. Clemens, J. R. Moore, C. J. Sprain, B. T. Hovatter, W. S. Mitchell, W. W. Mans, R. Mundil, P. R. Renne, Earliest Palaeocene purgatoriids and the initial radiation of stem primates. *R. Soc. Open Sci.* **8**, 210050 (2021).

199. W. A. Clemens, G. P. Wilson, Early Torrejonian mammalian local faunas from northeastern Montana, U.S.A. *Mus. North. Ariz. Bull.* **65**, 111–158 (2009).

200. R. T. Becker, J. E. A. Marshall, A. C. Da Silva, F. P. Agterberg, F. M. Gradstein, J. G. Ogg, The Devonian Period in *Geologic Time Scale 2020* (2020), pp. 733–810.

201. R. W. Gess, M. I. Coates, B. S. Rubidge, A lamprey from the Devonian period of South Africa. *Nature* **443**, 981–984 (2006).

202. S. C. Peng, L. E. Babcock, P. Ahlberg, The Cambrian Period in *Geologic Time Scale 2020* (2020), pp. 565–629.

203. A. B. Smith, Cambrian eleutherozoan echinoderms and the early diversification of edrioasteroids. *Palaeontology* **28**, 715–756 (1985).

204. D. Goldman, P. M. Sadler, S. A. Leslie, M. J. Melchin, F. P. Agterberg, F. M. Gradstein, The Ordovician Period in *Geologic Time Scale 2020* (2020), pp. 631–694.

205. A. B. Smith, J. J. Savill, *Bromidechinus*, a new Ordovician echinozoan (Echinodermata), and its bearing on the early history of echinoids. *Earth Environ. Sci. Trans. R. Soc. Edinb.* **92**, 137–147 (2001).

206. S. Bengtson, A. Urbanek, *Rhabdotubus*, a Middle Cambrian rhabdopleurid hemichordate. *Lethaia* **19**, 293–308 (1986).

207. J. Maletz, Tracing the evolutionary origins of the Hemichordata (Enteropneusta and Pterobranchia). *Palaeoworld* **28**, 58–72 (2019).

208. J. P. Botting, J. S. Peel, Early Cambrian sponges of the Sirius Passet Biota, North Greenland. *Pap. Palaeontol.* **2**, 463–487 (2016).

209. D. A. T. Harper, E. U. Hammarlund, T. P. Topper, A. T. Nielsen, J. A. Rasmussen, T.-Y. S. Park, M. P. Smith, The Sirius Passet Lagerstätte of North Greenland: A remote window on the Cambrian explosion. *J. Geol. Soc. London* **176**, 1023–1037 (2019).

210. J. P. Botting, L. A. Muir, Early sponge evolution: A review and phylogenetic framework. *Palaeoworld* **27**, 1–29 (2018).

211. J. B. Antcliffe, R. H. T. Callow, M. D. Brasier, Giving the early fossil record of sponges a squeeze. *Biol. Rev. Camb. Philos. Soc.* **89**, 972–1004 (2014).
